# Vulnerability to climate change of species in protected areas in Thailand

**DOI:** 10.1038/s41598-022-09767-9

**Published:** 2022-04-05

**Authors:** Nirunrut Pomoim, Alice C. Hughes, Yongyut Trisurat, Richard T. Corlett

**Affiliations:** 1 Center for Integrative Conservation, Xishuangbanna Tropical Botanical Garden, Chinese Academy of Sciences, Menglun, Yunnan China; 2 University of Chinese Academy of Sciences, Beijing, China; 3 Center of Conservation Biology, Core Botanical Gardens, Xishuangbanna Tropical Botanical Garden, Chinese Academy of Sciences, Menglun, Yunnan China; 4 Faculty of Forestry, Kasetsart University, Bangkok, Thailand

**Keywords:** Climate-change ecology, Ecology, Conservation biology

## Abstract

Although 23% of Thailand’s land is in protected areas, these are vulnerable to climate change. We used spatial distribution modelling for 866 vertebrate and 591 plant species to understand potential climate change impacts on species in protected areas. Most mammals, birds, and plants were projected to decline by 2070, but most amphibians and reptiles were projected to increase. By 2070 under RCP8.5, 54% of modeled species will be threatened and 11 nationally extinct. However, SDMs are sensitive to truncation of the climate space currently occupied by habitat loss and hunting, and apparent truncation by data limitations. In Thailand, lowland forest clearance has biased records for forest-dependent species to cooler uplands (> 250 m a.s.l.) and hunting has confined larger vertebrates to well-protected areas. In contrast, available data is biased towards lowland non-forest taxa for amphibians and reptiles. Niche truncation may therefore have resulted in overestimation of vulnerability for some mammal and plant species, while data limitations have likely led to underestimation of the threat to forest-dependent amphibians and reptiles. In view of the certainty of climate change but the many uncertainties regarding biological responses, we recommend regular, long-term monitoring of species and communities to detect early signals of climate change impacts.

## Introduction

The current draft of the Convention on Biological Diversity (CBD)’s Post-2020 Global Biodiversity Framework—expected to be adopted at the UN Biodiversity Conference (COP 15) Part 2 in Kunming, China, in May 2022—calls for ‘well-connected systems of protected areas and other effective area-based conservation measures’ covering at least 30% of the planet by 2030^1^. This ambitious target—increased from 17% terrestrial coverage in the 2020 targets—reflects a general recognition that well-managed protected areas (PAs) are, and will continue to be, the cornerstone of global biodiversity conservation. At the same time, it is also increasingly recognized that the fixed position of PAs makes them vulnerable to the impacts of climate change^2–4^. Despite this vulnerability, there is evidence that PAs can act as a buffer against some of the detrimental effects of climate change^5^ and that PAs and PA systems can be managed in ways that reduce these impacts^6^. Planning for the post-2020 targets must therefore start with an assessment of the climate change vulnerability of the existing PA system^7^.

Tropical East Asia supports an estimated 15–25% of global terrestrial biodiversity in only 4% of global land area^8^ and many studies have identified this region’s species and ecosystems as particularly threatened^8–11^. Unfortunately, this region, as well as most of the individual countries of which it is comprised, is relatively data-poor, with incomplete species lists, inadequate conservation assessments, and a lack of data on population sizes and trends for threatened species^12^. Conservation assessments and prioritization therefore depend heavily on the IUCN Red List global assessments, which are near-complete only for terrestrial vertebrates and gymnosperms (http://www.iucnredlist.org, accessed 27-12-2021). Despite these problems, national investments in conservation and conservation capacity have increased greatly in most of the region over the last 20 years^8^, putting the CBD’s post-2020 biodiversity targets within reach.

**Figure 1:**
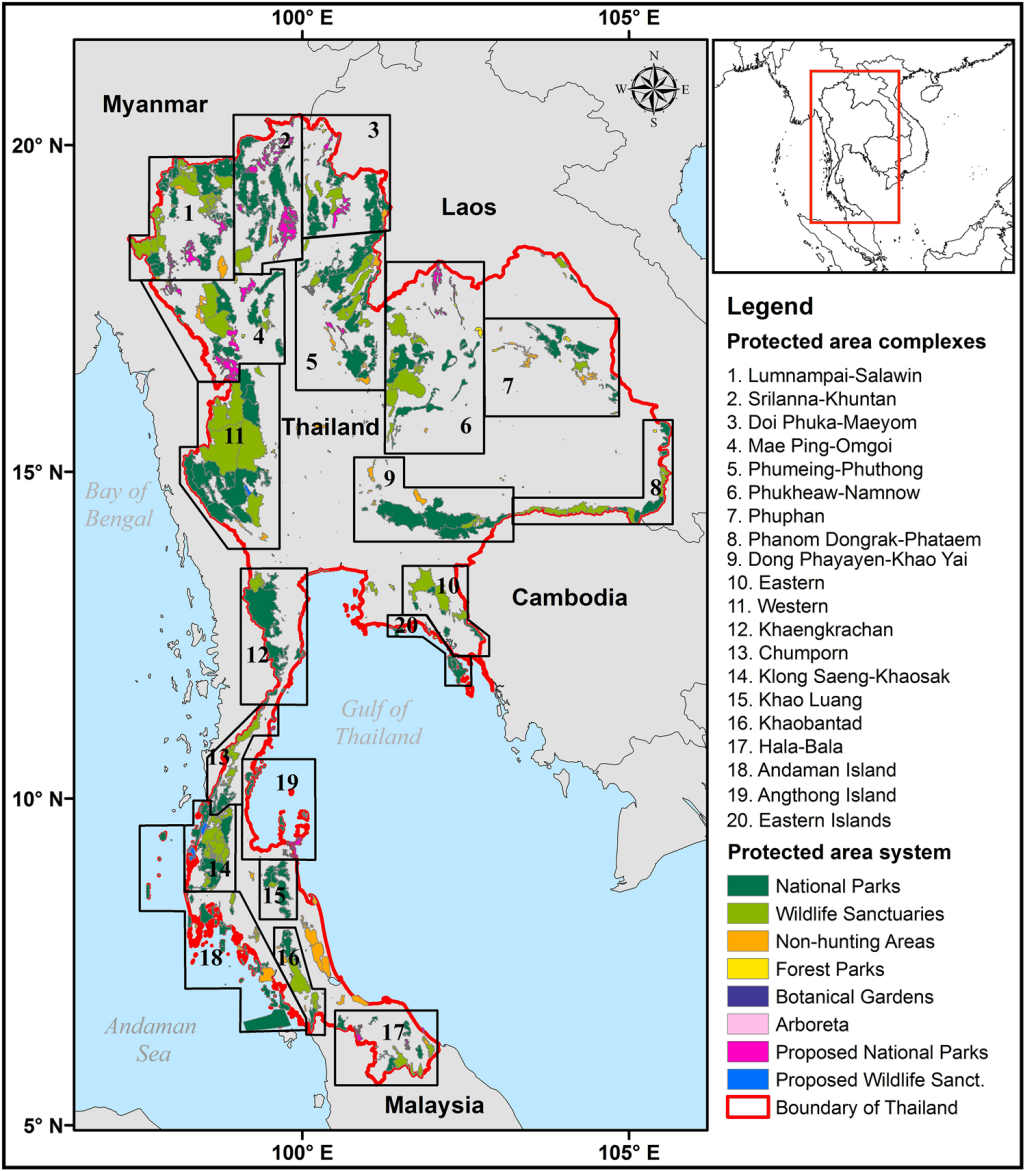
Thailand: location and protected area system, showing the protected area complexes used for management (DNP 2019). Maps created in ArcMap 10.5 (https://support.esri.com/en/products/desktop/arcgis-desktop/arcmap/10-5-1).

Thailand is in the center of the Southeast Asian region and has land borders with Laos, Cambodia, Myanmar, and Malaysia (Fig. 1). The total land area is 517,624 km^2^ and altitude ranges from 0 to 2564 m above mean sea-level. The climate is characterized by distinct wet and dry seasons associated with the Asian summer and winter monsoons, respectively. Temperature declines consistently with altitude, but rainfall has a more complex pattern, with a drier center and east, and wetter south, west, and north^7^. Thailand is entirely within the Indo-Burma biodiversity hotspot, which is recognized as one of the 36 global biodiversity hotspots (http://www.cepf.net/our-work/biodiversity-hotspots/hotspots-defined). Forest covers 31.7% of the country’s total area with 83% of this included within existing protected areas^13^. The protected area system (Fig. 1) consists of areas that fit within the IUCN’s definitions^14^, including Wildlife Sanctuaries (IUCN category Ia), National Parks (II), Non-hunting Areas (IV), and Forest Parks (V), as well as additional categories defined by the Thai government but not considered further here. There are currently 132 National Parks, 60 Wildlife Sanctuaries, 80 Non-hunting Areas, and 114 Forest Parks. The Department of National Parks, Wildlife and Plant Conservation (DNP) is also in the process of establishing at least 23 additional National Parks and 7 Non-hunting Areas. In total, these PAs cover approximately 118,320 km^2^ or 22.8% of the country’s land area. Under the Twelfth National Economic and Social Development Plan (2017–2021), the Thai government aims to increase the protected area system to 25% of the country by adding 15,796 km^2^ of forest reserves previously managed by the Royal Forest Department^15^.

Although the total PA coverage is relatively high, it is biased towards the north and west of Thailand and some ecosystem types are not adequately represented, including peat swamp forest, mangrove forest, and lowland deciduous forests. Moreover, Thailand’s PAs are highly fragmented, with only 8% larger than 1000 km^2^, with the potential to maintain populations of large mammals such as tigers, leopards, and elephants^16^. Around 54% are 100–500 km^2^ and 38% are < 100 km^2^. To reduce the problems of fragmentation, the DNP has established 17 ‘forest complexes’ which include multiple PAs, as well as three marine complexes^17^ (Fig. 1). Within these complexes, some PAs have actual or potential future connections, allowing movements of species between them^18^. Management is coordinated between individual PAs and, where possible, with other government agencies that control land. Several complexes also have potential for transboundary cooperation with PAs in neighboring countries. The most recent IUCN Red List assessment (Version 2021-3. https://www.iucnredlist.org. Accessed on 27-12-2021) lists 552 globally threatened species as occurring in Thailand, including 64 mammals, 67 birds, 42 reptiles, 12 amphibians, and 229 plants, but most plant and invertebrate species have not yet been assessed.

Protecting and rehabilitating forests is an important part of Thailand’s national climate change adaptation and mitigation strategy^19^, but the potential impacts of climate change on forest biodiversity have received less attention^20^. We have previously used a statistically derived Global Environmental Stratification (GEnS) to project changes in the distribution of bioclimatic conditions in Thailand as a whole, and within PAs, by 2050 and 2070^7^. However, bioclimatic types per se are not direct targets for conservation action, and not the only factors determining the distributions of wild species. Effective conservation planning, now and under climate change, also needs species occurrence data. Thailand has relatively good biodiversity data by tropical standards, with a half-completed Flora and good checklists for other taxa. However, accurate locality data for most taxa other than birds comes mainly from the PAs and even there is very incomplete. In this study we therefore use all the available data to assess the vulnerability of species within PAs to climate change, while also assessing the usefulness of such incomplete data for assessing vulnerability in a tropical biodiversity hotspot.

The major research questions were:

1. How complete and representative is the available biodiversity data?
2. How will patterns of species richness in Thailand change by 2070 under projected climate change?
3. How will the availability of habitat change for individual species change by 2070 under projected climate change?
4. What will be the impact of projected climate change on species within protected areas?

## Results

### Data completeness and representativeness

Taxonomic coverage varied greatly among taxa. For vertebrates, coverage largely reflected detectability in the field and many smaller species were surveyed inadequately, if at all. Within mammals, the larger-bodied ungulates, primates, and carnivores had good coverage (88, 58 and 63%, respectively, of species recorded in Thailand), including threatened species, but few bats and no insectivores were covered, and coverage of rodents and treeshrews was incomplete. Only 12% of Thai reptiles were included and only 16% of amphibians, and no threatened species of either had enough locality data (> 10 unique occurrence records) to be modeled. However, our bird dataset included 67% of Thailand’s recorded avifauna, including threatened species.

**Figure 2:**
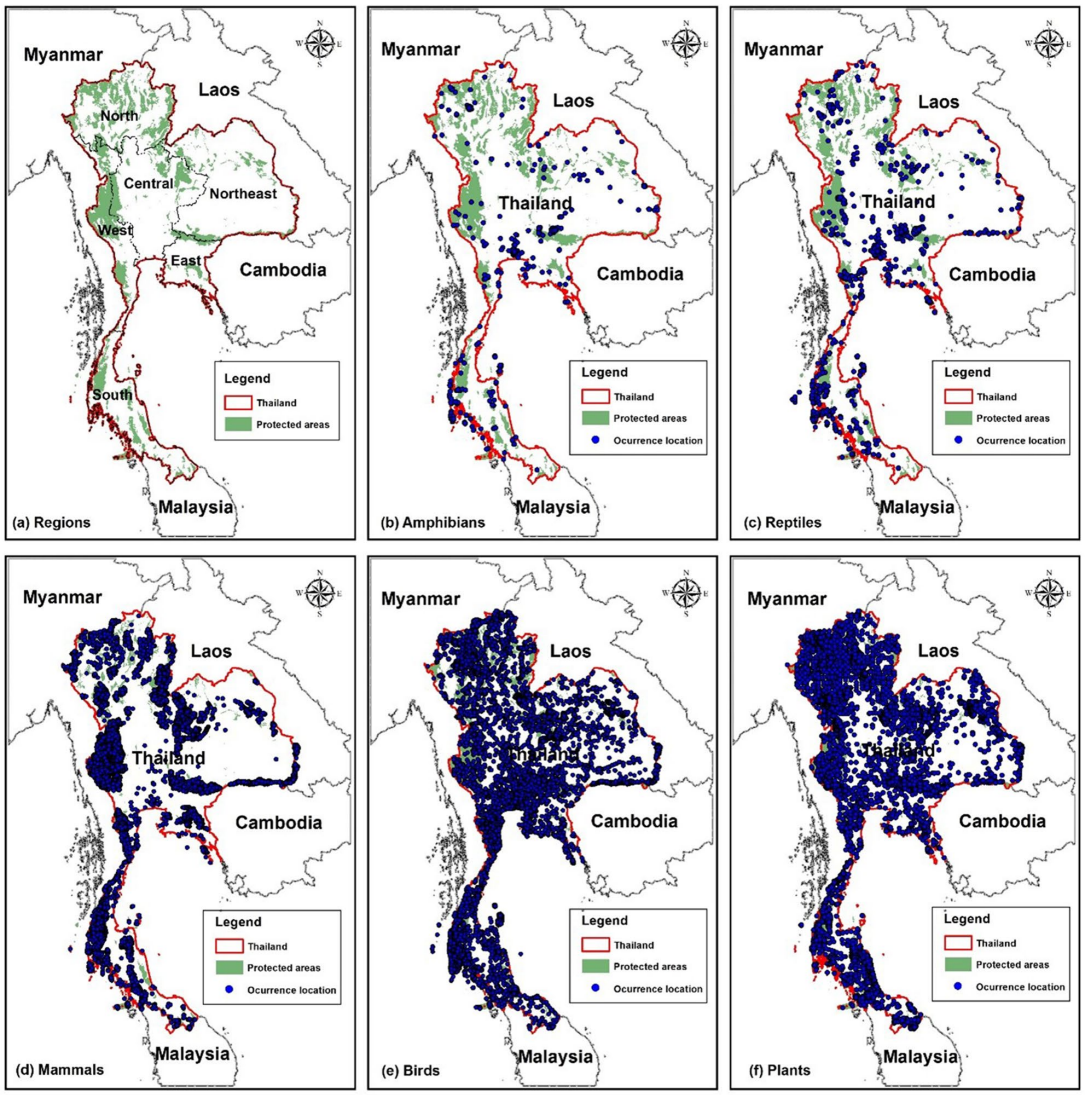
Maps of (**a**) the six regions of Thailand used in the text, and (**b**–**f**) species occurrence locations used in the analyses for each major taxon. Maps created in ArcMap 10.5 (https://support.esri.com/en/products/desktop/arcgis-desktop/arcmap/10-5-1).

Spatial coverage also varied among taxa (Fig. 2). For plants and birds, the records covered most of Thailand, although most plant records were from forests, but records for amphibians and reptiles were very patchy and those for mammals were largely forest-dependent species from the existing PAs. Nearly two-thirds of Thailand is below 250 m elevation, but only 23% of mammal records and 22% of plant records come from the lowlands, reflecting the small amount of protected forest there (Table 1). Bird records are better distributed, with 61% from the lowlands, including wetland and coastal species, while amphibian and reptile records (76% and 66% < 250 m, respectively) are dominated by non-forest species which are most common at low elevations.

**Table 1:** Numbers of species and location records used in the analyses for each taxonomic group from localities below and above 250 m above sea-level, and the percentage of the land area in each altitudinal belt protected.

Taxon	< 250 m a.s.l.		> 250 m a.s.l.	
	Number of species	Number of localities	Number of species	Number of localities
Amphibians	31	534	29	171
Reptiles	52	1147	50	580
Mammals	80	56,573	79	186,850
Birds	645	199,421	686	125,646
Plants	517	7663	580	26,777
Area (km^2^)	395,814		226,487	
Protected (%)	6.4		39.5	

### Performance of the species distribution models

The values of the continuous Boyce Index were positive for all species distribution models (SDMs), indicating consistent predictions, and all but five (of the 1457 calculated) exceeded 0.5, suggesting an acceptable performance. Medians (and ranges) for the major taxa were mammals 0.79 (0.49–1.0), birds 0.69 (0.46–1.0), reptiles 0.69 (0.31–1.0), amphibians 0.77 (0.45–0.96), and plants 0.80 (0.70–1.0).

### Changes in projected species richness under climate change

**Figure 3:**
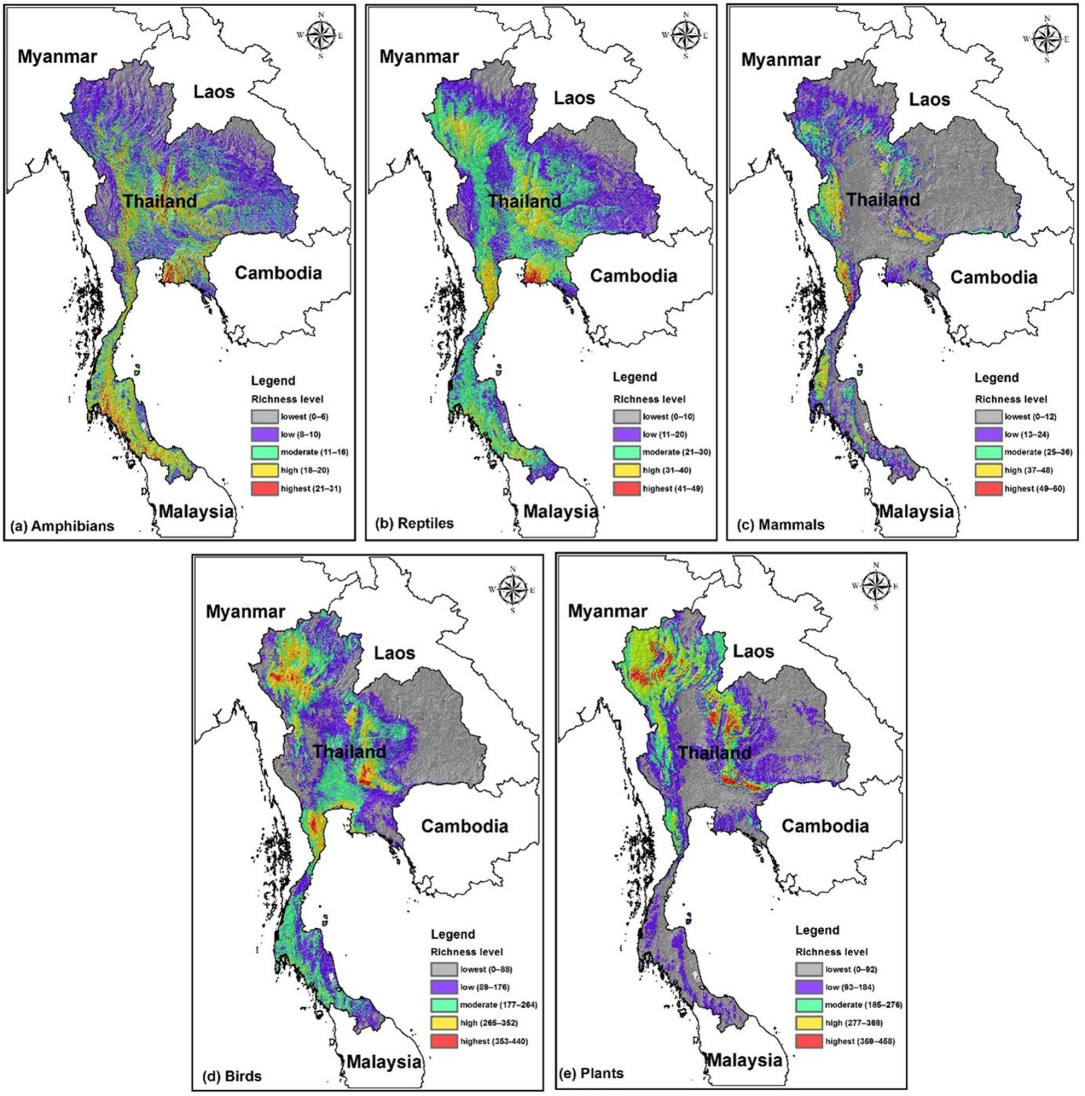
Spatial patterns of predicted species richness levels for each major taxon under current conditions. Maps created in ArcMap 10.5 (https://support.esri.com/en/products/desktop/arcgis-desktop/arcmap/10-5-1).

For the species in our dataset, maximum current richness in a 1 × 1 km grid cell, estimated from stacked single-species SDMs, is 31 species of amphibians, 49 reptiles, 60 mammals, 440 birds, and 458 plants (Table 2). The grid cells with the highest estimated current richness are concentrated in the Central, East, and South regions for amphibians, East for reptiles, West, Central, and Northeast for mammals, and North for birds and plants (Table 2; Fig. 3). Projections for 2070 show marked declines in species richness for most taxa under all scenarios, with mammals showing the largest declines (Table 2). Maximum species richness declined for all taxa and the numbers of grid cells in the highest current richness class declined for mammals, birds, and plants. Reductions were generally most severe under RCP8.5. Spatial patterns of richness also changed, although the change in pattern was different for each taxon (Table 2; Supplementary Material Figs. S1–S10). Mammals and birds showed declines in all regions with all models and both RCPs. Plants showed consistent declines under RCP8.5, but projections were model- and region-dependent under RCP2.6. Amphibian and reptile diversity generally increased, although this was model- and region-dependent at the highest levels.

**Table 2:** Estimated maximum species richness (per km^2^) and the total area with the highest richness level for each region of Thailand for the present and projected for 2070, using three earth system models and two RCPs.

ESM and RCP	Maximum species richness	Number of 1 × 1 km grid cells with the highest species richness level						
		North-east	Central	East	North	South	Western	All regions
**Amphibian richness (> 24 spp./grid cell)**								
Current	31	870	4204	4086	381	3937	1375	14,853
CNRM-CM5 (RCP2.6)	31	2385	7903	4743	1967	12,233	4615	33,846
GFDL-CM3 (RCP2.6)	29	2394	3847	1134	1196	4009	2776	15,356
HadGEM2-ES (RCP2.6)	28	2272	3591	208	894	1925	338	9228
CNRM-CM5 (RCP8.5)	29	2297	2388	1693	1649	5027	2388	15,442
GFDL-CM3 (RCP8.5)	26	57	381	13	55	27	48	581
HadGEM2-ES (RCP8.5)	30	816	2651	693	70	1547	1707	7484
**Reptile richness (> 38 spp./grid cell)**								
Current	48	4	91	2220	0	0	300	2615
CNRM-CM5 (RCP2.6)	47	13	307	1445	40	0	1709	3514
GFDL-CM3 (RCP2.6)	44	3	13	144	0	0	1552	1712
HadGEM2-ES (RCP2.6)	44	446	318	133	4	0	41	942
CNRM-CM5 (RCP8.5)	47	77	151	70	11	0	3691	4000
GFDL-CM3 (RCP8.5)	45	170	435	235	1	0	799	1640
HadGEM2-ES (RCP8.5)	44	151	279	179	0	0	298	907
**Mammal richness (> 48 spp./grid cell)**								
Current	60	246	617	7	11	1	1221	2103
CNRM-CM5 (RCP2.6)	56	24	50	0	0	0	253	327
GFDL-CM3 (RCP2.6)	52	1	14	0	0	0	2	17
HadGEM2-ES (RCP2.6)	51	0	0	0	0	0	23	23
CNRM-CM5 (RCP8.5)	53	39	165	6	0	0	2	212
GFDL-CM3 (RCP8.5)	45	0	0	0	0	0	0	0
HadGEM2-ES (RCP8.5)	48	0	0	0	0	0	0	0
**Bird richness (> 352 spp./grid cell)**								
Current	440	1070	1134	269	3409	0	1232	7114
CNRM-CM5 (RCP2.6)	429	68	156	47	1290	0	352	1913
GFDL-CM3 (RCP2.6)	410	31	56	14	1010	0	636	1747
HadGEM2-ES (RCP2.6)	415	149	220	33	1230	0	161	1793
CNRM-CM5 (RCP8.5)	407	2	3	0	403	0	97	505
GFDL-CM3 (RCP8.5)	402	1	0	0	211	0	1	213
HadGEM2-ES (RCP8.5)	405	1	4	0	35	0	0	40
**Plant richness (> 368 spp./grid cell)**								
Current	458	1855	5863	893	8560	0	95	17,266
CNRM-CM5 (RCP2.6)	455	667	2608	447	5475	0	181	9378
GFDL-CM3 (RCP2.6)	450	273	2238	284	400	0	0	3195
HadGEM2-ES (RCP2.6)	446	2888	5769	696	10,571	0	92	20,016
CNRM-CM5 (RCP8.5)	450	379	2097	302	4514	0	16	7308
GFDL-CM3 (RCP8.5)	412	89	388	48	125	0	0	650
HadGEM2-ES (RCP8.5)	428	418	451	53	2828	0	7	3757

### Changes in the availability of suitable habitat for individual species

**Figure 4:**
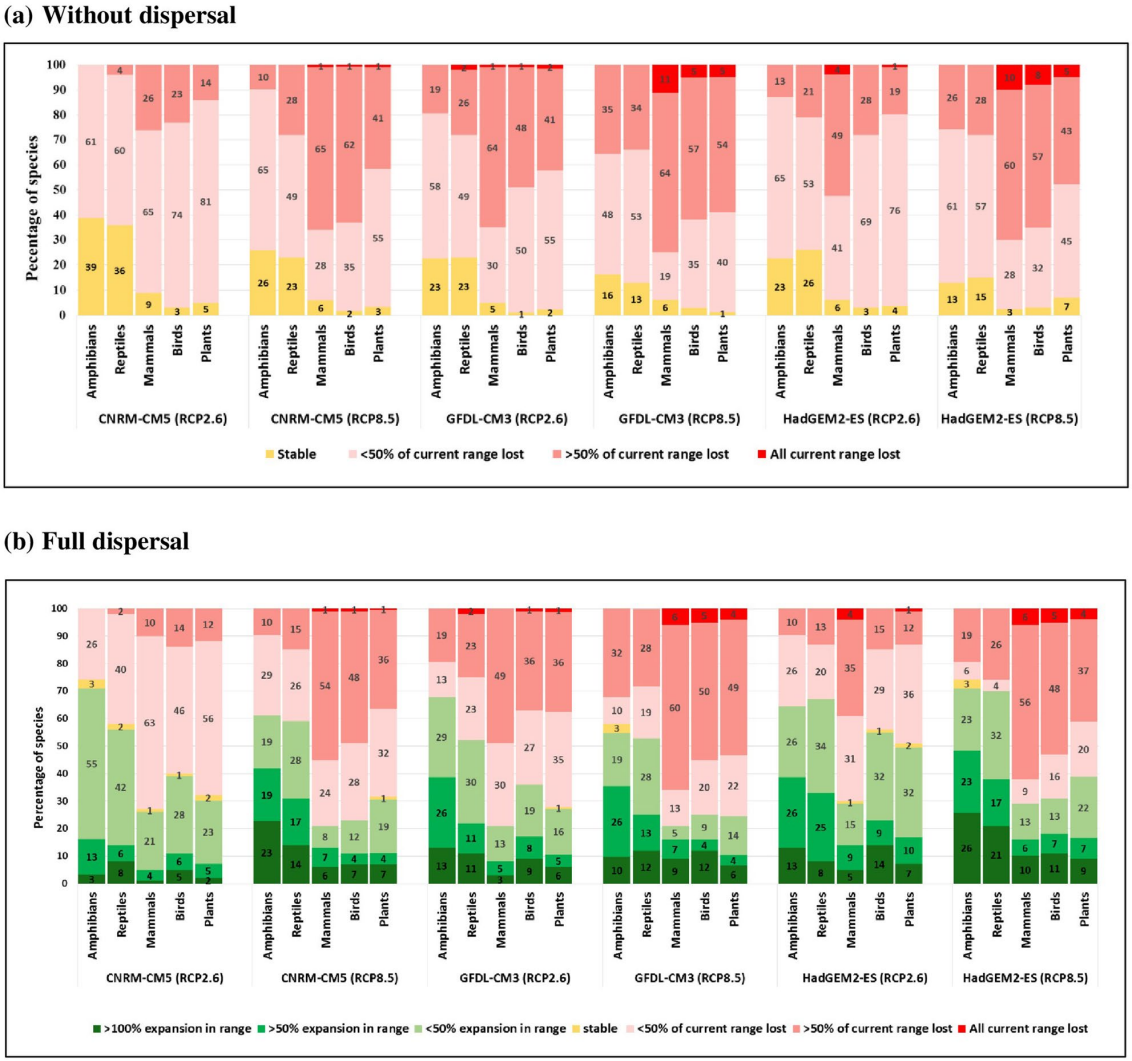
Projected changes in suitable habitat by 2070 (**a**) without and (**b**) with dispersal to newly suitable habitat for species in five taxa with three earth system models and two RCPs. Red colors represent projected habitat loss, yellow stability, and green expansion.

If expansions into newly suitable areas are not possible (i.e., no dispersal), under RCP2.6, 1–4% of mammal species are projected to lose all currently suitable habitat by 2070 and 26–64% to lose more than half (Fig. 4a). For plants, birds, and reptiles, 0–2% are projected to lose all suitable habitat and 14–41%, 23–48%, and 4–26%, respectively, to lose more than half. For amphibians, no species is projected to lose all suitable habitat, and 0–19% to lose more than half. Under RCP8.5, 1–11% of mammal species are projected to lose all currently suitable habitat and 60–65% to lose more than half. For birds, 1–8% are projected to lose all and 57–62% to lose more than half. For plants, 1–5% are projected to lose all and 41–54% to lose more than half. For reptiles and amphibians, no species are projected to lose all suitable habitat, and 28–34% and 10–35%, respectively, to lose more than half. If species can expand into newly suitable areas anywhere in Thailand (unlimited dispersal), many species show contractions and expansions in suitable habitat in different parts of their range. In amphibians and reptiles, the proportion of species projected to show net expansion is greater than those projected to show net contraction, while it is the other way round for mammals, birds, and plants (Fig. 4b). Under all scenarios, less than 25% of mammal species are projected to expand their ranges.

### Impacts on species in protected areas

The proportion of the habitat predicted as currently suitable for the modeled species that is included in PAs varies among taxa, ranging from 43% for mammals and 37% for birds to only 17% for amphibians and 22% for reptiles (Table 3). Overall, the extent of suitable habitat within PAs is projected to increase for the modeled amphibians and reptiles, but decrease for the mammals, birds, and plants, particularly under RCP8.5 (Table 3). Losses of suitable habitat for mammals under RCP8.5 are 26–38%. Even with full dispersal, mammal richness is projected to decline in 59–82% of PAs under RCP2.6 and 69–77% under RCP8.5 by 2070 (Supplementary Material Table S2). For birds and plants, impacts vary between models under RCP2.6 but declines dominate under RCP8.5. In contrast, most PAs have large projected increases in amphibian and reptile richness in all scenarios. The PAs are increasingly managed as part of protected area complexes (Fig. 1), and these vary in their vulnerability. Except for the three northernmost complexes, where habitat changes vary among models, and three complexes in the northern Central and Northeast regions for birds, all the complexes have projected declines in the extent of suitable habitat for modeled mammals, birds, and plants under RCP8.5 (Supplementary Material Table S3; Supplementary Material Figs. S6–S10). In contrast, all complexes have projected increases in suitable habitat for modeled amphibians and reptiles.

**Table 3:** The extent of suitable habitat for species within protected areas in Thailand currently and projected for 2070 for five taxa using three earth system models and two RCPs.

Taxon	Suitable habitat at present		ESMs	% change in suitable habitat under RCP2.6		% change in suitable habitat under RCP8.5	
	All (km^2^)	% protected		All	Protected	All	Protected
Amphibians	7,776,595	17	CNRM-CM5	20	22	33	55
			GFDL-CM3	22	33	13	52
			HadGEM2-ES	31	47	41	85
Reptiles	12,226,240	22	CNRM-CM5	13	11	21	25
			GFDL-CM3	9	7	14	25
			HadGEM2-ES	23	27	38	48
Mammals	7,585,451	43	CNRM-CM5	− 11	− 11	− 16	− 26
			GFDL-CM3	− 22	− 28	− 22	− 38
			HadGEM2-ES	− 12	− 20	− 19	− 33
Birds	84,834,145	26	CNRM-CM5	− 2	− 17	− 26	− 25
			GFDL-CM3	− 7	− 23	− 20	− 26
			HadGEM2-ES	19	6	− 5	− 14
Plants	81,136,326	37	CNRM-CM5	− 8	− 5	− 15	− 14
			GFDL-CM3	− 21	− 17	− 32	− 29
			HadGEM2-ES	6	4	− 5	− 8

### Projected changes in conservation status of species

Based on changes in the areas of suitable habitat, the numbers of modeled species in all threat categories will increase by 2070 under RCP2.6, while those rated as Least Concern more than halve, and the numbers of Endangered, Critically Endangered, and Extinct species will increase under RCP8.5 (Table 4; Supplementary Material Table S4). The overall percentage of modeled species assessed as threatened is projected to increase from 11% now to 35% under RCP2.6 and 54% under RCP8.5 (Supplementary Material Tables S5–S9).

**Table 4:** Current and projected 2070 conservation statuses of the modeled species.

Conservation status	Scenarios	The percentage of species for each taxon (%)				
		Amphibians	Reptiles	Mammals	Birds	Plants
Extinct (EX)	Current	–	–	–	–	–
	RCP2.6	–	–	–	–	–
	RCP8.5	–	–	1	1	1
Critically endangered (CR)	Current	–	–	–	–	–
	RCP2.6	–	–	1	2	2
	RCP8.5	3	8	36	25	16
Endangered (EN)	Current	–	2	3	1	4
	RCP2.6	3	9	30	17	14
	RCP8.5	10	13	20	22	23
Vulnerable (VU)	Current	–	2	14	10	7
	RCP2.6	13	13	30	16	19
	RCP8.5	13	8	11	11	12
Near threatened (NT)	Current	6	2	6	9	4
	RCP2.6	16	17	16	20	30
	RCP8.5	13	8	8	13	18
Least concern (LC)	Current	94	94	78	80	85
	RCP2.6	68	60	23	44	36
	RCP8.5	61	64	24	29	31
Total threatened species	Current	–	4	16	11	11
	RCP2.6	16	23	61	35	35
	RCP8.5	26	28	69	59	52

Eleven modeled species are projected to become extinct in Thailand by 2070 under RCP8.5. These include one primate with a northern distribution, *Trachypithecus crepusculus* (formerly considered a subspecies of *T. phayrei*), six bird species, and four plant species. The birds include the Chinese crested tern, *Thalasseus bernsteini,* a migratory seabird for which our data is incomplete, the ferruginous partridge, *Caloperdix oculeus*, found in SW Thailand and countries to the south, the chestnut-tailed minla, *Actinodura strigula*, found at high altitudes in NW Thailand, the Nepal house-martin, *Delichon nipalense*, a rare winter visitor to the north, the black-throated laughingthrush (*Ianthocincla chinensis*), widespread in the northern half of Thailand, and the ratchet-tailed treepie (*Temnurus temnurus*), found locally in southwestern Thailand. The plant species have varied but generally narrow current distributions in Thailand. They are *Acacia leucophloea*, a tree of dry forests and savanna, two species of montane epiphytic shrubs in the Ericaceae, *Agapetes loranthiflora* and *Diplycosia* cf. *heterophylla*, and a member of the Zingiberaceae, *Scaphochlamys obcordata*, from forest in southernmost Thailand. However, projected responses of individual species are varied and complex (Supplementary Material Tables S5–S9), and, in some cases, may reflect data limitations more than climate-change vulnerability.

## Discussion

Although the details are complex, the general patterns that emerge from the results are clear. Most modeled species of mammals, birds, and plants are projected to suffer large declines by 2070, although projected responses are model-dependent for birds and plants under RCP2.6. By contrast, more modeled amphibian and reptile species are projected to experience an increase in suitable habitat than a decline and the species richness of these taxa is projected to increase in most areas. Projected impacts on species within PAs are generally similar. The overall impact on the conservation status of the modeled species is to push many of them into higher threat categories, so that by 2070 under RCP8.5, 54% of all modeled species will be threatened and 11 extinct in Thailand. However, before the implications of these results are discussed further, it is important to consider the limitations of the modeling approach used and their possible consequences.

The ability of a species to maintain a viable population at a site is determined by abiotic conditions, including climate, plus biotic interactions and dispersal limitation^21^. The use of correlative species distribution models (SDMs) to model climate change impacts on species distributions rests on the assumption that the location data used to model the current distributions represents the entire climate space in which each species can live as part of a community. If the climate space currently occupied has been truncated in some way, then the models will underestimate potential future distribution and thus potentially overestimate the impacts of climate change^22^. Actual niche truncation can arise from both natural and anthropogenic factors, while apparent truncation may result from data availability. Non-climatic natural factors, including edaphic specialization, biotic interactions, and dispersal limitation, may prevent species from occupying their entire potential climate niche^23–25^. More generally, most tropical lowland species may have truncated upper thermal limits because warmer climates with similar rainfall do not currently exist^26,27^. Anthropogenic niche truncation through habitat loss and direct exploitation is probably universal in areas where people live^22,28^.

In Thailand, the clearance of most lowland forests, particularly in the hotter, drier, Central and East regions, has biased recent location records for forest-dependent species to cooler, wetter mountainous areas, and hunting of most large vertebrates has confined them to a subset of well-protected PAs. In addition to these actual truncations of species’ niches, the bias in our location dataset to existing PAs, particularly for mammals, and the exclusion of areas outside Thailand (because of sparse data from neighboring regions of Myanmar and Laos, which have the longest borders with Thailand), are artificial cut-offs which may influence model projections. While the eleven species projected to become extinct by 2070 under RCP8.5 are all plausible, given the magnitude of the bioclimatic changes, some of the projected declines for mammals, birds, and plants (Supplementary Material Tables S5–S9) may reflect lowland niche truncation, leading to underestimates of their potential future distributions and thus overestimates of the impacts of climate change. Without an independent dataset, it is not possible to check this. The problems are different for the amphibian and reptile datasets. These are both dominated by widespread open-country and forest edge species—because forest-dependent species need special techniques for effective surveys—and such species are pre-adapted to the warmer and sometimes drier conditions projected for PAs by 2070. The projected increases in richness of these taxa in most areas probably masks a real threat to forest-dependent species.

Predictions for the climate change responses of individual species can be very different depending on assumptions about dispersal limitations. Estimates of maximum routine dispersal distances for plant species in Southeast Asia range from < 10 m to > 10 km^29^ and the range for vertebrates is certainly larger. Ideally, impact projections would be made for individual species by combining dispersal ability with the availability and spatial configuration of suitable habitat, but for the great majority of species in Thailand the dispersal component would just be a guess. We therefore used ‘no dispersal’ and ‘unlimited dispersal’ (i.e., all suitable niche space in Thailand is occupied) to bracket the range of possibilities.

Additional problems arise when SDMs for multiple species are stacked to predict local species richness, as was done here. Although individual SDMs capture the combined effects of both the abiotic and biotic environments on current distributions, it is unreasonable to assume that biotic effects will not change under future climates. Various alternative statistical approaches have been proposed, but they make additional demands on data quality and do not result in consistently improved predictions^21^. Moreover, stacked SDMs are easier to apply and understand, and, although the absolute richness values should be treated with caution, the spatial patterns are likely to be robust.

At least two additional factors need to be taken into account. Firstly, the vegetation, and thus the available habitat for animals and smaller plants, over much of Thailand is strongly influenced by the fire regime^13,30,31^, which was not included in our models. Climate change could potentially increase or decrease fire frequency and intensity leading, respectively, to degradation of fire-sensitive forests or to canopy closure in fire-dependent open forests. Secondly, our models also do not include the direct ecophysiological effects of rising carbon dioxide levels, which will likely favor C3 trees over C4 grasses, leading to increased woody encroachment. A recent study simulated the effects of climate change on vegetation in South Asia, with and without increasing CO_2_^32^. Simulations with increasing CO_2_ resulted in transitions from savanna into forest and deciduous into evergreen forest which did not occur without increasing CO_2_. We also could not model the impacts of rare climatic extremes, such as floods, droughts, and extreme temperature maxima, or the secondary effects of primary impacts on important predator and prey species, and on essential mutualists.

We have previously taken a purely physical-climate approach to projecting bioclimatic conditions in Thailand’s PAs in 2070, using the same three earth system models and the same two RCPs^7^. This approach has the advantages of allowing a uniform coverage of the whole country and avoiding the accumulation of model errors that affects projections using species distribution data as well as physical climate data. On the other hand, bioclimatic types are not themselves targets for conservation, and converting projections for changes in bioclimate into projections of conservation impacts is still prone to errors and uncertainties. Taken together, however, the projections of pervasive changes in bioclimates and massive impacts on forest-dependent species suggest that Thailand needs to consider climate change in protected area planning and management.

## Management recommendations

Thailand currently has a large, fairly representative, and increasingly well-run protected area system. The issue of fragmentation is being addressed by the establishment of PAs complexes (Fig. 1), within which some PAs have existing connections, which allow movements of species, and the options for connecting additional areas, by corridors or steppingstones, are being investigated^18^. South to north and low to high altitude connectivity will be particularly important in allowing species to move in response to climate change and these should be prioritized when possible. However, many species will probably not be able to track the rapid climate change projected for the next 50 years^25,33^. Mitigation for these impacts could include in situ support and assisted migration to other PAs.

In view of the certainty of future warming but the uncertainties regarding biological responses, one management priority needs to be long-term monitoring of species and communities in order to detect early signals of climate change impacts. Currently available information on trends in the abundances and distributions of species in Southeast Asia is largely anecdotal^12^. Altitudinal transects are a useful tool for monitoring since the steep gradient in temperature with elevation (c. 0.6 °C/100 m) amplifies the expected response to warming. Ideally, permanent transects would be established in PAs from south to north Thailand. Impact signals are expected to be detectable first in highly mobile species, like most birds^34^, in species with rapid life cycles, like insects^35^, and in the regeneration success of forest plants^36^. Although collaborations with universities and volunteer citizen scientists are useful, consistent monitoring over multiple decades will need leadership and supervision from the Department of National Parks, Wildlife and Plant Conservation (DNP).

## Methods

### Study area

The study area covers the total land area of Thailand. Where it is useful, we divided Thailand into six regions (Fig. 2a), the names and boundaries of which are widely used, although they have no official administrative status. We focused on the elements of Thailand’s protected area system that were concerned principally with the in-situ conservation of biodiversity: existing and proposed National Parks, Wildlife Sanctuaries, Non-hunting Areas, and Forest Parks, covering 111, 201 km^2^ or 21.7% of the country’s land area^37^ (Fig. 1).

### Environmental data

A set of environmental variables that were expected to be directly or indirectly related to species distributions in Thailand was used to model suitable habitat in the present and future (Supplementary Material Table S1). These variables were chosen to encompass ecologically relevant variables and enable consistent comparison between species, regardless of species-specific preferences. GIS layers for the whole of the study area were compiled using a variety of data sources at 1-km^2^ resolution. For variables originally at higher than 1-km resolutions, we used the plus function in ArcMap to combine them with a mask of the study area to use the mask dimensions for all cells.

The physical variables, altitude, slope, aspect, and soil pH are widely used in species distribution modeling. Slope and aspect have biologically significant impacts on both temperature and rainfall at these latitudes^8^ and are particularly important at the poleward margins of species ranges where species may be confined to one aspect. Slope also affects soil maturity and depth. Soil pH is a consistently measured soil variable that broadly correlates with fertility in tropical soils^8^. Additional soil variables, particularly soil phosphorus, have been shown to be important filters of plant species distributions in the tropics^38^, but they are not available for Thailand with a useful accuracy and spatial resolution. Altitudes were downloaded from the CGIAR-Consortium for Spatial Information, CGIAR-CSI version 4.1. Slope and aspect were generated by using surface tools in ArcGIS. Soil pH was extracted from ISRIC-World Soil Information version 2.0.

Unlike the temperate zone, where tolerances of winter cold and requirements for summer warmth dominate plant and animal distributions, our understanding of how tropical climates filter species distributions is still weak^38,39^. In Thailand, as in most of the tropics, there are two major climatic gradients which correlate with changes in species composition: a rainfall gradient in the lowlands, along which total rainfall declines and the length of the dry season increases, and a gradient of steadily declining temperature with elevation^7^. There is no simple relationship between elevation, and thus temperature, and rainfall. An additional complication is that temperature seasonality may be significant in northern Thailand (north of c.18° N), where cooler winters reduce dry-season water stress and extreme low temperatures at high altitudes may exceed physiological tolerances. We therefore chose 8 bioclimatic variables (Supplementary Material Table S1) related to precipitation and temperature, and their seasonality, all of which have previously been used in species distribution modelling in this region^9,40^. These are available at a resolution of 30 arc sec (approximately 1 km at the equator) from WorldClim ver. 1.4 based on averages of 1970–1990. These variables are available from the same source (and downscaled using the same methods) for the future climate projections.

Vegetation structure is an additional major influence on plant and animal distributions in the tropics, both in intact natural vegetation^38,39^ and when the original vegetation has been degraded or cleared^8^. Vegetation structure was represented through the inclusion of two continuous variables, percentage forest cover and tree density, as most of the modelled species are known to be sensitive to both the presence of forest and the degree of intactness of the tree cover^9^. Mean tree density per km^2^ was extracted from Crowther et al.^41^ version 2 and percentage coverage of forest per km^2^ was extracted from the European Space Agency (ESA) GlobCover Version 2.3.

Note that the mechanistic basis of the correlations between all these variables and the current distributions of tropical plants and animals are rarely known. Temperature has a direct physiological impact on all organisms, and water supply may be seasonally limiting for plants and some amphibians, but indirect links through biotic interactions are expected to be more important in the tropics, including pest pressure on plants^38^ and food supply for animals^39^. Competition is probably also important in shaping local species assemblies. For future projections, we assumed that temperature and precipitation were changing, and that other variables (topography, soil, and vegetation) were stable, so our analysis represents the impacts of climate alone. For 2070, we used the same variables projected by three CMIP5 Earth System Models, CNRM-CM5, GFDL-CM3 and HadGEM2-ES, which have been previously used in Southeast Asia^9,42^ and in Thailand^7^. We used two Representative Concentration Pathways, RCP2.6 and RCP8.5, representing low and high greenhouse-gas concentration scenarios, respectively, and thus the potential range of radiative forcing by the end of the century^43^. RCP2.6 is consistent with meeting the Paris Agreement’s 2 °C global warming target.

### Species occurrence data

Many locality records for vertebrates were supplied by the Department of National Parks, Wildlife and Plant Conservation (DNP). Trained DNP staff walked along trails throughout the protected areas in Thailand during 2017–2018. They recorded 271,695 locations for 70 mammal species, 18 locations for 3 amphibian species, 318 locations for 18 reptile species, and 43,057 locations for 65 bird species^44^. We supplemented this with data downloaded from the Global Biodiversity Information Facility (GBIF, https://www.gbif.org/) for 1960–2019 for amphibians (2063 localities for 86 species)^45^, reptiles (1722 localities from 196 species)^46^, mammals (2508 localities from 191 species)^47^, and birds (1,559,222 localities from 884 species)^48^. More than 95% of the bird records from GBIF were identified as coming from eBird^49^, which is popular among birders in Thailand. For plants, we used occurrence data from the DNP’s forest resource inventory project from 221 plots, including 24,605 localities for 363 species, the DNP’s Forest Herbarium, including 227 localities for 141 species, and locations for 12 rare and endangered forest species collected from all over Thailand. We also downloaded data from the Botanical Information and Ecology Network (BIEN, https://bien.nceas.ucsb.edu/bien/), including 7209 localities for 1422 species.

We removed suspect records (coordinate issues, name problems, etc.), duplicates from the same locality (i.e., more than one individual of the same species recorded in a cell), and species with < 10 localities. High levels of duplication reduced the sizes of the datasets from GBIF and BIEN, so only 687 localities for 29 amphibian species, 1409 localities for 36 reptile species, 2050 localities for 52 mammal species, 282,026 localities for 697 bird species, and 1978 localities for 223 plant species were added to the data from the DNP. The total number of records used for analysis was therefore 705 localities for 31 amphibian species, 1697 localities for 53 reptile species, 243,423 localities for 80 mammal species, 325,008 localities for 702 bird species, and 34,440 localities for 591 plant species.

### Species distribution modeling

Species distribution models (SDMs) assume the relationship between a pattern of interest (e.g., species abundance or species occurrence) and a set of factors assumed to control it can be quantified. Here we used Maxent, based on the maximum-entropy approach, to predict suitable habitats for each species, then summed them to look at changing diversity patterns. Maxent parameters, unless otherwise indicated, were set to the defaults, as these are broadly optimized across species. The same background area—the total land area of Thailand—was used for all species. For each species with more than 10 unique occurrence records, three replicates were run and the average of the three models used for further analysis. The Maxent output was then converted into binary presence-absence maps using the 10% cumulative logistic threshold, because it has previously been found to be the most accurate and conservative threshold for delineating suitable from unsuitable areas^9^. The predictive performance of Maxent was evaluated using the continuous Boyce Index, a presence-only and threshold-independent metric which measures how much the predictions of the SDMs differ from a random distribution of occurrences along the prediction gradient^50^. We considered values > 0.5 as adequate, but since only five SDMs out of the 1457 generated in this study had values lower than this (0.3–0.5), we retained all the models.

### Assessment of climate change impacts

The estimated current distribution for each species from Maxent was used as the baseline for comparison with projected distributions of suitable habitat for these species by 2070, under the two emission scenarios and three ESMs, and with and without unlimited dispersal into newly available habitat. We then assessed the impacts of climate change, both on the spatial distribution of individual species and on the pattern of species richness. To generate a species richness map, the binary habitat suitability maps for all species were stacked to produce a consolidated map, which showed the number of species for each 1 km grid cell, and then classified them into five classes (lowest, low, moderate, high, and highest), using the mean ± standard deviation as a break class^40^.

Current and future maps were then compared for each species to calculate the change in species richness, and contingency tables were generated containing the numbers of cells (each of 1 km^2^) in each richness class. Suitable habitat areas were calculated for the current climate and projected for the future climate. For each species we estimated gained habitat as the areas that will become suitable for a species in future under that scenario, lost habitat as the areas currently predicted as suitable now but projected to become unsuitable under future climatic change, and stable habitat as the areas predicted as suitable now which will remain suitable into the future.

We then assessed the vulnerability of each species by estimating the projected change in its range over the next 50 years and using a criteria-based approach, which combined the mean of the suitable habitat area (interpreted as equivalent to extent of occurrence) in the three models and a simplified version of the IUCN Red List criteria^51^. For 2070, we modified criterion A3(c) as follows; Extinct (Ex) species are projected to lose 100% of suitable habitat by 2070, Critically Endangered (CR) species are projected to lose over 80%, Endangered (EN) species are projected to lose 50–80%; Vulnerable (VU) species are projected to lose 30–50%, Near Threatened (NT) species are projected to lose < 30%, and Least Concern (LC) show no projected loss. For the current status, we used only the modeled extent of suitable habitat: CR < 100 km^2^ of suitable habitat, EN < 5000 km^2^, VU < 20,000 km^2^, NT < 30,000 km^2^, and LC > 30,000 km^2^. In practice, other, non-climatic, factors will also influence extinction risk and may interact in unpredictable ways with changes in climate, so these assessments for individual species based only on climate change projections should not be used in isolation in conservation planning.

The 386 existing and 30 proposed PAs were overlaid on the predicted current and projected future species distributions to assess the impact of climate change on their ability to protect the modeled species. First, we assessed the difference between current and future predicted richness. We then assessed the ability of the protected area system as whole to support the conservation of species effectively, by calculating the change in the percentage of suitable habitat and identified which PAs complexes are likely to be most vulnerable.

## Supplementary Information

Supplementary Information.

## Data Availability

All data analyzed in this study is presented in Tables in the text and Supplementary Materials or was obtained from publicly accessible databases cited in the text, except for the protected area shapefiles, which were provided by the Department of National Parks, Wildlife and Plant Conservation, and the vegetation type shapefiles, which were provided by the Royal Forest Department, to whom requests for data should be sent.
