# Supplementary material for: Vulnerability to climate change of species in protected areas in Thailand
Source: Sci Rep. 2022 Apr 5;12:5705. doi: 10.1038/s41598-022-09767-9 (PMC8983663; doi:10.1038/s41598-022-09767-9)
Supplement: Supplementary file 1 — Supplementary Information. [file 41598_2022_9767_MOESM1_ESM.pdf]

**Journal name**: Scientific Reports

# Vulnerability to climate change of species in protected areas in Thailand

**Nirunrut Pomoim[1,2], Alice C. Hughes[1,3], Yongyut Trisurat[4], Richard T. Corlett[1,3]**

[1] Center for Integrative Conservation, Xishuangbanna Tropical Botanical Garden, Chinese Academy of Sciences, Menglun, Yunnan, China. nirunrut@xtbg.ac.cn (NP); ach_conservation2@hotmail.com (ACH); corlett@xtbg.org.cn (RTC)
[2] University of Chinese Academy of Sciences, Beijing, China
[3] Center of Conservation Biology, Core Botanical Gardens, Xishuangbanna Tropical Botanical Garden, Chinese Academy of Sciences, Menglun, Yunnan, China.
[4] Faculty of Forestry, Kasetsart University, Bangkok, Thailand. fforyyt@ku.ac.th
Correspondence: corlett@xtbg.org.cn

**Table S1** Data layers used to develop the models.

| Environment variables | Source |
|---|---|
| **Climatic variables** | www.worldclim.org |
| 1. annual mean temperature | |
| 2. Mean temperature of wettest quarter | |
| 3. Mean temperature of driest quarter | |
| 4. Mean temperature of warmest quarter | |
| 5. Mean temperature of coldest quarter | |
| 6. Annual precipitation | |
| 7. precipitation of wettest quarter | |
| 8. precipitation of driest quarter | |
| 9. precipitation of warmest quarter | |
| 10. precipitation of coldest quarter | |
| **Physical variables** | |
| 11. Altitude | http://www.cgiar-csi.org/data/srtm-90m-digital-elevation-database-v4-1 |
| 12. Slope | extracted by using surface tools in ArcGIS |
| 13. Aspect | extracted by using surface tools in ArcGIS |
| 14. Soil pH | http://soilgrids.org/ |
| **Biological variables** | |
| 15. Vegetation cover | http://due.esrin.esa.int/page_globcover.php |
| 16. Tree density | https://figshare.com/articles/Global_map_of_tree_density/3179986 |

**Table S2** Numbers of protected areas showing changes in species richness by 2070 under climate change, using three earth system models and two RCPs. Eighteen very small protected areas were excluded because of the spatial resolution of the data, leaving a total of 398.

| Taxon | Change | RCP2.6 | | | RCP8.5 | | |
|---|---|---|---|---|---|---|---|
| | | CNRM-CM5 | GFDL-CM3 | HadGEM2-ES | CNRM-CM5 | GFDL-CM3 | HadGEM2-ES |
| Amphibians | Loss | 7 | 44 | 16 | 22 | 81 | 28 |
| | Stable | 41 | 35 | 13 | 10 | 14 | 18 |
| | Gain | 350 | 319 | 369 | 366 | 303 | 352 |
| Reptiles | Loss | 14 | 126 | 53 | 39 | 80 | 53 |
| | Stable | 40 | 46 | 19 | 19 | 13 | 15 |
| | Gain | 344 | 226 | 326 | 340 | 305 | 330 |
| Mammals | Loss | 270 | 328 | 237 | 289 | 305 | 274 |
| | Stable | 46 | 34 | 22 | 27 | 23 | 13 |
| | Gain | 82 | 36 | 139 | 82 | 70 | 111 |
| Birds | Loss | 233 | 235 | 142 | 271 | 306 | 226 |
| | Stable | 8 | 8 | 2 | 1 | 2 | 0 |
| | Gain | 157 | 155 | 254 | 126 | 90 | 172 |
| Plants | Loss | 305 | 370 | 167 | 330 | 382 | 257 |
| | Stable | 10 | 3 | 5 | 4 | 1 | 4 |
| | Gain | 83 | 25 | 226 | 64 | 15 | 137 |

**Table S3** Changes in the total extent of suitable habitat for taxa within protected areas complexes in Thailand using three earth system models and two RCPs.

| Protected area complex | Taxon | suitable habitat at present ($km^2$) | %suitable habitat change under RCP2.6 | | | %suitable habitat change under RCP8.5 | | |
|---|---|---|---|---|---|---|---|---|
| | | | CNRM-CM5 | GFDL-CM3 | HadGEM2-ES | CNRM-CM5 | GFDL-CM3 | HadGEM2-ES |
| Lumnampai-Salawin | amphibians | 137,863 | 0.3 | 0.3 | 0.4 | 0.5 | 0.5 | 0.8 |
| | reptiles | 315,124 | 0.2 | 0.1 | 0.5 | 0.9 | 0.8 | 1.2 |
| | mammals | 259,690 | 11.9 | -1.9 | -6.9 | 4.1 | -8.6 | -16.0 |
| | birds | 2,835,953 | 1.5 | -27.2 | -6.2 | -17.1 | -28.2 | -37.3 |
| | plants | 4,495,421 | 1.4 | -11.7 | 0.1 | -5.0 | -21.9 | -12.3 |
| Srilanna-Khuntan | amphibians | 67,806 | 0.2 | 0.2 | 0.6 | 0.8 | 0.8 | 1.5 |
| | reptiles | 148,393 | 0.2 | -0.1 | 0.4 | 0.5 | 0.3 | 0.9 |
| | mammals | 141,074 | 19.4 | -22.7 | -3.1 | -10.2 | -17.5 | 0.6 |
| | birds | 2,205,088 | -2.8 | -34.8 | 9.1 | -14.8 | -41.0 | -10.2 |
| | plants | 2,968,661 | 0.3 | -17.4 | 10.9 | -11.0 | -31.0 | -3.7 |
| DoiPhuka-Maeyom | amphibians | 34,552 | 0.2 | 0.1 | 0.9 | 0.7 | 0.8 | 2.3 |
| | reptiles | 74,827 | 0.2 | -0.2 | 0.8 | 0.5 | 0.3 | 1.5 |
| | mammals | 34,588 | -3.8 | -21.9 | 37.6 | -13.7 | -16.9 | 53.8 |
| | birds | 914,226 | -21.1 | -29.3 | 72.8 | -26.1 | -47.5 | 59.3 |
| | plants | 1,575,849 | -12.3 | -22.1 | 40.2 | -12.8 | -37.1 | 27.2 |
| Maeping-Omgoi | amphibians | 124,146 | 0.2 | 0.3 | 0.4 | 0.5 | 0.3 | 0.5 |
| | reptiles | 270,650 | 0.1 | -0.1 | 0.1 | 0.1 | 0.1 | 0.2 |
| | mammals | 239,134 | -15.9 | -37.0 | -26.5 | -23.5 | -28.1 | -30.1 |
| | birds | 2,282,807 | -16.7 | -34.1 | -23.1 | -30.8 | -18.8 | -37.6 |
| | plant | 2,862,558 | -2.5 | -25.7 | -10.5 | -16.0 | -32.2 | -21.2 |
| Phumeing-Phuthong | amphibians | 96,130 | 0.2 | 0.4 | 0.8 | 0.7 | 0.8 | 1.3 |
| | reptiles | 235,634 | 0.1 | 0.0 | 0.5 | 0.3 | 0.3 | 0.7 |
| | mammals | 294,843 | -18.3 | -38.0 | -21.1 | -35.0 | -32.0 | -34.2 |
| | birds | 1,783,458 | -33.1 | -30.3 | 44.2 | -40.6 | -20.3 | 31.9 |
| | plants | 3,297,320 | -9.8 | -21.0 | 14.9 | -14.9 | -33.4 | -3.5 |
| Phukheio-Namnow | amphibians | 96,309 | 0.1 | 0.3 | 0.6 | 0.6 | 0.7 | 1.0 |
| | reptiles | 216,293 | 0.0 | 0.0 | 0.4 | 0.3 | 0.4 | 0.6 |
| | mammals | 268,053 | -26.7 | -32.8 | -29.2 | -24.7 | -29.8 | -42.4 |
| | birds | 2,002,089 | -63.0 | -34.5 | 22.2 | -48.5 | -20.2 | -2.0 |
| | plant | 3,136,948 | -13.6 | -17.6 | 5.8 | -15.8 | -26.3 | -12.3 |
| Phuphan | amphibians | 20,604 | 0.5 | 0.9 | 1.4 | 1.2 | 1.0 | 1.9 |
| | reptiles | 36,807 | 0.3 | 0.6 | 1.0 | 0.8 | 0.9 | 2 |

| | | | | | | | | |
|---|---|---|---|---|---|---|---|---|
| | mammals | 29,142 | -12.5 | -27.8 | 17.9 | -6.2 | 8.5 | 19.5 |
| | birds | 48,721 | 9.8 | 30.3 | 93.3 | 38.0 | 7.4 | 187.7 |
| | plants | 408,916 | -10.1 | -16.1 | 28.6 | -20.2 | -22.4 | 40.2 |
| Phanomdongrak-Phataem | amphibians | 30,317 | 0.5 | 0.8 | 0.9 | 1.0 | 0.8 | 1.3 |
| | reptiles | 55,435 | 0.0 | 0.3 | 0.5 | 0.5 | 0.5 | 0.9 |
| | mammals | 87,271 | -36.6 | -53.7 | -45.2 | -42.0 | -43.9 | -39.5 |
| | birds | 130,296 | -58.4 | 48.9 | 50.2 | 3.1 | 36.9 | 84.4 |
| | plants | 429,125 | -18.4 | -13.0 | 4.8 | -13.2 | -22.6 | 13.0 |
| DongPhayayen-Khaoyai | amphibians | 107,724 | 0.2 | 0.3 | 0.4 | 0.5 | 0.4 | 0.5 |
| | reptiles | 246,881 | 0.0 | 0.1 | 0.2 | 0.1 | 0.1 | 0.2 |
| | mammals | 263,138 | -23.3 | -32.2 | -33.2 | -30.8 | -37.5 | -46.3 |
| | birds | 1,997,809 | -27.9 | -12.8 | -8.9 | -26.7 | -25.2 | -20.9 |
| | plants | 2,459,240 | -11.5 | -22.1 | -5.3 | -19.8 | -30.5 | -21.5 |
| Eastern | amphibians | 49,054 | 0.2 | 0.1 | 0.3 | 0.3 | 0.1 | 0.3 |
| | reptiles | 89,934 | 0.1 | 0.1 | 0.2 | 0.1 | 0.1 | 0.2 |
| | mammals | 63,120 | -12.1 | -23.5 | -11.0 | -17.2 | -28.6 | -23.6 |
| | birds | 289,601 | 25.3 | 16.0 | 69.9 | 10.5 | -0.6 | 39.1 |
| | plants | 547,624 | -8.0 | -24.2 | -1.8 | -16.7 | -30.8 | -7.5 |
| Western | amphibians | 180,336 | 0.3 | 0.6 | 0.6 | 0.8 | 0.9 | 1.3 |
| | reptiles | 350,761 | 0.1 | 0.2 | 0.5 | 0.4 | 0.6 | 0.9 |
| | mammals | 777,625 | -10.0 | -24.9 | -27.8 | -38.5 | -55.5 | -43.0 |
| | birds | 2,624,819 | -32.3 | -39.6 | -13.6 | -48.0 | -39.8 | -30.1 |
| | plants | 4,268,797 | -0.2 | -9.8 | 0.0 | -13.9 | -23.4 | -3.4 |
| Khaengkrachan | amphibians | 69,853 | 0.3 | 0.5 | 0.4 | 0.6 | 0.7 | 0.8 |
| | reptiles | 181,525 | 0.1 | 0.1 | 0.1 | 0.1 | 0.2 | 0.2 |
| | mammals | 250,067 | -8.8 | -26.9 | -22.7 | -28.4 | -42.4 | -45.6 |
| | birds | 1,608,447 | -8.5 | -19.4 | -16.1 | -25.6 | -28.1 | -32.4 |
| | plants | 1,576,975 | -2.7 | -13.2 | -5.8 | -16.7 | -26.9 | -23.2 |
| Chumporn | amphibians | 34,390 | 0.2 | 0.3 | 0.4 | 0.5 | 0.4 | 0.6 |
| | reptiles | 52,096 | 0.2 | 0.2 | 0.1 | 0.3 | 0.2 | 0.2 |
| | mammals | 69,091 | -4 | -23 | 3 | -14 | -38 | -34 |
| | birds | 402,770 | 18.2 | 27.4 | 29.2 | 36.1 | 16.3 | -2.6 |
| | plants | 255,654 | -9.4 | -19.6 | 5.1 | -10.5 | -32.0 | -10.4 |
| Klongsaeng-Khaosok | amphibians | 87,333 | 0.2 | 0.2 | 0.3 | 0.4 | 0.2 | 0.4 |
| | Reptiles | 122,456 | 0.1 | 0.1 | 0.1 | 0.2 | 0.0 | 0.0 |
| | mammals | 200,738 | -10.8 | -30.5 | -8.3 | -24.0 | -53.3 | -43.0 |
| | birds | 1,202,194 | 6.4 | 9.2 | 14.3 | 7.2 | -10.6 | -19.4 |
| | plants | 592,319 | -11.9 | -19.5 | 14.4 | -11.3 | -38.8 | -15.1 |
| KhaoLuang | amphibians | 33,156 | 0.1 | 0.2 | 0.2 | 0.3 | 0.3 | 0.4 |
| | reptiles | 42,988 | 0.1 | 0.0 | 0.2 | 0.2 | 0.1 | 0.3 |

| | | | | | | | | |
|---|---|---|---|---|---|---|---|---|
| | mammals | 59,367 | -8.4 | -22.4 | 7.4 | -6.8 | -40.0 | -2.7 |
| | birds | 389,567 | -6.2 | -10.4 | 12.7 | -5.7 | -25.9 | -9.5 |
| | plant | 317,128 | -2.8 | -12.2 | 9.1 | -11.1 | -33.0 | -7.6 |
| Khaobantad | amphibians | 61,649 | 0.1 | 0.2 | 0.1 | 0.2 | 0.0 | 0.2 |
| | reptiles | 89,864 | 0.1 | 0.0 | 0.0 | 0.1 | -0.1 | -0.1 |
| | mammals | 97,721 | -9.5 | -31.1 | -15.0 | -24.7 | -51.0 | -38.7 |
| | birds | 666,510 | 3.2 | -13.5 | -10.7 | -16.3 | -26.1 | -37.4 |
| | plants | 441,839 | -5.4 | -19.8 | -5.9 | -20.2 | -39.5 | -26.0 |
| Hala-Bala | amphibians | 38,921 | 0.1 | 0.2 | 0.3 | 0.3 | 0.1 | 0.4 |
| | reptiles | 49,237 | 0.1 | 0.1 | 0.2 | 0.2 | 0.0 | 0.2 |
| | mammals | 36,633 | -15.6 | -19.8 | 14.1 | -13.3 | -36.3 | 9.4 |
| | birds | 253,331 | -5.0 | 7.6 | 43.6 | -2.0 | -19.8 | 28.4 |
| | plants | 252,698 | -13.0 | -17.7 | 15.1 | -22.0 | -39.8 | 5.0 |
| Andaman Island | amphibians | 26,465 | 0.1 | 0.0 | 0.1 | 0.1 | -0.1 | 0.0 |
| | reptiles | 37,115 | 0.1 | 0.0 | -0.1 | 0.0 | -0.2 | -0.2 |
| | mammals | 34,588 | -14.0 | -37.6 | -24.1 | -34.8 | -53.0 | -45.2 |
| | birds | 321,309 | -3 | -13 | -7 | -25 | -27 | -42 |
| | plant | 128,577 | -3.3 | -22.7 | -1.6 | -20.7 | -42.3 | -27.6 |
| Angthong Island | amphibians | 3,836 | 0.1 | 0.1 | 0.2 | 0.2 | 0.1 | 0.2 |
| | reptiles | 5,373 | 0.1 | 0.0 | 0.0 | 0.1 | 0.0 | 0.1 |
| | mammals | 5,353 | -9.4 | -26.6 | -20.2 | -16.1 | -51.2 | -34.4 |
| | birds | 53,180 | -12 | -22 | -23 | -16 | -34 | -44 |
| | plants | 25,399 | -9.5 | -16.1 | -8.8 | -18.0 | -39.5 | -29.7 |
| Eastern Island | amphibians | 2,453 | 0.2 | 0.4 | 0.4 | 0.5 | 0.3 | 0.5 |
| | reptiles | 3,648 | 0.1 | 0.3 | 0.1 | 0.3 | 0.0 | 0.1 |
| | mammals | 2,723 | -22.5 | -20.2 | -31.9 | -20.6 | -33.0 | -34.9 |
| | birds | 20,215 | 0 | 40 | -19 | -10 | -5 | -19 |
| | plants | 11,941 | -9.2 | -16.8 | -32.7 | -14.2 | -29.3 | -16.7 |

**Table S4** Estimated conservation status of all modeled species currently and in 2070, based on the modeled extent of suitable habitat, averaged across three earth system models.

| Status | number of species | | | percentage of species | | |
|---|---|---|---|---|---|---|
| | Current | RCP2.6 | RCP8.5 | Current | RCP2.6 | RCP8.5 |
| Extinct (EX) | 0 | 1 | 11 | 0 | 0 | 1 |
| Critically Endangered (CR) | 0 | 23 | 300 | 0 | 2 | 21 |
| Endangered (EN) | 38 | 232 | 311 | 3 | 16 | 21 |
| Vulnerable (VU) | 118 | 259 | 168 | 8 | 18 | 12 |
| Near Threatened (NT) | 96 | 345 | 208 | 7 | 24 | 14 |
| Least Concern (LC) | 1205 | 597 | 459 | 83 | 41 | 32 |
| Total of threatened species | 156 | 515 | 790 | 11 | 35 | 54 |

**Table S5** Predicted current suitable habitat for each mammal species, projected change by 2070, and estimated conservation status using three earth system models and two RCPs.

| species | suitable habitat at present (km$^2$) | % of suitable habitat change under RCP2.6 | | | % of suitable habitat change under RCP8.5 | | | mean area change | | IUCN Status | | |
|---|---|---|---|---|---|---|---|---|---|---|---|---|
| | | CNRM-CM5 | GFDL-CM3 | HadGEM2-ES | CNRM-CM5 | GFDL-CM3 | HadGEM2-ES | RCP 2.6 | RCP 8.5 | Current | RCP 2.6 | RCP 8.5 |
| *Aonyx cinerea* | 42,972 | -52 | -80 | -79 | -86 | -93 | -98 | -70 ± 16 | -92 ± 6 | LC | EN | CR |
| *Arctictis binturong* | 78,587 | -51 | -64 | -22 | -68 | -97 | -90 | -45 ± 22 | -85 ± 15 | LC | VU | CR |
| *Arctonyx collaris* | 51,015 | -28 | -39 | -75 | -69 | -94 | -95 | -47 ± 25 | -86 ± 15 | LC | VU | CR |
| *Atherurus macrourus* | 61,397 | -34 | -39 | -64 | -54 | -97 | -93 | -45 ± 16 | -81 ± 24 | LC | VU | CR |
| *Axis porcinus* | 17,565 | 48 | -1 | 227 | 39 | -60 | 437 | 91 ± 120 | 139 ± 263 | VU | LC | LC |
| *Bos gaurus* | 53,695 | -42 | -66 | -46 | -76 | -99 | -88 | -51 ± 13 | -88 ± 11 | LC | EN | CR |
| *Bos javanicus* | 26,253 | -31 | -61 | -38 | -64 | -99 | -95 | -43 ± 16 | -86 ± 19 | NT | VU | CR |
| *Bubalus bubalis* | 5,177 | -49 | -3 | -71 | -40 | -84 | -30 | -41 ± 34 | -51 ± 29 | VU | VU | EN |
| *Callosciurus caniceps* | 123,972 | 15 | 14 | 0 | 56 | 38 | 183 | 10 ± 8 | 92 ± 79 | LC | LC | LC |
| *Callosciurus erythraeus* | 114,430 | -21 | -52 | -24 | 31 | -88 | 7 | -33 ± 17 | -16 ± 63 | LC | VU | NT |
| *Callosciurus finlaysonii* | 134,389 | 3 | -21 | -14 | -25 | -22 | -35 | -11 ± 13 | -27 ± 7 | LC | NT | NT |
| *Canis aureus* | 106,932 | -12 | -48 | -38 | -59 | -69 | -87 | -33 ± 19 | -72 ± 14 | LC | VU | EN |
| *Cannomys badius* | 7,171 | 25 | -98 | -100 | -36 | -64 | -100 | -58 ± 72 | -67 ± 32 | VU | EN | EN |
| *Capricornis milneedwardsii* | 96,226 | -30 | -53 | -45 | -54 | -82 | -86 | -43 ± 11 | -74 ± 18 | LC | VU | EN |
| *Cervus unicolor* | 63,840 | -30 | -52 | -29 | -60 | -91 | -82 | -37 ± 13 | -78 ± 16 | LC | VU | EN |
| *Chaerephon plicatus* | 304,889 | -24 | -19 | -67 | -15 | -35 | 10 | -37 ± 26 | -13 ± 23 | LC | VU | NT |
| *Cuon alpinus* | 79,791 | -25 | -67 | -69 | -64 | -63 | -55 | -54 ± 25 | -61 ± 5 | LC | EN | EN |
| *Cynogale bennettii* | 17,300 | 35 | 435 | 97 | 368 | 979 | 270 | 189 ± 215 | 539 ± 384 | VU | LC | LC |
| *Elephas maximus* | 90,037 | -36 | -59 | -56 | -70 | -97 | -89 | -51 ± 12 | -85 ± 14 | LC | EN | CR |
| *Felis chaus* | 183,946 | -19 | 9 | -29 | -20 | -38 | -71 | -13 ± 20 | -43 ± 26 | LC | NT | VU |
| *Galeopterus variegatus* | 386,145 | 27 | 47 | 41 | 52 | 55 | 57 | 38 ± 10 | 55 ± 2 | LC | LC | LC |
| *Helarctos malayanus* | 63,188 | -42 | -76 | -72 | -81 | -98 | -95 | -63 ± 19 | -91 ± 9 | LC | EN | CR |
| *Herpestes javanicus* | 144,994 | -33 | -88 | -61 | -71 | -96 | -95 | -61 ± 27 | -87 ± 15 | LC | EN | CR |

| | | | | | | | | | | | | |
|---|---|---|---|---|---|---|---|---|---|---|---|---|
| *Herpestes urva* | 79,937 | -18 | -22 | -62 | -41 | -91 | -39 | -34 ± 24 | -57 ± 29 | LC | VU | EN |
| *Hipposideros armiger* | 216,324 | 28 | 60 | 79 | 68 | 72 | 71 | 56 ± 25 | 70 ± 2 | LC | LC | LC |
| *Hipposideros larvatus* | 123,049 | -10 | -51 | -2 | -3 | -13 | -80 | -21 ± 27 | -32 ± 42 | LC | NT | VU |
| *Hylobates agilis* | 17,257 | -32 | -42 | 39 | -78 | -99 | -85 | -11 ± 44 | -87 ± 11 | VU | NT | CR |
| *Hylobates lar* | 43,572 | -31 | -31 | -54 | -78 | -95 | -96 | -38 ± 13 | -89 ± 10 | LC | VU | CR |
| *Hylobates pileatus* | 6,105 | -26 | -57 | -71 | -15 | -80 | -78 | -52 ± 23 | -58 ± 37 | VU | EN | EN |
| *Hystrix brachyura* | 79,682 | -46 | -93 | -83 | -90 | -99 | -98 | -74 ± 25 | -96 ± 5 | LC | EN | CR |
| *Kerivoula hardwickii* | 62,778 | 14 | 62 | 59 | 78 | 79 | 185 | 45 ± 27 | 114 ± 61 | LC | LC | LC |
| *Kerivoula papillosa* | 39,423 | 53 | -10 | 114 | -2 | 134 | 33 | 53 ± 62 | 55 ± 71 | LC | LC | LC |
| *Lepus peguensis* | 46,135 | -31 | -73 | -68 | -87 | -96 | -98 | -57 ± 23 | -94 ± 6 | LC | EN | CR |
| *Macaca arctoides* | 57,697 | -30 | -24 | 37 | -55 | -71 | 76 | -6 ± 37 | -17 ± 81 | LC | NT | NT |
| *Macaca fascicularis* | 107,614 | 27 | 25 | 94 | 137 | 277 | 19 | 49 ± 39 | 144 ± 129 | LC | LC | LC |
| *Macaca leonine* | 31,219 | -38 | -94 | -90 | -86 | -100 | -100 | -74 ± 31 | -95 ± 8 | LC | EN | CR |
| *Macaca mulatta* | 27,325 | -31 | 2 | -100 | -79 | -99 | -100 | -43 ± 52 | -93 ± 12 | NT | VU | CR |
| *Macaca nemestrina* | 9,990 | -32 | -21 | -84 | -69 | -95 | -86 | -46 ± 34 | -83 ± 13 | VU | VU | CR |
| *Manis javanica* | 95,548 | -31 | -53 | -42 | -53 | -15 | -52 | -42 ± 11 | -40 ± 22 | LC | VU | VU |
| *Manis pentadactyla* | 88,005 | -37 | -53 | -50 | -66 | -85 | -74 | -47 ± 8 | -75 ± 10 | LC | VU | EN |
| *Martes flavigula* | 135,070 | -43 | -79 | -38 | -73 | -92 | -72 | -54 ± 22 | -79 ± 11 | LC | EN | EN |
| *Megaderma spasma* | 212,894 | 4 | 32 | 21 | 111 | 153 | -35 | 19 ± 14 | 77 ± 99 | LC | LC | LC |
| *Melogale personata* | 148,624 | 48 | 56 | 62 | 65 | 57 | 106 | 55 ± 7 | 76 ± 27 | LC | LC | LC |
| *Menetes berdmorei* | 149,130 | -44 | -31 | 12 | -53 | -55 | 13 | -21 ± 29 | -32 ± 39 | LC | NT | VU |
| *Muntiacus feae* | 58,273 | -15 | -62 | -29 | -57 | -91 | -80 | -35 ± 24 | -76 ± 17 | LC | VU | EN |
| *Muntiacus muntjak* | 152,554 | -2 | -13 | 12 | -22 | -42 | -50 | -1 ± 13 | -38 ± 14 | LC | NT | VU |
| *Muntiacus vaginalis* | 21,997 | -46 | -87 | -87 | -92 | -86 | -99 | -73 ± 24 | -92 ± 7 | NT | EN | CR |
| *Murina cyclotis* | 228,019 | 22 | 3 | 63 | 33 | 68 | 82 | 30 ± 31 | 61 ± 26 | LC | LC | LC |
| *Murina suilla* | 11,864 | 193 | 159 | 308 | 255 | 230 | 236 | 220 ± 78 | 240 ± 13 | VU | LC | LC |
| *Naemorhedus caudatus* | 38,305 | 0 | 18 | 12 | -17 | -62 | -55 | 10 ± 9 | -45 ± 25 | LC | LC | VU |
| *Neofelis nebulosa* | 158,443 | -30 | 43 | -44 | 46 | -87 | 67 | -10 ± 46 | 9 ± 84 | LC | NT | LC |
| *Panthera pardus* | 30,285 | -46 | -75 | -49 | -92 | -99 | -96 | -56 ± 16 | -96 ± 3 | LC | EN | CR |
| *Panthera tigris* | 22,540 | -31 | -44 | -34 | -82 | -99 | -59 | -37 ± 7 | -80 ± 20 | NT | VU | EN |
| *Paradoxurus hermaphroditus* | 153,673 | -15 | -32 | -43 | -55 | -54 | -87 | -30 ± 14 | -65 ± 19 | LC | VU | EN |
| *Paradoxurus musanga* | 236,831 | -35 | -58 | 6 | -98 | -88 | -99 | -29 ± 32 | -95 ± 6 | LC | NT | CR |
| *Pardofelis temminckii* | 63,837 | 62 | -23 | 42 | 97 | 160 | -74 | 27 ± 45 | 61 ± 121 | LC | LC | LC |

| | | | | | | | | | | | | |
|---|---|---|---|---|---|---|---|---|---|---|---|---|
| *Presbytis femoralis* | 8,052 | -68 | -95 | -54 | -97 | -100 | -84 | -72 ± 21 | -93 ± 9 | VU | EN | CR |
| *Prionailurus bengalensis* | 49,402 | -14 | -61 | -85 | -83 | -63 | -99 | -53 ± 36 | -82 ± 18 | LC | EN | CR |
| *Prionailurus viverrinus* | 116,313 | -61 | -85 | -72 | -97 | -100 | -100 | -73 ± 12 | -99 ± 2 | LC | EN | CR |
| *Prionodon linsang* | 86,388 | -16 | -43 | -53 | -25 | -83 | -84 | -37 ± 19 | -64 ± 34 | LC | VU | EN |
| *Prionodon pardicolor* | 53,710 | -12 | -53 | -70 | -44 | 2 | 11 | -45 ± 30 | -10 ± 30 | LC | VU | NT |
| *Ratufa bicolor* | 63,846 | -38 | -80 | -77 | -75 | -94 | -93 | -65 ± 23 | -87 ± 11 | LC | EN | CR |
| *Rhinolophus acuminatus* | 56,765 | 45 | 68 | 47 | 16 | -12 | 134 | 53 ± 12 | 46 ± 78 | LC | LC | LC |
| *Rhinolophus affinis* | 137,846 | -21 | -16 | -72 | -37 | 9 | 14 | -36 ± 31 | -5 ± 28 | LC | VU | NT |
| *Rhinolophus coelophyllus* | 461,273 | 4 | -27 | -15 | 9 | -11 | 6 | -13 ± 15 | 1 ± 11 | LC | NT | LC |
| *Rhinolophus malayanus* | 463,037 | 6 | -5 | 9 | -2 | 12 | 11 | 3 ± 8 | 7 ± 8 | LC | LC | LC |
| *Rucervus eldii* | 137,849 | -28 | -69 | 32 | -34 | -93 | -1 | -22 ± 51 | -43 ± 46 | LC | NT | VU |
| *Rusa unicolor* | 12,922 | -68 | -57 | -44 | -53 | -77 | -84 | -57 ± 12 | -71 ± 16 | VU | EN | EN |
| *Sus scrofa* | 148,495 | -11 | -19 | -5 | -31 | -56 | -47 | -12 ± 7 | -45 ± 13 | LC | NT | VU |
| *Tamiops macclellandii* | 43,086 | -67 | -93 | -71 | -92 | -100 | -99 | -77 ± 14 | -97 ± 4 | LC | EN | CR |
| *Tapirus indicus* | 22,725 | -70 | -86 | -31 | -88 | -99 | -90 | -62 ± 28 | -92 ± 6 | NT | EN | CR |
| *Trachypithecus obscurus* | 14,716 | -39 | -70 | -47 | -62 | -98 | -94 | -52 ± 16 | -85 ± 19 | VU | EN | CR |
| *Trachypithecus phayrei* | 4,018 | -92 | -87 | -100 | -100 | -100 | -100 | -93 ± 7 | -100 ± 0 | EN | CR | EX |
| *Tragulus kanchil* | 50,681 | -47 | -73 | -62 | -74 | -96 | -93 | -61 ± 13 | -88 ± 12 | LC | EN | CR |
| *Tragulus napu* | 81,635 | -34 | -71 | -37 | -60 | -39 | -32 | -47 ± 21 | -44 ± 14 | LC | VU | VU |
| *Tupaia belangeri* | 82,199 | 7 | 5 | 84 | -2 | 61 | 20 | 32 ± 45 | 27 ± 32 | LC | LC | LC |
| *Tupaia minor* | 414 | 68 | 456 | 424 | 429 | 1931 | 562 | 316 ± 215 | 974 ± 831 | EN | LC | LC |
| *Ursus thibetanus* | 89,116 | -30 | -72 | -57 | -71 | -95 | -93 | -53 ± 21 | -87 ± 13 | LC | EN | CR |
| *Viverra zibetha* | 143,500 | -16 | -71 | -48 | -72 | -91 | -96 | -45 ± 28 | -87 ± 13 | LC | VU | CR |
| *Viverricula indica* | 49,553 | 9 | -49 | -39 | -30 | -41 | -88 | -26 ± 31 | -53 ± 31 | LC | NT | EN |

**Table S6** Predicted current suitable habitat for each bird species, projected change by 2070, and estimated conservation status using three earth system models and two RCPs.

| species | suitable habitat at present ($km^2$) | % of suitable habitat change under RCP2.6 | | | % of suitable habitat change under RCP8.5 | | | mean area change | | IUCN Status | | |
|---|---|---|---|---|---|---|---|---|---|---|---|---|
| | | CNRM-CM5 | GFDL-CM3 | HadGEM2-ES | CNRM-CM5 | GFDL-CM3 | HadGEM2-ES | RCP2.6 | RCP8.5 | Current | RCP 2.6 | RCP 8.5 |
| *Accipiter badius* | 357,503 | -1 | 1 | 8 | -28 | -40 | 22 | 3 ± 5 | -15 ± 33 | LC | LC | NT |
| *Accipiter gularis* | 209,757 | 24 | -34 | 27 | -40 | -14 | 24 | 6 ± 34 | -10 ± 32 | LC | LC | NT |
| *Accipiter nisus* | 34,246 | -71 | -77 | 38 | -80 | -93 | -92 | -37 ± 65 | -88 ± 7 | LC | VU | CR |
| *Accipiter soloensis* | 182,011 | -24 | -32 | 30 | -67 | -63 | -94 | -9 ± 34 | -75 ± 17 | LC | NT | EN |
| *Accipiter trivirgatus* | 203,040 | -31 | -54 | -36 | -65 | -83 | -84 | -41 ± 12 | -77 ± 11 | LC | VU | EN |
| *Accipiter virgatus* | 163,588 | -45 | -68 | -53 | -80 | -97 | -95 | -55 ± 12 | -91 ± 9 | LC | EN | CR |
| *Aceros nipalensis* | 36,878 | -15 | 27 | 32 | 19 | -73 | 68 | 15 ± 26 | 5 ± 72 | LC | LC | LC |
| *Aceros subruficollis* | 41,997 | -63 | -92 | -67 | -82 | -99 | -98 | -74 ± 16 | -93 ± 10 | LC | EN | CR |
| *Acridotheres burmannicus* | 76,494 | -38 | -66 | -35 | -78 | -64 | -20 | -46 ± 17 | -54 ± 30 | LC | VU | EN |
| *Actenoides concretus* | 31,141 | -14 | -35 | -23 | -34 | -87 | -72 | -24 ± 11 | -64 ± 27 | LC | NT | EN |
| *Actinodura ramsayi* | 10,862 | -63 | -98 | -45 | -95 | -100 | -100 | -69 ± 27 | -98 ± 3 | VU | EN | CR |
| *Actitis hypoleucos* | 217,071 | 18 | 52 | 52 | -53 | -12 | -10 | 41 ± 19 | -25 ± 24 | LC | LC | NT |
| *Aerodramus brevirostris* | 214,964 | -50 | -58 | -39 | -74 | -90 | -89 | -49 ± 10 | -84 ± 9 | LC | VU | CR |
| *Aerodramus fuciphagus* | 173,819 | 16 | 28 | 43 | -52 | 2 | 64 | 29 ± 13 | 5 ± 58 | LC | LC | LC |
| *Aethopyga gouldiae* | 17,693 | -46 | -60 | 4 | -51 | -94 | -96 | -34 ± 34 | -80 ± 25 | VU | VU | EN |
| *Aethopyga ignicauda* | 107,831 | -29 | -25 | 0 | -58 | -78 | -73 | -18 ± 16 | -69 ± 11 | LC | NT | EN |
| *Aethopyga nipalensis* | 20,650 | -49 | -94 | 8 | -95 | -100 | -98 | -45 ± 51 | -98 ± 3 | NT | VU | CR |
| *Aethopyga saturata* | 89,400 | -45 | -80 | -38 | -80 | -94 | -92 | -54 ± 23 | -89 ± 8 | LC | EN | CR |
| *Aethopyga siparaja* | 151,291 | -23 | -63 | 6 | -72 | -25 | -16 | -27 ± 34 | -37 ± 30 | LC | NT | VU |
| *Agropsar philippensis* | 127,957 | -85 | -92 | -92 | -97 | -99 | -100 | -90 ± 4 | -99 ± 2 | LC | CR | CR |
| *Agropsar sturninus* | 167,636 | 3 | 5 | 40 | -16 | 17 | -26 | 16 ± 21 | -8 ± 22 | LC | LC | NT |
| *Alcedo atthis* | 345,937 | 3 | 26 | 26 | 6 | 5 | 20 | 18 ± 13 | 10 ± 8 | LC | LC | LC |
| *Alcedo euryzona* | 31,011 | -32 | -27 | -32 | -59 | -84 | -56 | -30 ± 2 | -66 ± 15 | LC | VU | EN |
| *Alcedo meninting* | 148,949 | -56 | -74 | -71 | -78 | -98 | -97 | -67 ± 10 | -91 ± 11 | LC | EN | CR |

| | | | | | | | | | | | | |
|---|---|---|---|---|---|---|---|---|---|---|---|---|
| *Alcippe brunneicauda* | 13,217 | 30 | 111 | 99 | 21 | 55 | 62 | 80 ± 44 | 46 ± 22 | VU | LC | LC |
| *Alcippe castaneceps* | 19,907 | -46 | -84 | -50 | -92 | -96 | -96 | -60 ± 21 | -95 ± 2 | VU | EN | CR |
| *Alcippe fratercula* | 37,833 | -35 | -76 | 6 | -70 | -90 | -74 | -35 ± 41 | -78 ± 10 | LC | VU | EN |
| *Alcippe poioicephala* | 123,946 | -51 | -75 | -51 | -85 | -97 | -95 | -59 ± 14 | -93 ± 7 | LC | EN | CR |
| *Alcippe rufogularis* | 49,175 | -43 | -68 | -8 | -80 | -100 | -89 | -40 ± 30 | -90 ± 10 | LC | VU | CR |
| *Alophoixus finschii* | 11,106 | 169 | 259 | 495 | -2 | 902 | 785 | 308 ± 168 | 562 ± 491 | VU | LC | LC |
| *Alophoixus flaveolus* | 35,716 | -11 | -31 | -7 | -52 | -100 | -8 | -16 ± 13 | -53 ± 46 | LC | NT | EN |
| *Alophoixus ochraceus* | 41,887 | -36 | -65 | -61 | -78 | -93 | -94 | -54 ± 16 | -88 ± 9 | LC | EN | CR |
| *Alophoixus pallidus* | 79,586 | -32 | -54 | -21 | -62 | -69 | -71 | -36 ± 17 | -67 ± 5 | LC | VU | EN |
| *Alophoixus phaeocephalus* | 33,905 | -33 | -48 | -46 | -22 | -41 | -25 | -42 ± 8 | -29 ± 11 | LC | VU | NT |
| *Amandava amandava* | 106,461 | 65 | 121 | 159 | 105 | 187 | 254 | 115 ± 47 | 182 ± 75 | LC | LC | LC |
| *Amaurornis phoenicurus* | 310,321 | 4 | 16 | 27 | -54 | -21 | 8 | 15 ± 11 | -22 ± 31 | LC | LC | NT |
| *Ampeliceps coronatus* | 93,060 | -38 | -71 | -65 | -82 | -88 | -97 | -58 ± 18 | -89 ± 8 | LC | EN | CR |
| *Anas acuta* | 49,967 | 66 | 383 | 248 | 81 | -6 | 21 | 233 ± 159 | 32 ± 45 | LC | LC | LC |
| *Anas clypeata* | 69,423 | 13 | 176 | 148 | -6 | -26 | -71 | 112 ± 87 | -34 ± 34 | LC | LC | VU |
| *Anas crecca* | 73,417 | 177 | 357 | 300 | 104 | 108 | 185 | 278 ± 92 | 132 ± 46 | LC | LC | LC |
| *Anas penelope* | 43,360 | 14 | -38 | 16 | -84 | -94 | -78 | -3 ± 31 | -85 ± 8 | LC | NT | CR |
| *Anas poecilorhyncha* | 51,189 | 338 | 540 | 408 | 183 | 405 | 207 | 429 ± 103 | 265 ± 122 | LC | LC | LC |
| *Anas querquedula* | 141,593 | 49 | 130 | 128 | 106 | 208 | -92 | 103 ± 46 | 74 ± 153 | LC | LC | LC |
| *Anas strepera* | 201,980 | 54 | 70 | 25 | 18 | -25 | 19 | 50 ± 23 | 4 ± 25 | LC | LC | LC |
| *Anastomus oscitans* | 236,195 | 26 | 26 | 46 | 11 | -18 | 19 | 33 ± 11 | 4 ± 20 | LC | LC | LC |
| *Anhinga melanogaster* | 201,015 | 7 | 5 | 2 | -38 | -55 | -46 | 5 ± 3 | -46 ± 8 | LC | LC | VU |
| *Anorrhinus austeni* | 4,471 | -79 | -80 | -100 | -73 | -99 | -100 | -86 ± 12 | -91 ± 15 | EN | CR | CR |
| *Anorrhinus galeritus* | 11,186 | 26 | -13 | 23 | -2 | -64 | -54 | 12 ± 22 | -40 ± 33 | VU | LC | VU |
| *Anorrhinus tickelli* | 25,577 | -72 | -88 | -81 | -93 | -100 | -100 | -81 ± 8 | -98 ± 4 | NT | CR | CR |
| *Anthipes monileger* | 10,074 | -60 | -85 | -59 | -83 | -97 | -99 | -68 ± 15 | -93 ± 9 | VU | EN | CR |
| *Anthipes solitaris* | 34,280 | -51 | -67 | -58 | -81 | -95 | -85 | -59 ± 8 | -87 ± 7 | LC | EN | CR |
| *Anthracoceros albirostris* | 114,012 | -43 | -63 | -60 | -62 | -92 | -96 | -55 ± 11 | -83 ± 18 | LC | EN | CR |
| *Anthracoceros malayanus* | 25,467 | -7 | 18 | 8 | -22 | -58 | -46 | 6 ± 12 | -42 ± 19 | NT | LC | VU |
| *Anthreptes malacensis* | 278,271 | -12 | -48 | 9 | -51 | -41 | 11 | -17 ± 29 | -27 ± 33 | LC | NT | NT |
| *Anthreptes rhodolaemus* | 38,427 | 6 | 9 | 50 | -26 | 6 | -36 | 22 ± 24 | -19 ± 22 | LC | LC | NT |
| *Anthreptes simplex* | 43,236 | -44 | -28 | -26 | -38 | -56 | -85 | -32 ± 10 | -60 ± 24 | LC | VU | EN |
| *Anthus cervinus* | 182,844 | -49 | -83 | -57 | -83 | -94 | -93 | -63 ± 18 | -90 ± 6 | LC | EN | CR |

| | | | | | | | | | | | | |
|---|---|---|---|---|---|---|---|---|---|---|---|---|
| *Anthus hodgsoni* | 154,978 | -36 | -50 | -21 | -57 | -78 | -86 | -36 ± 14 | -74 ± 15 | LC | VU | EN |
| *Anthus richardi* | 246,286 | 7 | -37 | 31 | -10 | 8 | -18 | 0 ± 35 | -7 ± 13 | LC | LC | NT |
| *Anthus roseatus* | 138,012 | -51 | -10 | -53 | -67 | -97 | -70 | -38 ± 24 | -78 ± 16 | LC | VU | EN |
| *Anthus rufulus* | 325,002 | 0 | 7 | -4 | -18 | -11 | -21 | 1 ± 5 | -17 ± 5 | LC | LC | NT |
| *Aplonis panayensis* | 29,599 | -46 | -45 | -16 | -56 | -79 | -86 | -36 ± 17 | -74 ± 15 | NT | VU | EN |
| *Apus nipalensis* | 301,039 | 6 | 28 | 47 | 1 | 2 | 30 | 27 ± 21 | 11 ± 17 | LC | LC | LC |
| *Apus pacificus* | 235,455 | -1 | -10 | 20 | -71 | -17 | -89 | 3 ± 15 | -59 ± 38 | LC | LC | EN |
| *Aquila clanga* | 144,407 | -30 | -51 | 8 | -70 | -71 | -52 | -24 ± 30 | -64 ± 11 | LC | NT | EN |
| *Aquila heliaca* | 49,801 | -71 | -81 | -48 | -95 | -99 | -100 | -67 ± 17 | -98 ± 2 | LC | EN | CR |
| *Aquila nipalensis* | 116,117 | -65 | -78 | -73 | -30 | -97 | -99 | -72 ± 7 | -75 ± 39 | LC | EN | EN |
| *Arachnothera chrysogenys* | 30,035 | -40 | -31 | -47 | -70 | -88 | -86 | -39 ± 8 | -81 ± 10 | LC | VU | CR |
| *Arachnothera crassirostris* | 11,532 | 75 | 163 | 174 | 133 | 124 | 67 | 137 ± 54 | 108 ± 36 | VU | LC | LC |
| *Arachnothera flavigaster* | 39,336 | -36 | -20 | -18 | -47 | -29 | -45 | -25 ± 10 | -41 ± 10 | LC | NT | VU |
| *Arachnothera longirostra* | 225,652 | -33 | -68 | -15 | -62 | -58 | -52 | -39 ± 27 | -57 ± 5 | LC | VU | EN |
| *Arachnothera magna* | 97,682 | -52 | -71 | -34 | -78 | -95 | -66 | -52 ± 18 | -79 ± 14 | LC | EN | EN |
| *Arachnothera modesta* | 10,240 | 119 | -44 | 222 | -18 | -10 | 68 | 99 ± 134 | 13 ± 47 | VU | LC | LC |
| *Arachnothera robusta* | 35,042 | 24 | 46 | 67 | 14 | 48 | -32 | 46 ± 22 | 10 ± 40 | LC | LC | LC |
| *Arborophila brunneopectus* | 67,597 | -57 | -86 | -63 | -94 | -97 | -100 | -69 ± 15 | -97 ± 3 | LC | EN | CR |
| *Arborophila chloropus* | 72,360 | -49 | -93 | -77 | -91 | -99 | -100 | -73 ± 22 | -97 ± 5 | LC | EN | CR |
| *Arborophila rufogularis* | 36,084 | -52 | -100 | -68 | -99 | -100 | -99 | -73 ± 25 | -99 ± 1 | LC | EN | CR |
| *Ardea alba* | 237,347 | 21 | 44 | 42 | -30 | 2 | 56 | 36 ± 13 | 9 ± 43 | LC | LC | LC |
| *Ardea cinerea* | 193,010 | 21 | 91 | 47 | -21 | -34 | -88 | 53 ± 36 | -47 ± 35 | LC | LC | VU |
| *Ardea modesta* | 31,969 | -41 | -27 | -25 | -71 | -81 | -80 | -31 ± 9 | -77 ± 5 | LC | VU | EN |
| *Ardea purpurea* | 161,025 | 49 | 13 | 37 | -58 | -35 | 137 | 33 ± 19 | 14 ± 106 | LC | LC | LC |
| *Ardeola bacchus* | 362,547 | 7 | 4 | 19 | -26 | -4 | 19 | 10 ± 8 | -4 ± 22 | LC | LC | NT |
| *Ardeola grayii* | 82,471 | 163 | 253 | 321 | 190 | 427 | 559 | 245 ± 79 | 392 ± 187 | LC | LC | LC |
| *Ardeola speciosa* | 82,521 | 46 | 56 | 67 | 7 | 186 | 116 | 56 ± 11 | 103 ± 90 | LC | LC | LC |
| *Arenaria interpres* | 91,096 | -21 | 87 | -8 | -3 | 56 | -68 | 19 ± 59 | -5 ± 62 | LC | LC | NT |
| *Argusianus argus* | 17,763 | -16 | -13 | 34 | 11 | -43 | -43 | 2 ± 28 | -25 ± 31 | VU | LC | NT |
| *Artamus fuscus* | 302,459 | -5 | -23 | 19 | -36 | -46 | -36 | -3 ± 21 | -39 ± 6 | LC | NT | VU |
| *Asarcornis scutulata* | 157,715 | -7 | -72 | -28 | -62 | -100 | -54 | -36 ± 33 | -72 ± 25 | LC | VU | EN |
| *Asio flammeus* | 340,313 | 28 | 9 | 18 | 11 | -23 | 23 | 18 ± 10 | 4 ± 24 | LC | LC | LC |
| *Athene brama* | 209,770 | 35 | 30 | 22 | -20 | -49 | 69 | 29 ± 6 | 0 ± 61 | LC | LC | LC |

| | | | | | | | | | | | | |
|---|---|---|---|---|---|---|---|---|---|---|---|---|
| *Aviceda jerdoni* | 152,212 | -26 | -62 | -46 | -52 | -92 | 58 | -45 ± 18 | -29 ± 77 | LC | VU | NT |
| *Aviceda leuphotes* | 282,197 | 11 | 17 | 18 | -10 | -7 | -7 | 16 ± 4 | -8 ± 2 | LC | LC | NT |
| *Aythya baeri* | 97,546 | -32 | 60 | 89 | 34 | -25 | -77 | 39 ± 63 | -23 ± 56 | LC | LC | NT |
| *Aythya ferina* | 119,496 | 31 | 77 | 63 | 49 | 51 | -11 | 57 ± 23 | 30 ± 35 | LC | LC | LC |
| *Aythya fuligula* | 197,057 | -3 | -58 | -76 | -55 | -95 | -84 | -46 ± 38 | -78 ± 21 | LC | VU | EN |
| *Aythya nyroca* | 5,089 | 638 | 3972 | 2295 | 902 | 4674 | 4736 | 2302 ± 1667 | 3437 ± 2196 | VU | LC | LC |
| *Bambusicola fytchii* | 6,589 | -76 | -88 | -66 | -94 | -100 | -99 | -77 ± 11 | -98 ± 3 | VU | EN | CR |
| *Batrachostomus affinis* | 79,821 | -31 | -49 | -45 | -58 | -91 | -91 | -42 ± 9 | -80 ± 19 | LC | VU | EN |
| *Batrachostomus hodgsoni* | 10,642 | -72 | -91 | -76 | -98 | -100 | -99 | -80 ± 10 | -99 ± 1 | VU | EN | CR |
| *Batrachostomus stellatus* | 60,398 | 37 | 21 | 42 | 7 | 13 | 65 | 33 ± 11 | 28 ± 32 | LC | LC | LC |
| *Berenicornis comatus* | 30,796 | -12 | -30 | -24 | -57 | -94 | -83 | -22 ± 9 | -78 ± 19 | LC | NT | EN |
| *Blythipicus pyrrhotis* | 64,732 | -53 | -78 | -39 | -85 | -97 | -94 | -57 ± 20 | -92 ± 6 | LC | EN | CR |
| *Blythipicus rubiginosus* | 41,552 | -35 | -52 | -43 | -60 | -87 | -82 | -43 ± 8 | -76 ± 14 | LC | VU | EN |
| *Botaurus stellaris* | 32,249 | 48 | -61 | 251 | -83 | 194 | -86 | 79 ± 158 | 9 ± 161 | LC | LC | LC |
| *Brachypodius eutilotus* | 27,491 | -7 | 39 | 10 | 13 | -4 | -42 | 14 ± 23 | -11 ± 28 | NT | LC | NT |
| *Brachypteryx leucophris* | 12,630 | -57 | -66 | 96 | -78 | -89 | -70 | -9 ± 91 | -79 ± 9 | VU | NT | EN |
| *Brachypteryx montana* | 10,600 | -49 | -84 | -37 | -93 | -100 | -86 | -57 ± 25 | -93 ± 7 | VU | EN | CR |
| *Bubo nipalensis* | 16,426 | -53 | -74 | -87 | -86 | -91 | -99 | -71 ± 17 | -92 ± 6 | VU | EN | CR |
| *Bubo sumatranus* | 16,403 | 86 | 61 | 128 | 92 | 21 | 79 | 92 ± 34 | 64 ± 38 | VU | LC | LC |
| *Bubulcus coromandus* | 159,612 | 10 | -60 | -41 | -55 | -93 | 95 | -30 ± 36 | -18 ± 99 | LC | VU | NT |
| *Bubulcus ibis* | 315,614 | 14 | 24 | 29 | 18 | -2 | 25 | 22 ± 8 | 13 ± 14 | LC | LC | LC |
| *Buceros bicornis* | 73,300 | -37 | -63 | -73 | -86 | -95 | -96 | -58 ± 19 | -92 ± 6 | LC | EN | CR |
| *Buceros rhinoceros* | 18,658 | 26 | -25 | 11 | 5 | 4 | -20 | 4 ± 26 | -4 ± 14 | VU | LC | NT |
| *Buceros vigil* | 54,458 | -8 | -42 | -28 | -63 | -81 | -87 | -26 ± 17 | -77 ± 13 | LC | NT | EN |
| *Burhinus indicus* | 90,274 | 91 | 96 | 176 | 14 | 161 | 430 | 121 ± 48 | 202 ± 211 | LC | LC | LC |
| *Butastur indicus* | 247,751 | 6 | -23 | -13 | -43 | -66 | -2 | -10 ± 15 | -37 ± 32 | LC | NT | VU |
| *Butastur liventer* | 250,336 | -32 | -29 | -31 | -61 | -74 | -69 | -31 ± 1 | -68 ± 6 | LC | VU | EN |
| *Buteo buteo* | 103,744 | -48 | -60 | -26 | -74 | -94 | -92 | -45 ± 17 | -87 ± 11 | LC | VU | CR |
| *Buteo japonicus* | 252,523 | -37 | -68 | -48 | -74 | -90 | -80 | -51 ± 16 | -81 ± 8 | LC | EN | CR |
| *Buteo refectus* | 90,247 | -41 | -72 | -45 | -85 | -94 | -91 | -53 ± 17 | -90 ± 4 | LC | EN | CR |
| *Butorides striata* | 242,794 | 3 | 1 | 28 | -60 | -25 | -81 | 11 ± 15 | -55 ± 28 | LC | LC | EN |
| *Cacomantis merulinus* | 331,048 | 2 | 21 | 10 | -14 | -27 | 52 | 11 ± 10 | 3 ± 42 | LC | LC | LC |
| *Cacomantis sonneratii* | 170,821 | -49 | -71 | -62 | -82 | -95 | -93 | -61 ± 11 | -90 ± 7 | LC | EN | CR |

| | | | | | | | | | | | | |
|---|---|---|---|---|---|---|---|---|---|---|---|---|
| *Cacomantis variolosus* | 53,565 | 20 | 5 | -24 | -44 | -13 | -16 | 0 ± 23 | -24 ± 17 | LC | LC | NT |
| *Calidris alba* | 329,381 | 0 | -3 | 7 | -14 | -33 | 18 | 1 ± 5 | -10 ± 26 | LC | LC | NT |
| *Calidris alpina* | 112,105 | 132 | 209 | 227 | -21 | 38 | 205 | 189 ± 51 | 74 ± 117 | LC | LC | LC |
| *Calidris canutus* | 2,156 | -52 | -61 | -54 | -93 | -62 | -100 | -55 ± 5 | -85 ± 20 | EN | EN | CR |
| *Calidris ferruginea* | 55,183 | 108 | 216 | 285 | -3 | 176 | -78 | 203 ± 89 | 32 ± 131 | LC | LC | LC |
| *Calidris minuta* | 31,583 | -42 | 68 | 87 | 151 | -3 | 1 | 38 ± 70 | 50 ± 88 | LC | LC | LC |
| *Calidris pygmaea* | 5,346 | -41 | 25 | -97 | -92 | -99 | 12 | -37 ± 61 | -60 ± 62 | VU | VU | EN |
| *Calidris ruficollis* | 69,881 | 126 | 63 | 48 | 8 | 7 | -8 | 79 ± 41 | 2 ± 9 | LC | LC | LC |
| *Calidris subminuta* | 44,533 | 81 | -3 | 100 | 16 | 179 | -61 | 59 ± 55 | 45 ± 123 | LC | LC | LC |
| *Calidris temminckii* | 98,751 | -19 | 15 | 15 | -49 | -82 | -100 | 4 ± 20 | -77 ± 26 | LC | LC | EN |
| *Calidris tenuirostris* | 8,722 | -35 | 62 | 299 | -1 | 789 | -90 | 109 ± 172 | 233 ± 484 | VU | LC | LC |
| *Caloenas nicobarica* | 50,659 | 1 | -73 | -54 | -76 | -95 | -92 | -42 ± 38 | -88 ± 10 | LC | VU | CR |
| *Caloperdix oculeus* | 6,077 | -75 | -100 | -99 | -100 | -100 | -100 | -91 ± 14 | -100 ± 0 | VU | CR | EX |
| *Caloramphus fuliginosus* | 21,516 | -16 | -31 | -18 | -61 | -79 | -79 | -22 ± 8 | -73 ± 11 | NT | NT | EN |
| *Caprimulgus affinis* | 187,402 | 35 | 87 | 109 | 67 | 117 | 186 | 77 ± 38 | 123 ± 60 | LC | LC | LC |
| *Caprimulgus asiaticus* | 126,707 | 43 | 43 | 105 | 27 | 43 | 249 | 64 ± 36 | 107 ± 124 | LC | LC | LC |
| *Caprimulgus jotaka* | 248,103 | 3 | -17 | 7 | -39 | -22 | -69 | -3 ± 13 | -43 ± 24 | LC | NT | VU |
| *Caprimulgus macrurus* | 205,767 | 4 | 1 | -18 | -39 | 5 | 83 | -4 ± 12 | 16 ± 61 | LC | NT | LC |
| *Carpococcyx renauldi* | 20,553 | -18 | -59 | -52 | -15 | -61 | -39 | -43 ± 22 | -39 ± 23 | NT | VU | VU |
| *Cecropis badia* | 20,949 | 154 | 284 | 209 | 307 | 357 | 345 | 215 ± 65 | 336 ± 26 | NT | LC | LC |
| *Cecropis daurica* | 292,311 | -41 | -79 | 9 | -63 | -49 | -19 | -37 ± 44 | -43 ± 22 | LC | VU | VU |
| *Cecropis striolata* | 208,167 | -45 | -75 | -68 | -80 | -94 | -95 | -62 ± 16 | -90 ± 9 | LC | EN | CR |
| *Celeus brachyurus* | 176,368 | -36 | -19 | 23 | -23 | -64 | -46 | -11 ± 30 | -44 ± 21 | LC | NT | VU |
| *Centropus bengalensis* | 315,073 | 7 | 7 | 16 | -34 | 13 | 55 | 10 ± 5 | 11 ± 44 | LC | LC | LC |
| *Centropus sinensis* | 397,937 | 1 | 16 | 15 | 8 | -14 | 32 | 11 ± 8 | 9 ± 23 | LC | LC | LC |
| *Cerasophila thompsoni* | 43,776 | -56 | -45 | 21 | -68 | -97 | -90 | -27 ± 42 | -85 ± 15 | LC | NT | CR |
| *Ceryle rudis* | 95,365 | 57 | 74 | 47 | -31 | -67 | -45 | 59 ± 14 | -48 ± 18 | LC | LC | VU |
| *Ceyx erithaca* | 118,281 | 19 | 71 | 131 | 80 | 83 | 186 | 74 ± 56 | 116 ± 60 | LC | LC | LC |
| *Chaimarrornis leucocephalus* | 46,550 | -46 | -53 | 34 | -24 | -63 | -49 | -22 ± 49 | -45 ± 20 | LC | NT | VU |
| *Chalcoparia singalensis* | 266,549 | -22 | -63 | -10 | -47 | -50 | -51 | -32 ± 28 | -49 ± 2 | LC | VU | VU |
| *Chalcophaps indica* | 186,985 | -42 | -55 | -39 | -71 | -86 | -58 | -45 ± 9 | -72 ± 14 | LC | VU | EN |
| *Charadrius alexandrinus* | 73,814 | 32 | 129 | 106 | -61 | -21 | 245 | 89 ± 51 | 54 ± 166 | LC | LC | LC |
| *Charadrius dubius* | 218,628 | 6 | 59 | 44 | -11 | 73 | 90 | 36 ± 27 | 51 ± 54 | LC | LC | LC |

| | | | | | | | | | | | | |
|---|---|---|---|---|---|---|---|---|---|---|---|---|
| *Charadrius leschenaultii* | 37,291 | 306 | 583 | 523 | 528 | 1035 | 1086 | 471 ± 145 | 883 ± 309 | LC | LC | LC |
| *Charadrius mongolus* | 23,860 | 268 | 467 | 524 | 482 | 354 | 1158 | 420 ± 134 | 665 ± 432 | NT | LC | LC |
| *Charadrius peronii* | 29,763 | -67 | -89 | -78 | -92 | -99 | -100 | -78 ± 11 | -97 ± 4 | NT | EN | CR |
| *Charadrius veredus* | 68,459 | 146 | 422 | 452 | 93 | 53 | 262 | 340 ± 169 | 136 ± 111 | LC | LC | LC |
| *Chlidonias hybrida* | 43,934 | 115 | 81 | 237 | 27 | 463 | 261 | 144 ± 82 | 250 ± 218 | LC | LC | LC |
| *Chlidonias leucopterus* | 82,255 | 82 | 45 | 134 | -33 | 298 | -55 | 87 ± 45 | 70 ± 198 | LC | LC | LC |
| *Chloropsis aurifrons* | 146,017 | -22 | -38 | -9 | -61 | -79 | -57 | -23 ± 14 | -66 ± 11 | LC | NT | EN |
| *Chroicocephalus brunnicephalus* | 7,308 | 79 | 289 | 342 | 83 | 554 | 689 | 237 ± 139 | 442 ± 318 | VU | LC | LC |
| *Chroicocephalus genei* | 54,114 | 49 | 44 | 287 | 123 | 157 | 606 | 127 ± 139 | 295 ± 269 | LC | LC | LC |
| *Chroicocephalus ridibundus* | 10,992 | 4 | 303 | 700 | 95 | 199 | 36 | 336 ± 349 | 110 ± 83 | VU | LC | LC |
| *Chrysococcyx maculatus* | 136,298 | -25 | -54 | 13 | -60 | -66 | 41 | -22 ± 33 | -28 ± 60 | LC | NT | NT |
| *Chrysococcyx minutillus* | 24,025 | 29 | 4 | -1 | 18 | -65 | -75 | 11 ± 16 | -41 ± 51 | NT | LC | VU |
| *Chrysococcyx xanthorhynchus* | 207,356 | 10 | 10 | 36 | -34 | -10 | -26 | 18 ± 15 | -23 ± 12 | LC | LC | NT |
| *Chrysocolaptes lucidus* | 152,278 | -49 | -69 | -68 | -82 | -98 | -98 | -62 ± 11 | -93 ± 9 | LC | EN | CR |
| *Chrysophlegma mentale* | 60,729 | 12 | -45 | 13 | -25 | -76 | -66 | -7 ± 33 | -56 ± 27 | LC | NT | EN |
| *Ciconia nigra* | 167,931 | 20 | -3 | 13 | 1 | -62 | 143 | 10 ± 12 | 27 ± 105 | LC | LC | LC |
| *Cinnyris asiaticus* | 225,493 | -20 | -25 | -13 | -54 | -61 | -67 | -19 ± 6 | -61 ± 7 | LC | NT | EN |
| *Cinnyris jugularis* | 364,831 | -16 | -56 | 11 | -1 | -5 | 31 | -20 ± 34 | 8 ± 19 | LC | NT | LC |
| *Circaetus gallicus* | 192,316 | 20 | 59 | 58 | 52 | 16 | 79 | 46 ± 22 | 49 ± 32 | LC | LC | LC |
| *Circus aeruginosus* | 110,508 | 9 | -46 | -82 | -87 | -99 | -69 | -40 ± 46 | -85 ± 15 | LC | VU | CR |
| *Circus cyaneus* | 270,999 | -2 | -40 | -40 | -91 | -68 | -91 | -28 ± 22 | -83 ± 14 | LC | NT | CR |
| *Circus melanoleucos* | 213,493 | -8 | 50 | 15 | -44 | -59 | -87 | 19 ± 29 | -63 ± 22 | LC | LC | EN |
| *Circus spilonotus* | 155,414 | 47 | 127 | 76 | 21 | 174 | -87 | 83 ± 40 | 36 ± 131 | LC | LC | LC |
| *Cissa chinensis* | 116,333 | -42 | -30 | -3 | -66 | -59 | 85 | -25 ± 20 | -13 ± 85 | LC | NT | NT |
| *Cisticola exilis* | 156,509 | 46 | 36 | 100 | 72 | 17 | 135 | 61 ± 35 | 74 ± 59 | LC | LC | LC |
| *Cisticola juncidis* | 200,874 | 7 | 1 | -35 | 7 | 72 | 77 | -9 ± 23 | 52 ± 39 | LC | NT | LC |
| *Clamator coromandus* | 185,346 | -17 | -6 | 24 | -39 | -52 | -91 | 0 ± 21 | -61 ± 27 | LC | LC | EN |
| *Clamator jacobinus* | 98,290 | 61 | 94 | 6 | 188 | 112 | 313 | 54 ± 45 | 204 ± 102 | LC | LC | LC |
| *Collocalia affinis* | 27,637 | 23 | 61 | 102 | 77 | -8 | -54 | 62 ± 40 | 5 ± 66 | NT | LC | LC |
| *Columba hodgsonii* | 21,432 | -40 | -97 | -88 | -94 | -100 | -93 | -75 ± 30 | -96 ± 4 | NT | EN | CR |
| *Columba livia* | 325,162 | 4 | 23 | 25 | 22 | -7 | 34 | 17 ± 12 | 16 ± 21 | LC | LC | LC |
| *Columba pulchricollis* | 59,696 | -37 | -98 | 26 | -89 | -93 | -99 | -36 ± 62 | -94 ± 5 | LC | VU | CR |
| *Columba punicea* | 46,223 | -27 | -88 | -87 | -100 | -86 | -91 | -67 ± 35 | -92 ± 7 | LC | EN | CR |

| | | | | | | | | | | | | |
|---|---|---|---|---|---|---|---|---|---|---|---|---|
| *Copsychus malabaricus* | 304,193 | 0 | -31 | 28 | 11 | 39 | 37 | -1 ± 29 | 29 ± 16 | LC | NT | LC |
| *Copsychus saularis* | 354,426 | -2 | -6 | 23 | -7 | -26 | 15 | 5 ± 16 | -6 ± 21 | LC | LC | NT |
| *Coracias affinis* | 24,825 | -30 | -35 | 8 | -51 | -57 | -100 | -19 ± 24 | -69 ± 27 | NT | NT | EN |
| *Coracias benghalensis* | 321,355 | -3 | -23 | -30 | -66 | -69 | -41 | -19 ± 14 | -59 ± 16 | LC | NT | EN |
| *Coracina fimbriata* | 42,184 | 2 | -8 | -40 | -49 | -16 | -7 | -15 ± 22 | -24 ± 22 | LC | NT | NT |
| *Coracina macei* | 124,375 | -30 | -36 | -30 | -62 | -75 | -73 | -32 ± 3 | -70 ± 7 | LC | VU | EN |
| *Coracina melaschistos* | 204,338 | -5 | 21 | 52 | -29 | -28 | 58 | 23 ± 28 | 0 ± 50 | LC | LC | LC |
| *Coracina polioptera* | 173,596 | -29 | -35 | -30 | -44 | -77 | -86 | -31 ± 3 | -69 ± 22 | LC | VU | EN |
| *Corvus levaillantii* | 23,813 | 176 | 490 | 540 | 287 | 22 | 834 | 402 ± 197 | 381 ± 414 | NT | LC | LC |
| *Corvus macrorhynchos* | 355,781 | 11 | -14 | 31 | 31 | 6 | 38 | 10 ± 23 | 25 ± 17 | LC | LC | LC |
| *Corvus splendens* | 44,486 | 98 | 69 | 229 | 154 | 130 | 152 | 132 ± 85 | 145 ± 13 | LC | LC | LC |
| *Coturnix chinensis* | 78,484 | -59 | -91 | -66 | -90 | -99 | -99 | -72 ± 17 | -96 ± 5 | LC | EN | CR |
| *Coturnix coromandelica* | 72,187 | -16 | 119 | 71 | 8 | 132 | 337 | 58 ± 69 | 159 ± 166 | LC | LC | LC |
| *Crypsirina temia* | 274,077 | 2 | -44 | 5 | -45 | -44 | -24 | -12 ± 27 | -37 ± 12 | LC | NT | VU |
| *Cuculus canorus* | 457,024 | -2 | -18 | 9 | 4 | -6 | 15 | -3 ± 14 | 4 ± 11 | LC | NT | LC |
| *Cuculus fugax* | 60,595 | 10 | 4 | -18 | -39 | -48 | -78 | -1 ± 15 | -55 ± 21 | LC | NT | EN |
| *Cuculus micropterus* | 249,465 | 18 | 30 | 64 | 42 | 66 | 110 | 37 ± 24 | 73 ± 35 | LC | LC | LC |
| *Cuculus nisicolor* | 154,238 | -40 | -81 | -53 | -67 | -80 | -68 | -58 ± 21 | -72 ± 7 | LC | EN | EN |
| *Cuculus saturatus* | 278,831 | -19 | 4 | 11 | -22 | -18 | -62 | -1 ± 16 | -34 ± 24 | LC | NT | VU |
| *Cuculus sparverioides* | 223,369 | 5 | -16 | 47 | -50 | -56 | 1 | 12 ± 32 | -35 ± 31 | LC | LC | VU |
| *Cuculus vagans* | 24,937 | -4 | -11 | 26 | -45 | -21 | -56 | 4 ± 20 | -41 ± 18 | NT | LC | VU |
| *Cutia nipalensis* | 5,370 | -85 | -73 | 9 | -96 | -99 | -95 | -50 ± 51 | -97 ± 2 | VU | VU | CR |
| *Cyanoderma ambiguum* | 42,543 | -44 | -47 | -31 | -83 | -72 | -48 | -41 ± 9 | -68 ± 18 | LC | VU | EN |
| *Cyanoptila cumatilis* | 134,267 | 18 | -2 | 41 | -20 | 38 | 41 | 19 ± 21 | 20 ± 35 | LC | LC | LC |
| *Cyanoptila cyanomelana* | 196,067 | 39 | 5 | 22 | -55 | -37 | 29 | 22 ± 17 | -21 ± 44 | LC | LC | NT |
| *Cyornis banyumas* | 140,947 | -31 | -24 | -13 | -72 | -86 | -61 | -23 ± 9 | -73 ± 13 | LC | NT | EN |
| *Cyornis glaucicomans* | 158,097 | 15 | 24 | 94 | -13 | 79 | 101 | 45 ± 43 | 56 ± 61 | LC | LC | LC |
| *Cyornis hainanus* | 257,813 | 0 | -11 | -7 | -39 | -56 | -30 | -6 ± 6 | -41 ± 13 | LC | NT | VU |
| *Cyornis magnirostris* | 80,700 | -6 | -41 | -15 | -53 | -53 | -58 | -21 ± 18 | -54 ± 3 | LC | NT | EN |
| *Cyornis rubeculoides* | 149,415 | -47 | -66 | -34 | -73 | -90 | -89 | -49 ± 16 | -84 ± 9 | LC | VU | CR |
| *Cyornis tickelliae* | 213,739 | -42 | -65 | -41 | -79 | -55 | -48 | -49 ± 14 | -61 ± 16 | LC | VU | EN |
| *Cyornis unicolor* | 96,761 | -38 | -65 | -20 | -72 | -87 | -81 | -41 ± 23 | -80 ± 8 | LC | VU | EN |
| *Cypsiurus balasiensis* | 374,372 | 4 | 13 | 22 | 18 | -1 | 29 | 13 ± 9 | 15 ± 15 | LC | LC | LC |

| | | | | | | | | | | | | |
|---|---|---|---|---|---|---|---|---|---|---|---|---|
| *Delichon dasypus* | 148,473 | 12 | -34 | 58 | -45 | 119 | -37 | 12 ± 46 | 12 ± 92 | LC | LC | LC |
| *Delichon nipalense* | 4,313 | -64 | -100 | -98 | -99 | -100 | -100 | -87 ± 20 | -100 ± 0 | EN | CR | EX |
| *Delichon urbicum* | 36,656 | 275 | 78 | 385 | 21 | 527 | 662 | 246 ± 156 | 403 ± 338 | LC | LC | LC |
| *Dendrocitta formosae* | 57,328 | -43 | -71 | -18 | -70 | -94 | -85 | -44 ± 26 | -83 ± 12 | LC | VU | CR |
| *Dendrocitta vagabunda* | 125,887 | 4 | 53 | 104 | -23 | -54 | -16 | 53 ± 50 | -31 ± 20 | LC | LC | VU |
| *Dendrocopos atratus* | 44,135 | -29 | -24 | 40 | -49 | -74 | -48 | -4 ± 38 | -57 ± 15 | LC | NT | EN |
| *Dendrocopos canicapillus* | 219,325 | -34 | -49 | -24 | -53 | -86 | -81 | -36 ± 13 | -73 ± 18 | LC | VU | EN |
| *Dendrocopos cathpharius* | 12,532 | -74 | -82 | -76 | -92 | -100 | -100 | -77 ± 4 | -97 ± 5 | VU | EN | CR |
| *Dendrocopos macei* | 212,946 | -33 | -28 | -14 | -66 | -95 | -82 | -25 ± 10 | -81 ± 15 | LC | NT | CR |
| *Dendrocygna javanica* | 268,131 | 22 | 14 | 25 | -21 | -33 | 22 | 20 ± 6 | -11 ± 29 | LC | LC | NT |
| *Dendronanthus indicus* | 265,326 | -23 | -65 | -35 | -74 | -27 | -5 | -41 ± 22 | -35 ± 35 | LC | VU | VU |
| *Dicaeum agile* | 212,466 | -25 | -61 | -29 | -54 | 5 | -82 | -38 ± 20 | -44 ± 45 | LC | VU | VU |
| *Dicaeum chrysorrheum* | 187,764 | -44 | -76 | -41 | -75 | -96 | -93 | -54 ± 19 | -88 ± 11 | LC | EN | CR |
| *Dicaeum cruentatum* | 370,372 | -2 | -36 | 12 | 9 | -28 | 19 | -9 ± 25 | 0 ± 25 | LC | NT | LC |
| *Dicaeum ignipectus* | 64,935 | -54 | -88 | -60 | -83 | -97 | -95 | -67 ± 18 | -92 ± 8 | LC | EN | CR |
| *Dicaeum melanozanthum* | 7,828 | -65 | -96 | 85 | -95 | -99 | -93 | -25 ± 97 | -96 ± 3 | VU | NT | CR |
| *Dicaeum minullum* | 126,967 | -41 | -74 | -41 | -79 | -94 | -92 | -52 ± 19 | -88 ± 8 | LC | EN | CR |
| *Dicaeum trigonostigma* | 60,011 | -63 | -83 | -78 | -88 | -96 | -95 | -75 ± 10 | -93 ± 4 | LC | EN | CR |
| *Dicrurus aeneus* | 243,581 | -42 | -68 | -45 | -74 | -87 | -94 | -52 ± 14 | -85 ± 10 | LC | EN | CR |
| *Dicrurus annectens* | 216,674 | 13 | -39 | 49 | -36 | -1 | 19 | 8 ± 44 | -6 ± 28 | LC | LC | NT |
| *Dicrurus hottentottus* | 229,053 | -3 | -31 | 23 | -58 | -65 | -49 | -4 ± 27 | -57 ± 8 | LC | NT | EN |
| *Dicrurus leucophaeus* | 312,745 | -3 | -39 | 27 | -19 | -49 | 11 | -5 ± 33 | -19 ± 30 | LC | NT | NT |
| *Dicrurus macrocercus* | 364,571 | 5 | -1 | 22 | 9 | -22 | 23 | 9 ± 12 | 3 ± 23 | LC | LC | LC |
| *Dicrurus paradiseus* | 323,854 | -9 | -39 | 10 | -42 | -58 | -30 | -13 ± 25 | -43 ± 14 | LC | NT | VU |
| *Dicrurus remifer* | 145,315 | -22 | -31 | -17 | -44 | -61 | 8 | -23 ± 7 | -32 ± 36 | LC | NT | VU |
| *Dinopium javanense* | 168,104 | -41 | -53 | -37 | -60 | -75 | -94 | -43 ± 8 | -76 ± 17 | LC | VU | EN |
| *Dryocopus javensis* | 92,362 | -8 | 11 | 86 | 7 | 30 | 129 | 29 ± 50 | 55 ± 65 | LC | LC | LC |
| *Ducula aenea* | 89,730 | -24 | -29 | -9 | -45 | -81 | -61 | -21 ± 10 | -62 ± 18 | LC | NT | EN |
| *Ducula badia* | 78,285 | -46 | -80 | -67 | -79 | -99 | -94 | -64 ± 17 | -90 ± 11 | LC | EN | CR |
| *Ducula bicolor* | 26,464 | 26 | 167 | 178 | 36 | 190 | -49 | 124 ± 85 | 59 ± 121 | NT | LC | LC |
| *Dupetor flavicollis* | 223,693 | 7 | 26 | 0 | -29 | -7 | 40 | 11 ± 13 | 1 ± 36 | LC | LC | LC |
| *Egretta eulophotes* | 51,185 | 72 | 53 | 131 | 89 | 150 | -1 | 85 ± 41 | 79 ± 76 | LC | LC | LC |
| *Egretta garzetta* | 302,027 | 12 | 32 | 39 | 10 | -1 | 10 | 28 ± 14 | 6 ± 6 | LC | LC | LC |

| | | | | | | | | | | | | |
|---|---|---|---|---|---|---|---|---|---|---|---|---|
| *Egretta intermedia* | 226,787 | 22 | -16 | 23 | -56 | 2 | -68 | 9 ± 22 | -40 ± 37 | LC | LC | VU |
| *Egretta sacra* | 21,389 | 263 | 321 | 360 | 351 | 320 | 856 | 315 ± 49 | 509 ± 301 | NT | LC | LC |
| *Elanus caeruleus* | 304,116 | 21 | 36 | 41 | -47 | 43 | 70 | 33 ± 10 | 22 ± 61 | LC | LC | LC |
| *Enicurus immaculatus* | 20,648 | -35 | -62 | 19 | -80 | -84 | -96 | -26 ± 41 | -87 ± 8 | NT | NT | CR |
| *Enicurus leschenaulti* | 77,466 | -42 | -78 | -21 | -75 | -94 | -81 | -47 ± 29 | -83 ± 10 | LC | VU | CR |
| *Enicurus ruficapillus* | 36,072 | 14 | 1 | -31 | -40 | -16 | -68 | -5 ± 23 | -41 ± 26 | LC | NT | VU |
| *Enicurus schistaceus* | 77,731 | -45 | -77 | -31 | -73 | -84 | -90 | -51 ± 23 | -82 ± 9 | LC | EN | CR |
| *Erythrura prasina* | 104,701 | -55 | -90 | -64 | -94 | -99 | -99 | -70 ± 18 | -97 ± 3 | LC | EN | CR |
| *Eudynamys scolopaceus* | 307,881 | 3 | 21 | 29 | -12 | -14 | 20 | 18 ± 13 | -2 ± 19 | LC | LC | NT |
| *Eumyias thalassinus* | 182,791 | -35 | -63 | 8 | -61 | -75 | -65 | -30 ± 35 | -67 ± 7 | LC | VU | EN |
| *Eurystomus orientalis* | 237,760 | -29 | -72 | -42 | -82 | -85 | -97 | -48 ± 22 | -88 ± 8 | LC | VU | CR |
| *Falco amurensis* | 187,275 | -47 | -67 | -52 | -64 | -86 | -94 | -56 ± 10 | -81 ± 16 | LC | EN | CR |
| *Falco peregrinus* | 268,203 | 5 | -20 | 10 | -79 | 21 | 52 | -1 ± 16 | -2 ± 69 | LC | NT | NT |
| *Falco severus* | 190,280 | -18 | 33 | 8 | -53 | -68 | 35 | 8 ± 26 | -29 ± 55 | LC | LC | NT |
| *Falco subbuteo* | 155,804 | -19 | -25 | -7 | -63 | -55 | -44 | -17 ± 9 | -54 ± 9 | LC | NT | EN |
| *Falco tinnunculus* | 222,916 | 13 | -2 | 44 | -59 | -2 | 51 | 18 ± 23 | -3 ± 55 | LC | LC | NT |
| *Ficedula albicilla* | 364,214 | -1 | -16 | 11 | -5 | -33 | -10 | -2 ± 13 | -16 ± 15 | LC | NT | NT |
| *Ficedula elisae* | 88,338 | 45 | 79 | 56 | 55 | 182 | 49 | 60 ± 17 | 95 ± 75 | LC | LC | LC |
| *Ficedula erithacus* | 41,784 | -56 | -85 | -6 | -84 | -91 | -92 | -49 ± 40 | -89 ± 4 | LC | VU | CR |
| *Ficedula hyperythra* | 22,478 | -46 | -87 | 49 | -39 | -94 | -88 | -28 ± 70 | -74 ± 30 | NT | NT | EN |
| *Ficedula mugimaki* | 294,631 | -10 | -54 | -31 | -69 | -51 | -54 | -32 ± 22 | -58 ± 10 | LC | VU | EN |
| *Ficedula narcissina* | 177,237 | 103 | 144 | 150 | 156 | 175 | 190 | 132 ± 26 | 174 ± 17 | LC | LC | LC |
| *Ficedula sapphira* | 40,347 | -64 | -76 | -70 | -81 | -91 | -84 | -70 ± 6 | -85 ± 5 | LC | EN | CR |
| *Ficedula strophiata* | 19,829 | -63 | -91 | -51 | -94 | -99 | -98 | -68 ± 20 | -97 ± 3 | VU | EN | CR |
| *Ficedula superciliaris* | 29,856 | -40 | -66 | -74 | -94 | -88 | -97 | -60 ± 18 | -93 ± 5 | NT | EN | CR |
| *Ficedula tricolor* | 17,073 | -61 | -91 | -3 | -94 | -98 | -97 | -51 ± 45 | -97 ± 2 | VU | EN | CR |
| *Ficedula westermanni* | 45,646 | -47 | -71 | -5 | -67 | -85 | -85 | -41 ± 34 | -79 ± 10 | LC | VU | EN |
| *Ficedula zanthopygia* | 234,940 | 5 | -53 | -8 | -51 | 88 | 65 | -18 ± 30 | 34 ± 75 | LC | NT | LC |
| *Francolinus pintadeanus* | 103,820 | -11 | 5 | -2 | -69 | -77 | -76 | -3 ± 8 | -74 ± 5 | LC | NT | EN |
| *Fregata andrewsi* | 161,164 | 39 | 149 | 19 | -6 | 186 | 14 | 69 ± 70 | 65 ± 106 | LC | LC | LC |
| *Fregata ariel* | 36,587 | 78 | -64 | 14 | -68 | -100 | -66 | 9 ± 71 | -78 ± 19 | LC | LC | EN |
| *Fulica atra* | 81,125 | 95 | 215 | 217 | 11 | 46 | -81 | 176 ± 70 | -8 ± 65 | LC | LC | NT |
| *Gallicrex cinerea* | 175,334 | 12 | 12 | 50 | -65 | -71 | -91 | 25 ± 22 | -76 ± 14 | LC | LC | EN |

| | | | | | | | | | | | | |
|---|---|---|---|---|---|---|---|---|---|---|---|---|
| *Gallinago gallinago* | 184,616 | 24 | 43 | 59 | -40 | 103 | 81 | 42 ± 17 | 48 ± 77 | LC | LC | LC |
| *Gallinago stenura* | 165,267 | 34 | 58 | 70 | -54 | 29 | 136 | 54 ± 18 | 37 ± 95 | LC | LC | LC |
| *Gallinula chloropus* | 165,771 | 34 | 84 | 97 | 9 | 81 | -28 | 72 ± 33 | 21 ± 56 | LC | LC | LC |
| *Gallirallus striatus* | 105,382 | 12 | 19 | 66 | -20 | -68 | -99 | 32 ± 29 | -63 ± 40 | LC | LC | EN |
| *Gallus gallus* | 211,855 | -17 | -37 | -39 | -40 | -52 | -42 | -31 ± 12 | -45 ± 7 | LC | VU | VU |
| *Gampsorhynchus torquatus* | 52,308 | -64 | -89 | -60 | -90 | -100 | -98 | -71 ± 16 | -96 ± 5 | LC | EN | CR |
| *Garrulax chinensis* | 34,026 | -75 | -99 | -92 | -99 | -100 | -100 | -88 ± 12 | -100 ± 1 | LC | CR | EX |
| *Garrulax leucolophus* | 155,147 | 0 | -17 | 22 | -42 | -34 | 24 | 2 ± 20 | -17 ± 36 | LC | LC | NT |
| *Garrulax monileger* | 111,523 | -52 | -65 | -66 | -86 | -98 | -100 | -61 ± 8 | -95 ± 8 | LC | EN | CR |
| *Garrulax pectoralis* | 24,907 | -65 | -90 | -97 | -89 | -75 | -100 | -84 ± 17 | -88 ± 12 | NT | CR | CR |
| *Garrulax strepitans* | 36,045 | -42 | -57 | 65 | -80 | -99 | -45 | -11 ± 67 | -75 ± 27 | LC | NT | EN |
| *Garrulus glandarius* | 124,033 | -17 | -10 | 11 | -26 | -55 | -64 | -5 ± 15 | -48 ± 20 | LC | NT | VU |
| *Gecinulus grantia* | 130,358 | -34 | -64 | -24 | -63 | -90 | -54 | -41 ± 21 | -69 ± 19 | LC | VU | EN |
| *Gelochelidon nilotica* | 12,763 | 266 | -50 | 901 | 9 | 144 | -97 | 372 ± 485 | 19 ± 121 | VU | LC | LC |
| *Glareola lactea* | 127,016 | 31 | 133 | 50 | 131 | 80 | -76 | 71 ± 54 | 45 ± 108 | LC | LC | LC |
| *Glareola maldivarum* | 157,725 | 47 | 108 | 103 | 73 | 153 | 168 | 86 ± 34 | 132 ± 51 | LC | LC | LC |
| *Glaucidium brodiei* | 106,313 | -42 | -88 | -68 | -78 | -97 | -99 | -66 ± 23 | -91 ± 12 | LC | EN | CR |
| *Glaucidium cuculoides* | 284,862 | -1 | -1 | 21 | -53 | -55 | 39 | 6 ± 13 | -23 ± 54 | LC | LC | NT |
| *Gorsachius melanolophus* | 237,109 | 19 | 69 | 92 | 69 | 40 | 91 | 60 ± 37 | 67 ± 25 | LC | LC | LC |
| *Gracula religiosa* | 143,614 | -36 | -82 | -46 | -82 | -73 | -95 | -55 ± 24 | -83 ± 11 | LC | EN | CR |
| *Gracupica contra* | 145,688 | 11 | -30 | 38 | -46 | -14 | -16 | 6 ± 34 | -25 ± 18 | LC | LC | NT |
| *Gracupica nigricollis* | 230,685 | -25 | -70 | -52 | -80 | -83 | -64 | -49 ± 23 | -76 ± 11 | LC | VU | EN |
| *Grus antigone* | 13,283 | 166 | -98 | -37 | -99 | -100 | 164 | 10 ± 138 | -12 ± 152 | VU | LC | NT |
| *Gyps himalayensis* | 311,984 | -3 | -19 | 9 | 2 | -52 | 13 | -4 ± 14 | -12 ± 35 | LC | NT | NT |
| *Halcyon coromanda* | 107,838 | 9 | 67 | -22 | -6 | 111 | -50 | 18 ± 45 | 18 ± 83 | LC | LC | LC |
| *Halcyon pileata* | 283,549 | 4 | -2 | -8 | -17 | -14 | 53 | -2 ± 6 | 7 ± 40 | LC | NT | LC |
| *Halcyon smyrnensis* | 331,377 | 1 | 10 | 29 | 6 | -28 | 14 | 13 ± 14 | -3 ± 23 | LC | LC | NT |
| *Haliaeetus leucogaster* | 36,874 | 41 | 7 | -12 | 34 | -59 | -82 | 12 ± 27 | -35 ± 61 | LC | LC | VU |
| *Haliastur indus* | 120,627 | -3 | -29 | 47 | -27 | -49 | 66 | 5 ± 39 | -3 ± 61 | LC | LC | NT |
| *Hemicircus canente* | 142,272 | -50 | -93 | -92 | -91 | -99 | -100 | -78 ± 25 | -97 ± 5 | LC | EN | CR |
| *Hemicircus concretus* | 21,694 | -13 | -32 | -10 | -41 | -80 | -73 | -18 ± 12 | -65 ± 21 | NT | NT | EN |
| *Hemiprocne comata* | 32,561 | -41 | -69 | -49 | -73 | -93 | -88 | -53 ± 14 | -85 ± 11 | LC | EN | CR |
| *Hemiprocne coronata* | 140,668 | -42 | -42 | -36 | -76 | -89 | -79 | -40 ± 4 | -81 ± 7 | LC | VU | CR |

| | | | | | | | | | | | | |
|---|---|---|---|---|---|---|---|---|---|---|---|---|
| *Hemiprocne longipennis* | 33,335 | -43 | -69 | -63 | -75 | -97 | -88 | -58 ± 14 | -87 ± 11 | LC | EN | CR |
| *Hemixos cinereus* | 40,817 | -7 | -17 | 0 | -68 | -57 | 6 | -8 ± 9 | -40 ± 40 | LC | NT | VU |
| *Hemixos flavala* | 74,403 | -50 | -74 | -24 | -80 | -93 | -90 | -49 ± 25 | -88 ± 7 | LC | VU | CR |
| *Heterophasia annectans* | 37,048 | -33 | 33 | 53 | 269 | -26 | -100 | 18 ± 45 | 48 ± 195 | LC | LC | LC |
| *Heterophasia melanoleuca* | 29,258 | -36 | -71 | 68 | -73 | -85 | -81 | -13 ± 72 | -80 ± 6 | NT | NT | EN |
| *Heterophasia picaoides* | 45,870 | -46 | -53 | 51 | -74 | -96 | -62 | -16 ± 58 | -77 ± 18 | LC | NT | EN |
| *Hieraaetus pennatus* | 210,890 | -39 | -26 | 48 | -45 | -74 | -31 | -6 ± 47 | -50 ± 22 | LC | NT | VU |
| *Himantopus himantopus* | 162,228 | 50 | 46 | 68 | -41 | -57 | 71 | 54 ± 12 | -9 ± 70 | LC | LC | NT |
| *Hirundapus caudacutus* | 187,269 | -24 | -75 | -44 | -49 | -59 | -77 | -48 ± 25 | -62 ± 14 | LC | VU | EN |
| *Hirundapus cochinchinensis* | 48,550 | -36 | -40 | -14 | -29 | -79 | 64 | -30 ± 14 | -15 ± 73 | LC | VU | NT |
| *Hirundapus giganteus* | 192,913 | -42 | -78 | -52 | -74 | -82 | -95 | -57 ± 18 | -84 ± 11 | LC | EN | CR |
| *Hirundo rustica* | 378,641 | 4 | 4 | 27 | 18 | -8 | 16 | 12 ± 13 | 9 ± 14 | LC | LC | LC |
| *Hirundo smithii* | 22,623 | -47 | 65 | 39 | -43 | -59 | -83 | 19 ± 59 | -62 ± 20 | NT | LC | EN |
| *Hirundo tahitica* | 54,339 | 67 | 48 | 102 | 91 | 99 | 41 | 73 ± 27 | 77 ± 31 | LC | LC | LC |
| *Hodgsonius phoenicuroides* | 15,260 | -65 | -69 | -29 | -83 | -100 | -97 | -54 ± 22 | -93 ± 9 | VU | EN | CR |
| *Hydrophasianus chirurgus* | 112,574 | -12 | 51 | 42 | -64 | -48 | -64 | 27 ± 34 | -58 ± 9 | LC | LC | EN |
| *Hydroprogne caspia* | 13,212 | 226 | 175 | 243 | 196 | 708 | 410 | 215 ± 35 | 438 ± 257 | VU | LC | LC |
| *Hypogramma hypogrammicum* | 126,743 | -31 | -59 | -54 | -70 | -42 | -90 | -48 ± 15 | -68 ± 24 | LC | VU | EN |
| *Hypothymis azurea* | 315,934 | 8 | -12 | 13 | -34 | -44 | -43 | 3 ± 13 | -40 ± 6 | LC | LC | VU |
| *Hypsipetes leucocephalus* | 69,372 | -46 | -69 | -34 | -76 | -93 | -90 | -49 ± 18 | -86 ± 9 | LC | VU | CR |
| *Ianthocincla pectoralis* | 78,645 | -49 | -59 | -32 | -76 | -88 | -97 | -47 ± 14 | -87 ± 10 | LC | VU | CR |
| *Ianthocincla sannio* | 2,178 | -91 | -93 | -51 | -96 | -99 | -98 | -78 ± 24 | -98 ± 2 | EN | EN | CR |
| *Ichthyaetus ichthyaetus* | 358,951 | 38 | 41 | 25 | 51 | -3 | 60 | 35 ± 8 | 36 ± 34 | LC | LC | LC |
| *Icthyophaga humilis* | 38,245 | -39 | -55 | 126 | -23 | -90 | -77 | 11 ± 100 | -64 ± 35 | LC | LC | EN |
| *Icthyophaga ichthyaetus* | 42,749 | 28 | 61 | 68 | 20 | -4 | 261 | 52 ± 22 | 92 ± 147 | LC | LC | LC |
| *Ictinaetus malayensis* | 127,136 | -50 | -87 | -63 | -91 | -99 | -98 | -67 ± 19 | -96 ± 5 | LC | EN | CR |
| *Iole crypta* | 24,988 | 41 | 53 | 38 | 6 | -31 | -69 | 44 ± 8 | -31 ± 38 | NT | LC | VU |
| *Iole propinqua* | 86,334 | -28 | -58 | -43 | -68 | -76 | -91 | -43 ± 15 | -78 ± 12 | LC | VU | EN |
| *Iole viridescens* | 75,395 | -36 | -49 | -13 | -38 | -79 | -67 | -33 ± 18 | -61 ± 21 | LC | VU | EN |
| *Ixobrychus cinnamomeus* | 224,357 | 23 | 52 | 63 | -37 | 59 | 82 | 46 ± 21 | 35 ± 63 | LC | LC | LC |
| *Ixobrychus sinensis* | 190,981 | 27 | 43 | 57 | -47 | 10 | -99 | 42 ± 15 | -45 ± 54 | LC | LC | VU |
| *Ixos malaccensis* | 32,920 | -19 | -15 | -9 | -28 | -45 | 9 | -15 ± 5 | -21 ± 27 | LC | NT | NT |
| *Ixos mcclellandii* | 75,777 | -46 | -74 | -20 | -74 | -91 | -88 | -47 ± 27 | -84 ± 9 | LC | VU | CR |

| | | | | | | | | | | | | |
|---|---|---|---|---|---|---|---|---|---|---|---|---|
| *Jynx torquilla* | 115,006 | -39 | -4 | 36 | -57 | -45 | -94 | -2 ± 38 | -65 ± 25 | LC | NT | EN |
| *Ketupa ketupu* | 29,516 | 11 | 15 | -46 | -65 | -63 | -75 | -7 ± 34 | -68 ± 6 | NT | NT | EN |
| *Ketupa zeylonensis* | 123,094 | -12 | -34 | 13 | 16 | -89 | 51 | -11 ± 23 | -8 ± 73 | LC | NT | NT |
| *Lacedo pulchella* | 81,763 | -53 | -70 | -71 | -86 | -98 | -98 | -65 ± 10 | -94 ± 7 | LC | EN | CR |
| *Lalage nigra* | 41,034 | 47 | 58 | 45 | 0 | 43 | -50 | 50 ± 7 | -2 ± 47 | LC | LC | NT |
| *Lanius collurioides* | 188,939 | 24 | -27 | 34 | -46 | -60 | -1 | 10 ± 32 | -36 ± 31 | LC | LC | VU |
| *Lanius cristatus* | 377,280 | 5 | -10 | 17 | -4 | -39 | 18 | 4 ± 13 | -8 ± 29 | LC | LC | NT |
| *Lanius schach* | 155,896 | 6 | -71 | 44 | -53 | -33 | 22 | -7 ± 59 | -21 ± 39 | LC | NT | NT |
| *Lanius tephronotus* | 77,944 | -38 | -58 | -18 | -69 | -88 | -81 | -38 ± 20 | -79 ± 10 | LC | VU | EN |
| *Lanius tigrinus* | 257,264 | -4 | -44 | 53 | -44 | 11 | -1 | 2 ± 48 | -11 ± 29 | LC | LC | NT |
| *Larus crassirostris* | 1,473 | -22 | -20 | -26 | -24 | 22 | -55 | -23 ± 3 | -19 ± 39 | EN | NT | NT |
| *Larus fuscus* | 23,986 | -10 | -89 | 14 | -54 | -24 | 31 | -28 ± 53 | -16 ± 43 | NT | NT | NT |
| *Leiothrix argentauris* | 28,752 | -42 | -57 | 28 | -79 | -92 | -67 | -24 ± 46 | -79 ± 12 | NT | NT | EN |
| *Leptocoma brasiliana* | 77,731 | 40 | -20 | 37 | 4 | 52 | 39 | 19 ± 34 | 32 ± 25 | LC | LC | LC |
| *Leptocoma calcostetha* | 42,575 | 56 | -18 | 74 | -63 | -21 | -49 | 37 ± 49 | -44 ± 21 | LC | LC | VU |
| *Leptoptilos javanicus* | 26,222 | 423 | 772 | 974 | 1126 | 1487 | 1696 | 723 ± 279 | 1436 ± 288 | NT | LC | LC |
| *Limicola falcinellus* | 11,909 | -26 | -48 | 11 | -83 | 274 | -76 | -21 ± 30 | 38 ± 205 | VU | NT | LC |
| *Limnodromus semipalmatus* | 2,196 | -43 | -95 | -84 | -93 | -77 | -100 | -74 ± 28 | -90 ± 12 | EN | EN | CR |
| *Limosa lapponica* | 35,920 | -5 | 52 | 50 | -27 | 75 | -99 | 32 ± 32 | -17 ± 87 | LC | LC | NT |
| *Limosa limosa* | 60,990 | 180 | 181 | 362 | -20 | 413 | -85 | 241 ± 105 | 102 ± 271 | LC | LC | LC |
| *Liocichla ripponi* | 2,294 | -92 | -93 | -60 | -94 | -100 | -100 | -82 ± 19 | -98 ± 3 | EN | CR | CR |
| *Lonchura atricapilla* | 97,710 | 72 | 115 | 58 | 208 | 361 | 51 | 82 ± 30 | 206 ± 155 | LC | LC | LC |
| *Lonchura leucogastra* | 19,795 | 99 | 184 | 221 | 140 | 155 | 216 | 168 ± 63 | 170 ± 40 | VU | LC | LC |
| *Lonchura maja* | 67,945 | 7 | 26 | 40 | 11 | -64 | 76 | 24 ± 16 | 8 ± 70 | LC | LC | LC |
| *Lonchura punctulata* | 345,596 | 3 | 5 | 28 | 22 | -2 | 25 | 12 ± 14 | 15 ± 15 | LC | LC | LC |
| *Lonchura striata* | 382,106 | -18 | -56 | -13 | -61 | -63 | -46 | -29 ± 23 | -57 ± 9 | LC | NT | EN |
| *Lophotriorchis kienerii* | 135,645 | -52 | -82 | -55 | -82 | -96 | -95 | -63 ± 17 | -91 ± 8 | LC | EN | CR |
| *Lophura diardi* | 101,639 | -26 | 12 | -58 | -1 | 17 | -62 | -24 ± 35 | -15 ± 41 | LC | NT | NT |
| *Lophura leucomelanos* | 33,239 | -55 | -63 | -69 | -85 | -95 | -100 | -62 ± 7 | -93 ± 8 | LC | EN | CR |
| *Lophura nycthemera* | 82,014 | -50 | -81 | -77 | -83 | -95 | -99 | -69 ± 17 | -92 ± 8 | LC | EN | CR |
| *Loriculus galgulus* | 10,802 | 42 | 147 | 129 | 68 | -16 | 7 | 106 ± 56 | 20 ± 43 | VU | LC | LC |
| *Loriculus vernalis* | 166,556 | -51 | -70 | -60 | -77 | -95 | -97 | -60 ± 10 | -90 ± 11 | LC | EN | CR |
| *Luscinia calliope* | 145,673 | 33 | 89 | 116 | 103 | 103 | 143 | 79 ± 42 | 116 ± 23 | LC | LC | LC |

| | | | | | | | | | | | | |
|---|---|---|---|---|---|---|---|---|---|---|---|---|
| *Luscinia cyane* | 226,482 | -14 | -57 | 5 | -57 | -57 | -72 | -22 ± 31 | -62 ± 9 | LC | NT | EN |
| *Luscinia sibilans* | 203,332 | -29 | -74 | -15 | -65 | -93 | -88 | -39 ± 31 | -82 ± 15 | LC | VU | CR |
| *Luscinia svecica* | 87,885 | -53 | -31 | 123 | -53 | -57 | -30 | 13 ± 96 | -47 ± 15 | LC | LC | VU |
| *Lyncornis macrotis* | 99,901 | -60 | -93 | -87 | -93 | -92 | -100 | -80 ± 17 | -95 ± 4 | LC | EN | CR |
| *Macheiramphus alcinus* | 40,671 | -13 | -53 | -42 | -45 | -93 | -88 | -36 ± 20 | -75 ± 26 | LC | VU | EN |
| *Macronus gularis* | 271,918 | -9 | -35 | 8 | -44 | -46 | -41 | -12 ± 22 | -44 ± 3 | LC | NT | VU |
| *Macronus ptilosus* | 19,741 | 17 | 67 | -13 | 21 | 45 | -39 | 24 ± 41 | 9 ± 43 | VU | LC | LC |
| *Macropygia ruficeps* | 87,861 | -53 | -83 | -59 | -91 | -96 | -99 | -65 ± 16 | -95 ± 4 | LC | EN | CR |
| *Macropygia unchall* | 79,715 | -40 | -5 | -32 | -83 | -94 | -60 | -26 ± 18 | -79 ± 17 | LC | NT | EN |
| *Malacocincla abbotti* | 46,425 | -20 | -67 | -75 | -76 | -88 | -92 | -54 ± 29 | -86 ± 8 | LC | EN | CR |
| *Malacopteron cinereum* | 37,566 | -5 | -5 | -13 | -8 | 24 | -36 | -8 ± 5 | -7 ± 30 | LC | NT | NT |
| *Malacopteron magnirostre* | 22,060 | 102 | 109 | 66 | 41 | 69 | -2 | 92 ± 23 | 36 ± 36 | NT | LC | LC |
| *Malacopteron magnum* | 15,472 | 80 | 127 | 114 | 92 | 130 | 70 | 107 ± 24 | 97 ± 30 | VU | LC | LC |
| *Megaceryle lugubris* | 123,941 | -34 | -38 | 28 | -52 | -92 | -30 | -14 ± 37 | -58 ± 31 | LC | NT | EN |
| *Megalaima asiatica* | 94,362 | -50 | -74 | -42 | -78 | -94 | -93 | -55 ± 17 | -88 ± 9 | LC | EN | CR |
| *Megalaima australis* | 165,221 | -32 | -42 | -16 | -53 | -69 | -92 | -30 ± 13 | -71 ± 20 | LC | VU | EN |
| *Megalaima chrysopogon* | 30,825 | -9 | -24 | -21 | -33 | -49 | -57 | -18 ± 8 | -46 ± 12 | LC | NT | VU |
| *Megalaima faiostricta* | 130,731 | -44 | -85 | -52 | -88 | -98 | -98 | -60 ± 22 | -94 ± 6 | LC | EN | CR |
| *Megalaima franklinii* | 63,766 | -56 | -83 | -30 | -86 | -96 | -93 | -56 ± 27 | -91 ± 5 | LC | EN | CR |
| *Megalaima haemacephala* | 342,497 | 0 | 8 | 15 | -3 | -40 | 9 | 8 ± 8 | -11 ± 26 | LC | LC | NT |
| *Megalaima henricii* | 16,725 | 54 | 48 | 200 | 197 | -29 | 170 | 101 ± 86 | 112 ± 123 | VU | LC | LC |
| *Megalaima incognita* | 40,730 | -68 | -93 | -95 | -95 | -100 | -100 | -85 ± 15 | -98 ± 3 | LC | CR | CR |
| *Megalaima lineata* | 291,259 | 23 | 36 | 25 | -7 | -11 | -31 | 28 ± 7 | -16 ± 13 | LC | LC | NT |
| *Megalaima mystacophanos* | 43,614 | -46 | -50 | -43 | -67 | -97 | -78 | -46 ± 3 | -81 ± 15 | LC | VU | CR |
| *Megalaima rafflesii* | 42,270 | 40 | 73 | 77 | 228 | 178 | 232 | 63 ± 21 | 213 ± 30 | LC | LC | LC |
| *Megalaima virens* | 78,012 | -51 | -76 | -37 | -83 | -94 | -94 | -55 ± 20 | -90 ± 6 | LC | EN | CR |
| *Megapomatorhinus erythrogenys* | 18,685 | -70 | -88 | -55 | -80 | -99 | -99 | -71 ± 16 | -92 ± 11 | VU | EN | CR |
| *Megapomatorhinus hypoleucos* | 117,552 | -51 | -90 | -83 | -85 | -98 | -100 | -75 ± 21 | -94 ± 8 | LC | EN | CR |
| *Meiglyptes jugularis* | 17,491 | -74 | -90 | -95 | -94 | -100 | -100 | -86 ± 11 | -98 ± 3 | VU | CR | CR |
| *Meiglyptes tristis* | 23,160 | -28 | -31 | -2 | -48 | -64 | -59 | -20 ± 16 | -57 ± 9 | NT | NT | EN |
| *Meiglyptes tukki* | 24,775 | -1 | -5 | 25 | 65 | -83 | 18 | 6 ± 16 | 0 ± 76 | NT | LC | LC |
| *Merops leschenaulti* | 293,543 | -5 | -35 | 13 | -45 | -58 | -83 | -9 ± 24 | -62 ± 19 | LC | NT | EN |
| *Merops orientalis* | 291,624 | -7 | -17 | -37 | -87 | -76 | -37 | -20 ± 15 | -66 ± 26 | LC | NT | EN |

| | | | | | | | | | | | | |
|---|---|---|---|---|---|---|---|---|---|---|---|---|
| *Merops philippinus* | 161,008 | 43 | 50 | 76 | -5 | 36 | 118 | 56 ± 17 | 50 ± 63 | LC | LC | LC |
| *Merops viridis* | 143,095 | -10 | -8 | 20 | -37 | -15 | -47 | 1 ± 17 | -33 ± 17 | LC | LC | VU |
| *Metopidius indicus* | 121,041 | -1 | -31 | -8 | -78 | -13 | -65 | -13 ± 16 | -52 ± 34 | LC | NT | EN |
| *Microcarbo niger* | 184,717 | 35 | 31 | 87 | -28 | 20 | 110 | 51 ± 31 | 34 ± 70 | LC | LC | LC |
| *Microhierax caerulescens* | 64,672 | -53 | -33 | -12 | -63 | -93 | -81 | -32 ± 20 | -79 ± 15 | LC | VU | EN |
| *Microhierax fringillarius* | 22,663 | -31 | -44 | -32 | -40 | -97 | -87 | -36 ± 8 | -75 ± 30 | NT | VU | EN |
| *Milvus migrans* | 210,941 | 34 | 50 | 43 | -33 | 63 | 126 | 42 ± 8 | 52 ± 80 | LC | LC | LC |
| *Minla annectens* | 25,443 | -46 | -67 | 45 | -71 | -100 | -70 | -23 ± 60 | -80 ± 17 | NT | NT | EN |
| *Minla cyanouroptera* | 35,316 | -39 | -59 | -30 | -74 | -89 | -77 | -43 ± 15 | -80 ± 8 | LC | VU | EN |
| *Minla strigula* | 12,212 | -45 | -97 | -91 | -99 | -100 | -100 | -78 ± 29 | -100 ± 0 | VU | EN | EX |
| *Monticola gularis* | 177,421 | -29 | -62 | 2 | -58 | -70 | -37 | -29 ± 32 | -55 ± 16 | LC | NT | EN |
| *Monticola rufiventris* | 26,353 | -66 | -76 | -14 | -87 | -96 | -96 | -52 ± 33 | -93 ± 5 | NT | EN | CR |
| *Monticola solitarius* | 283,459 | 17 | -35 | -2 | -31 | -7 | -1 | -7 ± 26 | -13 ± 16 | LC | NT | NT |
| *Motacilla alba* | 232,849 | -8 | -1 | 51 | 8 | -48 | 43 | 14 ± 32 | 1 ± 46 | LC | LC | LC |
| *Motacilla cinerea* | 253,604 | -25 | -38 | 44 | -47 | -31 | 43 | -6 ± 44 | -12 ± 48 | LC | NT | NT |
| *Motacilla citreola* | 76,510 | 31 | 222 | 210 | 165 | 226 | 339 | 154 ± 107 | 243 ± 88 | LC | LC | LC |
| *Motacilla tschutschensis* | 272,731 | -8 | 12 | 49 | 34 | 59 | 62 | 18 ± 29 | 52 ± 15 | LC | LC | LC |
| *Mulleripicus pulverulentus* | 90,311 | -39 | -55 | -47 | -75 | -91 | -94 | -47 ± 8 | -87 ± 10 | LC | VU | CR |
| *Muscicapa ferruginea* | 198,273 | 48 | 48 | 71 | 60 | 78 | 88 | 56 ± 13 | 75 ± 14 | LC | LC | LC |
| *Muscicapa latirostris* | 344,516 | -7 | -30 | 25 | -20 | -7 | 25 | -4 ± 28 | 0 ± 23 | LC | NT | LC |
| *Muscicapa sibirica* | 219,444 | -18 | -40 | 19 | -24 | 10 | 6 | -13 ± 30 | -2 ± 19 | LC | NT | NT |
| *Muscicapa williamsoni* | 33,530 | 60 | 71 | 48 | 70 | -13 | -52 | 60 ± 11 | 2 ± 62 | LC | LC | LC |
| *Mycteria cinerea* | 5,584 | -25 | 370 | 1241 | 975 | 170 | -73 | 529 ± 648 | 357 ± 548 | VU | LC | LC |
| *Mycteria leucocephala* | 48,290 | 97 | 161 | 182 | -76 | 41 | 331 | 147 ± 44 | 99 ± 210 | LC | LC | LC |
| *Myiomela leucura* | 74,013 | -31 | -47 | 86 | -84 | -74 | 42 | 3 ± 73 | -39 ± 70 | LC | LC | VU |
| *Myophonus caeruleus* | 211,850 | -17 | -52 | -22 | -55 | -67 | -45 | -31 ± 19 | -56 ± 11 | LC | VU | EN |
| *Napothera brevicaudata* | 137,617 | -27 | -60 | -14 | -70 | -88 | -62 | -34 ± 24 | -73 ± 13 | LC | VU | EN |
| *Napothera crispifrons* | 50,211 | -23 | 53 | 20 | -16 | -32 | -39 | 17 ± 38 | -29 ± 12 | LC | LC | NT |
| *Napothera epilepidota* | 48,243 | -44 | -77 | -56 | -96 | -95 | -88 | -59 ± 17 | -93 ± 5 | LC | EN | CR |
| *Napothera macrodactyla* | 29,376 | 64 | -31 | 77 | -41 | 34 | 376 | 37 ± 59 | 123 ± 222 | NT | LC | LC |
| *Netta rufina* | 148,823 | 34 | 18 | -9 | 19 | -22 | 12 | 14 ± 22 | 3 ± 22 | LC | LC | LC |
| *Nettapus coromandelianus* | 211,880 | 16 | 53 | 63 | -29 | 92 | -97 | 44 ± 25 | -11 ± 96 | LC | LC | NT |
| *Niltava grandis* | 36,519 | -57 | -70 | -41 | -80 | -89 | -87 | -56 ± 15 | -85 ± 5 | LC | EN | CR |

| | | | | | | | | | | | | |
|---|---|---|---|---|---|---|---|---|---|---|---|---|
| *Niltava macgrigoriae* | 47,096 | -35 | -58 | 42 | -78 | -96 | -82 | -17 ± 53 | -85 ± 9 | LC | NT | CR |
| *Niltava sundara* | 45,352 | -21 | -33 | 127 | -22 | -74 | -35 | 24 ± 89 | -44 ± 27 | LC | LC | VU |
| *Niltava vivida* | 38,042 | -50 | -88 | -25 | -92 | -99 | -96 | -54 ± 32 | -96 ± 3 | LC | EN | CR |
| *Ninox japonica* | 194,374 | 108 | 141 | 136 | 106 | 53 | 63 | 128 ± 18 | 74 ± 28 | LC | LC | LC |
| *Ninox scutulata* | 160,211 | -28 | -63 | -40 | -63 | -66 | -83 | -44 ± 18 | -71 ± 11 | LC | VU | EN |
| *Nisaetus alboniger* | 33,438 | 7 | 32 | 6 | -33 | -48 | -64 | 15 ± 15 | -48 ± 15 | LC | LC | VU |
| *Nisaetus cirrhatus* | 155,382 | -46 | -60 | -46 | -75 | -97 | -87 | -51 ± 8 | -86 ± 11 | LC | EN | CR |
| *Nisaetus nanus* | 15,896 | 1 | -22 | -8 | -32 | -75 | -76 | -10 ± 11 | -61 ± 25 | VU | NT | EN |
| *Nisaetus nipalensis* | 78,679 | -50 | -87 | -62 | -92 | -99 | -99 | -66 ± 19 | -97 ± 4 | LC | EN | CR |
| *Numenius arquata* | 100,078 | 28 | -41 | -3 | -76 | 15 | -87 | -5 ± 34 | -50 ± 56 | LC | NT | VU |
| *Numenius madagascariensis* | 193,113 | 86 | 6 | 116 | 165 | 87 | 110 | 69 ± 57 | 121 ± 40 | LC | LC | LC |
| *Numenius phaeopus* | 21,834 | 298 | 243 | 428 | 529 | 1122 | -50 | 323 ± 95 | 534 ± 586 | NT | LC | LC |
| *Nycticorax nycticorax* | 183,464 | 25 | 37 | 36 | -42 | -11 | 111 | 33 ± 7 | 19 ± 81 | LC | LC | LC |
| *Nyctyornis amictus* | 35,556 | -45 | -77 | -59 | -81 | -99 | -94 | -60 ± 16 | -91 ± 9 | LC | EN | CR |
| *Nyctyornis athertoni* | 129,522 | -55 | -85 | -46 | -88 | -98 | -98 | -62 ± 20 | -95 ± 5 | LC | EN | CR |
| *Onychoprion anaethetus* | 36,900 | 51 | 214 | 21 | 172 | 199 | -30 | 95 ± 104 | 113 ± 125 | LC | LC | LC |
| *Orthotomus atrogularis* | 339,780 | -5 | -46 | 15 | -44 | -40 | -26 | -12 ± 31 | -37 ± 10 | LC | NT | VU |
| *Orthotomus ruficeps* | 28,022 | 8 | 8 | 110 | -27 | -67 | -73 | 42 ± 59 | -56 ± 25 | NT | LC | EN |
| *Orthotomus sericeus* | 38,181 | 22 | 49 | -17 | -55 | -31 | -39 | 18 ± 33 | -42 ± 12 | LC | LC | VU |
| *Orthotomus sutorius* | 418,380 | 0 | -12 | 13 | -1 | -30 | 15 | 1 ± 12 | -5 ± 23 | LC | LC | NT |
| *Otus lempiji* | 31,364 | 35 | -93 | -4 | -49 | -82 | -76 | -21 ± 66 | -69 ± 18 | LC | NT | EN |
| *Otus lettia* | 228,774 | -33 | -29 | 6 | -62 | -79 | -95 | -19 ± 21 | -78 ± 17 | LC | NT | EN |
| *Otus sagittatus* | 46,001 | -47 | -90 | -75 | -97 | -90 | -87 | -71 ± 22 | -91 ± 5 | LC | EN | CR |
| *Otus spilocephalus* | 68,883 | -48 | -90 | -49 | -86 | -99 | -97 | -62 ± 24 | -94 ± 7 | LC | EN | CR |
| *Otus sunia* | 153,413 | -2 | 19 | 78 | 19 | -13 | 31 | 31 ± 41 | 12 ± 23 | LC | LC | LC |
| *Pandion haliaetus* | 200,582 | -5 | -21 | 40 | -66 | 56 | 140 | 5 ± 32 | 43 ± 104 | LC | LC | LC |
| *Passer flaveolus* | 266,535 | 13 | -39 | 0 | 14 | 4 | 28 | -9 ± 27 | 15 ± 12 | LC | NT | LC |
| *Passer montanus* | 344,635 | 8 | 8 | 30 | 29 | -9 | 31 | 15 ± 13 | 17 ± 22 | LC | LC | LC |
| *Pastor roseus* | 59,243 | -3 | 143 | 187 | 109 | -27 | 62 | 109 ± 100 | 48 ± 69 | LC | LC | LC |
| *Pavo muticus* | 76,950 | -41 | 8 | 16 | -30 | -90 | -69 | -6 ± 31 | -63 ± 30 | LC | NT | EN |
| *Pelargopsis amauroptera* | 7,947 | 47 | 84 | -20 | 85 | 14 | -29 | 37 ± 53 | 23 ± 58 | VU | LC | LC |
| *Pelargopsis capensis* | 138,708 | -12 | -7 | -20 | -72 | -69 | -90 | -13 ± 7 | -77 ± 12 | LC | NT | EN |
| *Pelecanus philippensis* | 23,265 | -60 | -90 | 135 | -89 | -50 | 26 | -5 ± 122 | -38 ± 58 | NT | NT | VU |

| | | | | | | | | | | | | |
|---|---|---|---|---|---|---|---|---|---|---|---|---|
| *Pellorneum albiventre* | 3,706 | -80 | -99 | -39 | -99 | -100 | -99 | -73 ± 30 | -99 ± 1 | EN | EN | CR |
| *Pellorneum capistratum* | 23,347 | 45 | 30 | 22 | 27 | 35 | -57 | 32 ± 12 | 2 ± 51 | NT | LC | LC |
| *Pellorneum ruficeps* | 238,139 | -20 | -58 | -8 | -47 | -69 | -80 | -28 ± 26 | -65 ± 17 | LC | NT | EN |
| *Pellorneum tickelli* | 105,948 | -45 | -81 | -41 | -78 | -96 | -75 | -56 ± 22 | -83 ± 11 | LC | EN | CR |
| *Pericrocotus brevirostris* | 64,837 | -41 | -62 | 21 | -74 | -74 | -84 | -28 ± 43 | -77 ± 6 | LC | NT | EN |
| *Pericrocotus cantonensis* | 172,888 | -14 | -14 | 48 | -26 | -42 | -10 | 7 ± 36 | -26 ± 16 | LC | LC | NT |
| *Pericrocotus cinnamomeus* | 115,398 | -15 | -25 | 23 | -35 | -58 | -56 | -5 ± 25 | -50 ± 13 | LC | NT | VU |
| *Pericrocotus divaricatus* | 265,132 | -14 | -69 | 2 | -39 | -70 | -25 | -27 ± 37 | -44 ± 23 | LC | NT | VU |
| *Pericrocotus ethologus* | 33,564 | -62 | -54 | -14 | -59 | -75 | -67 | -43 ± 26 | -67 ± 8 | LC | VU | EN |
| *Pericrocotus igneus* | 39,360 | 8 | -21 | -25 | -68 | -82 | -84 | -13 ± 18 | -78 ± 9 | LC | NT | EN |
| *Pericrocotus roseus* | 157,052 | -34 | -49 | 8 | -65 | -56 | -68 | -25 ± 29 | -63 ± 6 | LC | NT | EN |
| *Pericrocotus solaris* | 58,288 | -47 | -76 | -28 | -78 | -95 | -95 | -50 ± 24 | -89 ± 10 | LC | VU | CR |
| *Pericrocotus speciosus* | 131,280 | -32 | -70 | 8 | -46 | -54 | -27 | -31 ± 39 | -42 ± 14 | LC | VU | VU |
| *Pernis ptilorhynchus* | 270,374 | 4 | -26 | 7 | -46 | -49 | -89 | -5 ± 18 | -61 ± 24 | LC | NT | EN |
| *Phaenicophaeus tristis* | 345,054 | -14 | -64 | 16 | -57 | -50 | -66 | -21 ± 40 | -58 ± 8 | LC | NT | EN |
| *Phalacrocorax carbo* | 15,469 | -69 | -99 | -56 | -93 | -99 | -93 | -74 ± 22 | -95 ± 4 | VU | EN | CR |
| *Phalacrocorax fuscicollis* | 46,931 | 59 | 26 | 139 | -39 | -34 | 555 | 74 ± 58 | 161 ± 341 | LC | LC | LC |
| *Phalaropus lobatus* | 141,863 | 18 | 126 | 177 | -27 | 163 | 211 | 107 ± 81 | 115 ± 126 | LC | LC | LC |
| *Philomachus pugnax* | 121,471 | -10 | -10 | 40 | -82 | -73 | -98 | 7 ± 29 | -85 ± 13 | LC | LC | CR |
| *Phodilus badius* | 71,662 | -29 | -81 | -57 | -86 | -98 | -94 | -56 ± 26 | -93 ± 6 | LC | EN | CR |
| *Phoenicurus auroreus* | 31,682 | -48 | -37 | 136 | -66 | -92 | -88 | 17 ± 103 | -82 ± 14 | LC | LC | CR |
| *Phoenicurus frontalis* | 83,168 | -36 | -64 | -82 | -81 | -95 | -64 | -61 ± 23 | -80 ± 16 | LC | EN | EN |
| *Phylloscopus armandii* | 46,419 | -43 | 14 | 117 | -50 | -60 | -29 | 30 ± 81 | -46 ± 15 | LC | LC | VU |
| *Phylloscopus borealis* | 239,642 | 30 | 38 | 56 | 5 | 24 | 15 | 41 ± 13 | 15 ± 10 | LC | LC | LC |
| *Phylloscopus borealoides* | 151,997 | 42 | -33 | 22 | -19 | 127 | -23 | 10 ± 39 | 28 ± 86 | LC | LC | LC |
| *Phylloscopus cantator* | 75,412 | -45 | -91 | -25 | -92 | -94 | -96 | -54 ± 34 | -94 ± 2 | LC | EN | CR |
| *Phylloscopus claudiae* | 99,445 | -34 | -61 | 6 | -37 | -47 | 53 | -30 ± 34 | -10 ± 55 | LC | VU | NT |
| *Phylloscopus coronatus* | 207,678 | 50 | 44 | 136 | 36 | 156 | 116 | 76 ± 51 | 103 ± 61 | LC | LC | LC |
| *Phylloscopus examinandus* | 16,813 | 310 | 456 | 539 | 231 | 808 | 1747 | 435 ± 116 | 929 ± 765 | VU | LC | LC |
| *Phylloscopus fuscatus* | 312,147 | -6 | -7 | 13 | -43 | -69 | -45 | 0 ± 11 | -53 ± 15 | LC | LC | EN |
| *Phylloscopus humei* | 43,082 | -39 | -44 | 5 | -73 | -88 | -85 | -26 ± 27 | -82 ± 8 | LC | NT | CR |
| *Phylloscopus inornatus* | 352,621 | -4 | 0 | 25 | -15 | -35 | 18 | 7 ± 16 | -11 ± 27 | LC | LC | NT |
| *Phylloscopus intensior* | 46,506 | -25 | -66 | -8 | -58 | -70 | -23 | -33 ± 30 | -51 ± 24 | LC | VU | EN |

| | | | | | | | | | | | | |
|---|---|---|---|---|---|---|---|---|---|---|---|---|
| *Phylloscopus maculipennis* | 6,654 | -48 | -88 | 17 | -94 | -92 | -93 | -39 ± 53 | -93 ± 1 | VU | VU | CR |
| *Phylloscopus plumbeitarsus* | 230,428 | 3 | -50 | 21 | -29 | -33 | 43 | -9 ± 37 | -7 ± 43 | LC | NT | NT |
| *Phylloscopus proregulus* | 27,928 | -49 | -82 | -22 | -88 | -98 | -98 | -51 ± 30 | -94 ± 6 | NT | EN | CR |
| *Phylloscopus pulcher* | 6,179 | -73 | -86 | -24 | -86 | -100 | -95 | -61 ± 32 | -93 ± 7 | VU | EN | CR |
| *Phylloscopus reguloides* | 50,969 | -44 | -62 | 10 | -73 | -90 | -49 | -32 ± 37 | -71 ± 20 | LC | VU | EN |
| *Phylloscopus ricketti* | 117,897 | -18 | -51 | -12 | -47 | -72 | -44 | -27 ± 21 | -54 ± 16 | LC | NT | EN |
| *Phylloscopus schwarzi* | 221,290 | -3 | -60 | 6 | -39 | -67 | -13 | -19 ± 36 | -40 ± 27 | LC | NT | VU |
| *Phylloscopus subaffinis* | 3,291 | -81 | -87 | -46 | -89 | -96 | -98 | -71 ± 22 | -94 ± 5 | EN | EN | CR |
| *Phylloscopus tenellipes* | 204,603 | 6 | -49 | 19 | -9 | -27 | -78 | -8 ± 36 | -38 ± 36 | LC | NT | VU |
| *Phylloscopus trochiloides* | 66,603 | -17 | -1 | 26 | -32 | -53 | -72 | 3 ± 22 | -52 ± 20 | LC | LC | EN |
| *Phylloscopus yunnanensis* | 73,027 | -53 | -67 | -2 | -87 | -97 | -86 | -41 ± 34 | -90 ± 6 | LC | VU | CR |
| *Picumnus innominatus* | 56,273 | -46 | -65 | -37 | -83 | -95 | -90 | -50 ± 14 | -89 ± 6 | LC | VU | CR |
| *Picus canus* | 88,650 | -58 | -88 | -67 | -91 | -99 | -99 | -71 ± 16 | -96 ± 5 | LC | EN | CR |
| *Picus chlorolophus* | 95,031 | -32 | -69 | -21 | -61 | -81 | -87 | -41 ± 25 | -76 ± 13 | LC | VU | EN |
| *Picus erythropygius* | 103,664 | -17 | -31 | 37 | -46 | -74 | -22 | -4 ± 36 | -48 ± 26 | LC | NT | VU |
| *Picus flavinucha* | 110,383 | -48 | -65 | -42 | -89 | -97 | -97 | -52 ± 12 | -94 ± 5 | LC | EN | CR |
| *Picus miniaceus* | 39,554 | -29 | -38 | -40 | -61 | -83 | -87 | -36 ± 6 | -77 ± 14 | LC | VU | EN |
| *Picus puniceus* | 23,876 | -22 | -51 | -24 | -68 | -96 | -72 | -32 ± 16 | -79 ± 15 | NT | VU | EN |
| *Picus viridanus* | 42,792 | -72 | -97 | -75 | -91 | -100 | -97 | -81 ± 14 | -96 ± 4 | LC | CR | CR |
| *Picus vittatus* | 91,125 | -27 | -24 | 25 | -35 | -68 | -78 | -9 ± 29 | -60 ± 22 | LC | NT | EN |
| *Platalea minor* | 367,339 | 17 | 33 | 31 | 14 | 20 | 44 | 27 ± 8 | 26 ± 16 | LC | LC | LC |
| *Platylophus galericulatus* | 27,675 | -13 | -3 | -44 | -83 | -67 | -80 | -20 ± 21 | -77 ± 9 | NT | NT | EN |
| *Platysmurus leucopterus* | 24,077 | 24 | -71 | 5 | 37 | -92 | -92 | -14 ± 50 | -49 ± 74 | NT | NT | VU |
| *Plegadis falcinellus* | 60,352 | 178 | 353 | 362 | 227 | 433 | 293 | 297 ± 104 | 318 ± 105 | LC | LC | LC |
| *Pluvialis fulva* | 78,856 | 61 | 44 | 168 | -72 | 166 | 124 | 91 ± 67 | 73 ± 127 | LC | LC | LC |
| *Pluvialis squatarola* | 25,440 | 91 | 114 | 144 | -9 | 461 | -95 | 117 ± 26 | 119 ± 299 | NT | LC | LC |
| *Podiceps cristatus* | 41,507 | 78 | 17 | 102 | 71 | -18 | 89 | 65 ± 44 | 47 ± 58 | LC | LC | LC |
| *Polihierax insignis* | 102,683 | -13 | 10 | 88 | -36 | -61 | 51 | 28 ± 53 | -15 ± 59 | LC | LC | NT |
| *Polyplectron bicalcaratum* | 107,142 | -43 | -70 | -60 | -82 | -96 | -96 | -58 ± 14 | -91 ± 8 | LC | EN | CR |
| *Pomatorhinus erythrogenys* | 16,551 | -69 | -58 | -31 | -97 | -97 | -100 | -53 ± 20 | -98 ± 2 | VU | EN | CR |
| *Pomatorhinus ferruginosus* | 50,832 | -22 | -30 | 90 | -43 | -83 | 13 | 13 ± 67 | -38 ± 48 | LC | LC | VU |
| *Pomatorhinus hypoleucos* | 126,791 | -39 | -85 | -62 | -87 | -89 | -77 | -62 ± 23 | -85 ± 6 | LC | EN | CR |
| *Pomatorhinus ochraceiceps* | 39,778 | -51 | -93 | -88 | -90 | -98 | -99 | -77 ± 23 | -96 ± 5 | LC | EN | CR |

| | | | | | | | | | | | | |
|---|---|---|---|---|---|---|---|---|---|---|---|---|
| *Pomatorhinus schisticeps* | 91,616 | -48 | -73 | -42 | -76 | -97 | -97 | -54 ± 16 | -90 ± 12 | LC | EN | CR |
| *Porphyrio porphyrio* | 193,958 | 25 | 96 | 76 | 1 | 110 | 145 | 66 ± 36 | 85 ± 75 | LC | LC | LC |
| *Porzana bicolor* | 21,937 | -24 | -90 | -19 | -72 | -98 | -95 | -44 ± 40 | -88 ± 14 | NT | VU | CR |
| *Porzana cinerea* | 101,621 | 19 | 47 | 43 | -47 | -75 | -100 | 37 ± 15 | -74 ± 26 | LC | LC | EN |
| *Porzana fusca* | 100,511 | 7 | -7 | 99 | -71 | 83 | -80 | 33 ± 57 | -23 ± 92 | LC | LC | NT |
| *Porzana pusilla* | 32,661 | 29 | 155 | 123 | 36 | -91 | -86 | 102 ± 65 | -47 ± 72 | LC | LC | VU |
| *Prinia flaviventris* | 234,776 | 3 | -11 | 7 | -58 | 16 | 33 | 0 ± 9 | -3 ± 49 | LC | LC | NT |
| *Prinia hodgsonii* | 269,616 | -9 | -19 | 44 | -28 | -23 | 46 | 5 ± 34 | -2 ± 41 | LC | LC | NT |
| *Prinia inornata* | 235,629 | -1 | -8 | 10 | -54 | -66 | -27 | 0 ± 9 | -49 ± 20 | LC | LC | VU |
| *Prinia polychroa* | 134,792 | -11 | 4 | 1 | -49 | -81 | -56 | -2 ± 8 | -62 ± 17 | LC | NT | EN |
| *Prinia rufescens* | 263,126 | -30 | -59 | -26 | -64 | -68 | -72 | -38 ± 18 | -68 ± 4 | LC | VU | EN |
| *Prinia superciliaris* | 43,391 | -44 | -67 | -15 | -74 | -94 | -91 | -42 ± 26 | -86 ± 11 | LC | VU | CR |
| *Prionochilus maculatus* | 33,015 | -19 | -28 | -27 | -54 | -52 | -86 | -25 ± 5 | -64 ± 19 | LC | NT | EN |
| *Prionochilus percussus* | 38,362 | 45 | 10 | 0 | -34 | -18 | -63 | 19 ± 24 | -38 ± 23 | LC | LC | VU |
| *Prionochilus thoracicus* | 65,719 | 16 | 79 | 61 | 20 | 181 | -63 | 52 ± 32 | 46 ± 124 | LC | LC | LC |
| *Psittacula alexandri* | 115,784 | -34 | -39 | -29 | -74 | -93 | -96 | -34 ± 5 | -88 ± 12 | LC | VU | CR |
| *Psittacula eupatria* | 98,544 | 180 | 304 | 301 | 389 | 394 | 418 | 262 ± 71 | 400 ± 16 | LC | LC | LC |
| *Psittacula finschii* | 147,847 | -1 | -11 | 17 | -37 | -56 | -65 | 1 ± 14 | -53 ± 15 | LC | LC | EN |
| *Psittacula roseata* | 164,833 | 49 | 81 | 140 | 68 | 104 | 85 | 90 ± 46 | 86 ± 18 | LC | LC | LC |
| *Psittinus cyanurus* | 41,612 | -45 | -44 | -60 | -12 | -72 | -16 | -50 ± 9 | -33 ± 33 | LC | VU | VU |
| *Pterorhinus sannio* | 19,988 | -78 | -80 | -46 | -99 | -100 | -99 | -68 ± 19 | -99 ± 0 | VU | EN | CR |
| *Ptyonoprogne concolor* | 242,965 | -29 | -65 | -40 | -76 | -68 | -93 | -45 ± 18 | -79 ± 13 | LC | VU | EN |
| *Pycnonotus atriceps* | 261,260 | -3 | -32 | 33 | -37 | -37 | 3 | -1 ± 33 | -24 ± 23 | LC | NT | NT |
| *Pycnonotus aurigaster* | 283,749 | -9 | -24 | 20 | -32 | -43 | -7 | -4 ± 22 | -27 ± 19 | LC | NT | NT |
| *Pycnonotus blanfordi* | 351,630 | 8 | -1 | 25 | 20 | -21 | 28 | 11 ± 13 | 9 ± 26 | LC | LC | LC |
| *Pycnonotus brunneus* | 36,531 | 51 | 30 | 62 | 72 | 3 | 12 | 47 ± 16 | 29 ± 38 | LC | LC | LC |
| *Pycnonotus finlaysoni* | 275,422 | -11 | -54 | -14 | -46 | -48 | -20 | -26 ± 24 | -38 ± 16 | LC | NT | VU |
| *Pycnonotus flavescens* | 78,346 | 55 | -67 | 11 | -55 | -68 | -7 | 0 ± 62 | -43 ± 32 | LC | LC | VU |
| *Pycnonotus flaviventris* | 263,703 | -9 | -39 | -1 | -29 | -56 | -50 | -17 ± 20 | -45 ± 14 | LC | NT | VU |
| *Pycnonotus goiavier* | 228,878 | 23 | -32 | 33 | 13 | 33 | 63 | 8 ± 35 | 36 ± 25 | LC | LC | LC |
| *Pycnonotus jocosus* | 118,806 | -20 | -69 | -40 | -81 | -46 | -44 | -43 ± 25 | -57 ± 21 | LC | VU | EN |
| *Pycnonotus melanicterus* | 55,548 | 99 | 65 | -67 | 101 | 416 | 341 | 32 ± 87 | 286 ± 165 | LC | LC | LC |
| *Pycnonotus plumosus* | 28,617 | 75 | 22 | 175 | 70 | -6 | -3 | 91 ± 78 | 20 ± 43 | NT | LC | LC |

| | | | | | | | | | | | | |
|---|---|---|---|---|---|---|---|---|---|---|---|---|
| *Pycnonotus simplex* | 33,127 | 41 | 32 | 7 | 2 | 4 | -68 | 27 ± 18 | -20 ± 41 | LC | LC | NT |
| *Pycnonotus striatus* | 30,043 | -25 | -43 | 77 | -61 | -83 | -81 | 3 ± 64 | -75 ± 12 | LC | LC | EN |
| *Pycnonotus xanthorrhous* | 28,597 | -79 | -95 | -53 | -86 | -100 | -99 | -76 ± 21 | -95 ± 8 | NT | EN | CR |
| *Rallina eurizonoides* | 102,107 | 88 | -13 | 113 | 19 | 183 | 254 | 63 ± 67 | 152 ± 121 | LC | LC | LC |
| *Rallina fasciata* | 53,492 | 34 | 10 | 195 | -45 | -30 | -20 | 80 ± 100 | -31 ± 13 | LC | LC | VU |
| *Rallus indicus* | 130,762 | -67 | -92 | -78 | -96 | -99 | -97 | -79 ± 12 | -97 ± 1 | LC | EN | CR |
| *Recurvirostra avosetta* | 18,616 | 39 | 131 | 196 | -71 | -37 | -91 | 122 ± 79 | -66 ± 27 | VU | LC | EN |
| *Reinwardtipicus validus* | 19,126 | 12 | -11 | -36 | -62 | -99 | -84 | -12 ± 24 | -82 ± 18 | VU | NT | CR |
| *Rhaphidura leucopygialis* | 28,850 | -20 | -18 | 3 | -43 | -55 | -80 | -12 ± 13 | -59 ± 19 | NT | NT | EN |
| *Rhinomyias brunneatus* | 437,234 | 19 | 21 | 10 | 24 | 13 | 20 | 17 ± 6 | 19 ± 5 | LC | LC | LC |
| *Rhinomyias olivaceus* | 10,597 | 106 | 134 | 110 | 202 | 62 | 149 | 116 ± 15 | 137 ± 71 | VU | LC | LC |
| *Rhinortha chlorophaea* | 41,719 | -36 | -58 | -53 | -70 | -94 | -88 | -49 ± 11 | -84 ± 13 | LC | VU | CR |
| *Rhipidura albicollis* | 111,781 | -49 | -80 | -13 | -78 | -92 | -93 | -47 ± 34 | -88 ± 9 | LC | VU | CR |
| *Rhipidura aureola* | 369,200 | -3 | -3 | -10 | -19 | -49 | -10 | -5 ± 4 | -26 ± 20 | LC | NT | NT |
| *Rhipidura javanica* | 299,709 | 27 | -20 | 27 | 28 | 5 | 33 | 11 ± 27 | 22 ± 15 | LC | LC | LC |
| *Rhipidura perlata* | 25,456 | 105 | 130 | 212 | 10 | 260 | 340 | 149 ± 56 | 203 ± 172 | NT | LC | LC |
| *Rhopodytes diardi* | 48,225 | -46 | -70 | -68 | -67 | -95 | -89 | -61 ± 13 | -84 ± 15 | LC | EN | CR |
| *Rhopodytes sumatranus* | 22,119 | 21 | 22 | 30 | -7 | -64 | -79 | 24 ± 5 | -50 ± 38 | NT | LC | VU |
| *Rhopodytes tristis* | 364,441 | 0 | -15 | 15 | -28 | -50 | -62 | 0 ± 15 | -46 ± 17 | LC | LC | VU |
| *Rhyacornis fuliginosa* | 54,628 | -36 | -59 | 21 | -58 | -66 | -47 | -25 ± 41 | -57 ± 10 | LC | NT | EN |
| *Rhyticeros subruficollis* | 27,329 | -37 | -44 | -38 | -82 | -87 | -74 | -40 ± 3 | -81 ± 6 | NT | VU | CR |
| *Rhyticeros undulatus* | 108,434 | -28 | -61 | -52 | -62 | -68 | -84 | -47 ± 17 | -71 ± 11 | LC | VU | EN |
| *Riparia chinensis* | 137,088 | -41 | -53 | -53 | -70 | -79 | -87 | -49 ± 7 | -79 ± 8 | LC | VU | EN |
| *Riparia riparia* | 226,274 | 1 | 1 | 38 | -28 | 61 | 39 | 13 ± 21 | 24 ± 46 | LC | LC | LC |
| *Rostratula benghalensis* | 130,624 | -21 | -17 | -6 | -81 | -78 | -99 | -15 ± 8 | -86 ± 11 | LC | NT | CR |
| *Rubigula cyaniventris* | 23,348 | 12 | -3 | -26 | -31 | 18 | -62 | -6 ± 19 | -25 ± 40 | NT | NT | NT |
| *Rubigula erythropthalmos* | 42,529 | 14 | -19 | 0 | -37 | -57 | -44 | -2 ± 16 | -46 ± 10 | LC | NT | VU |
| *Rubigula squamatus* | 21,047 | 15 | 40 | 50 | 51 | 30 | 6 | 35 ± 18 | 29 ± 23 | NT | LC | LC |
| *Sarkidiornis melanotos* | 441,214 | -1 | 8 | 10 | 10 | -12 | -49 | 6 ± 6 | -17 ± 30 | LC | LC | NT |
| *Saroglossa spiloptera* | 60,443 | -63 | -52 | -6 | -66 | -84 | -96 | -40 ± 31 | -82 ± 15 | LC | VU | CR |
| *Sasia abnormis* | 25,481 | 37 | 40 | -9 | 87 | 38 | -32 | 23 ± 28 | 31 ± 60 | NT | LC | LC |
| *Sasia ochracea* | 124,850 | -31 | -7 | 21 | 26 | -67 | 9 | -5 ± 26 | -11 ± 49 | LC | NT | NT |
| *Saxicola caprata* | 219,753 | 44 | 47 | 67 | 90 | 98 | 100 | 53 ± 13 | 96 ± 6 | LC | LC | LC |

| | | | | | | | | | | | | |
|---|---|---|---|---|---|---|---|---|---|---|---|---|
| *Saxicola ferreus* | 47,985 | -33 | -57 | -21 | -69 | -84 | -89 | -37 ± 19 | -81 ± 11 | LC | VU | CR |
| *Saxicola jerdoni* | 13,848 | -84 | -50 | -11 | -93 | -99 | -99 | -48 ± 37 | -97 ± 4 | VU | VU | CR |
| *Saxicola maurus* | 284,067 | -8 | -45 | -38 | -45 | -61 | -20 | -30 ± 20 | -42 ± 21 | LC | VU | VU |
| *Saxicola stejnegeri* | 82,494 | 81 | 94 | 248 | 168 | 267 | 565 | 141 ± 93 | 333 ± 207 | LC | LC | LC |
| *Scolopax rusticola* | 25,649 | -32 | -81 | -43 | -69 | -99 | -98 | -52 ± 26 | -89 ± 17 | NT | EN | CR |
| *Seicercus castaniceps* | 45,012 | -34 | -52 | 57 | -47 | -86 | -78 | -10 ± 59 | -70 ± 21 | LC | NT | EN |
| *Seicercus omeiensis* | 62,798 | -52 | -83 | -49 | -85 | -95 | -91 | -62 ± 19 | -90 ± 5 | LC | EN | CR |
| *Seicercus soror* | 151,106 | 5 | -22 | -32 | -42 | -64 | -4 | -16 ± 19 | -37 ± 30 | LC | NT | VU |
| *Seicercus tephrocephalus* | 53,844 | -37 | -55 | -12 | -54 | -88 | -88 | -35 ± 22 | -76 ± 20 | LC | VU | EN |
| *Seicercus valentini* | 59,982 | -42 | -73 | 46 | -67 | -78 | -46 | -23 ± 62 | -64 ± 16 | LC | NT | EN |
| *Spilopelia chinensis* | 345,789 | 6 | 21 | 31 | 10 | -19 | 30 | 19 ± 13 | 7 ± 25 | LC | LC | LC |
| *Spilornis cheela* | 230,093 | -33 | -63 | -25 | -54 | -89 | -83 | -40 ± 20 | -75 ± 19 | LC | VU | EN |
| *Spizixos canifrons* | 7,886 | -91 | -83 | -59 | -95 | -99 | -98 | -77 ± 17 | -97 ± 2 | VU | EN | CR |
| *Spodiopsar sericeus* | 10,729 | 0 | 63 | 12 | -26 | -94 | -98 | 25 ± 34 | -73 ± 40 | VU | LC | EN |
| *Stachyridopsis chrysaea* | 65,745 | -48 | -74 | -41 | -77 | -86 | -85 | -55 ± 18 | -83 ± 5 | LC | EN | CR |
| *Stachyridopsis rufifrons* | 112,765 | -49 | -74 | -37 | -80 | -95 | -97 | -53 ± 19 | -91 ± 9 | LC | EN | CR |
| *Stachyris erythroptera* | 24,563 | 93 | 86 | 54 | 31 | 14 | -5 | 78 ± 21 | 13 ± 18 | NT | LC | LC |
| *Stachyris maculata* | 10,857 | 127 | 229 | 185 | 219 | 261 | 145 | 180 ± 51 | 208 ± 59 | VU | LC | LC |
| *Stachyris nigriceps* | 76,747 | -42 | -59 | -32 | -90 | -97 | -85 | -45 ± 13 | -91 ± 6 | LC | VU | CR |
| *Stachyris nigricollis* | 23,450 | 51 | 47 | 32 | 9 | 47 | 4 | 43 ± 10 | 20 ± 24 | NT | LC | LC |
| *Stachyris poliocephala* | 16,825 | 27 | 38 | 9 | -12 | 15 | 10 | 25 ± 14 | 4 ± 14 | VU | LC | LC |
| *Stachyris strialata* | 59,526 | -39 | -64 | -11 | -80 | -86 | -85 | -38 ± 27 | -84 ± 3 | LC | VU | CR |
| *Stercorarius pomarinus* | 12,188 | 635 | 76 | 706 | 661 | 174 | -85 | 472 ± 345 | 250 ± 379 | VU | LC | LC |
| *Sterna hirundo* | 57,692 | 155 | 80 | 217 | 15 | 426 | 380 | 151 ± 68 | 274 ± 225 | LC | LC | LC |
| *Sterna sumatrana* | 66,136 | 21 | 3 | -3 | 43 | 6 | -79 | 7 ± 12 | -10 ± 62 | LC | LC | NT |
| *Sternula albifrons* | 31,256 | 396 | 697 | 712 | 858 | 1236 | 1299 | 601 ± 178 | 1131 ± 239 | LC | LC | LC |
| *Streptopelia chinensis* | 340,372 | 3 | 15 | 35 | -15 | -43 | 4 | 18 ± 16 | -18 ± 24 | LC | LC | NT |
| *Streptopelia orientalis* | 148,684 | -34 | -40 | -38 | -50 | -77 | -79 | -38 ± 3 | -69 ± 16 | LC | VU | EN |
| *Streptopelia tranquebarica* | 290,808 | 16 | 34 | 38 | -15 | -11 | 17 | 29 ± 12 | -3 ± 18 | LC | LC | NT |
| *Strix leptogrammica* | 154,159 | -44 | -78 | -49 | -83 | -93 | -93 | -57 ± 18 | -90 ± 6 | LC | EN | CR |
| *Strix seloputo* | 57,197 | 10 | 9 | -1 | -63 | -69 | -48 | 6 ± 6 | -60 ± 11 | LC | LC | EN |
| *Sturnia malabarica* | 158,837 | -1 | -20 | -4 | -56 | -65 | -20 | -8 ± 11 | -47 ± 24 | LC | NT | VU |
| *Sturnia pagodarum* | 354,331 | 3 | 27 | 46 | 37 | 7 | -13 | 25 ± 22 | 10 ± 25 | LC | LC | LC |

| | | | | | | | | | | | | |
|---|---|---|---|---|---|---|---|---|---|---|---|---|
| *Sturnia sinensis* | 100,357 | -54 | -5 | 65 | -73 | -4 | -50 | 2 ± 60 | -42 ± 35 | LC | LC | VU |
| *Sturnus vulgaris* | 116,397 | -20 | -12 | -83 | -45 | -82 | -28 | -39 ± 39 | -52 ± 27 | LC | VU | EN |
| *Surniculus lugubris* | 223,323 | -15 | -31 | -20 | -65 | -51 | -70 | -22 ± 9 | -62 ± 10 | LC | NT | EN |
| *Syrmaticus humiae* | 20,201 | -82 | -95 | -61 | -88 | -100 | -98 | -79 ± 17 | -95 ± 6 | NT | EN | CR |
| *Tachybaptus ruficollis* | 241,114 | 5 | 49 | 19 | -14 | 74 | 105 | 24 ± 22 | 55 ± 62 | LC | LC | LC |
| *Tadorna ferruginea* | 10,111 | -27 | 71 | 60 | -1 | 814 | -100 | 35 ± 54 | 238 ± 502 | VU | LC | LC |
| *Tadorna tadorna* | 286,934 | 28 | 27 | 20 | -53 | -65 | 6 | 25 ± 5 | -37 ± 38 | LC | LC | VU |
| *Tarsiger chrysaeus* | 15,289 | -79 | -70 | 57 | -28 | -93 | -93 | -30 ± 76 | -71 ± 38 | VU | VU | EN |
| *Tarsiger cyanurus* | 17,621 | -70 | -88 | -19 | -89 | -99 | -95 | -59 ± 36 | -95 ± 5 | VU | EN | CR |
| *Tarsiger rufilatus* | 10,776 | -70 | -85 | -71 | -93 | -99 | -99 | -75 ± 8 | -97 ± 4 | VU | EN | CR |
| *Temnurus temnurus* | 1,024 | -99 | -100 | -100 | -100 | -100 | -100 | -100 ± 0 | -100 ± 0 | EN | EX | EX |
| *Thalasseus bengalensis* | 32,944 | 12 | -99 | -99 | -100 | -100 | -100 | -62 ± 64 | -100 ± 0 | LC | EN | EX |
| *Thalasseus bergii* | 73,153 | 43 | 106 | 44 | 60 | 76 | 0 | 64 ± 36 | 45 ± 40 | LC | LC | LC |
| *Threskiornis melanocephalus* | 23,731 | 15 | 192 | 270 | -28 | 289 | 615 | 159 ± 131 | 292 ± 321 | NT | LC | LC |
| *Timalia pileata* | 175,443 | -26 | -8 | 43 | -45 | -36 | -44 | 3 ± 36 | -41 ± 5 | LC | LC | VU |
| *Todiramphus chloris* | 36,387 | 116 | 212 | 285 | 97 | 417 | 529 | 204 ± 85 | 348 ± 224 | LC | LC | LC |
| *Treron apicauda* | 29,995 | -47 | -68 | 36 | -86 | -95 | -94 | -26 ± 55 | -92 ± 5 | NT | NT | CR |
| *Treron bicinctus* | 76,031 | -6 | -35 | -52 | -15 | -68 | -70 | -31 ± 24 | -51 ± 31 | LC | VU | EN |
| *Treron curvirostra* | 183,395 | -2 | -41 | 7 | -34 | -55 | -38 | -12 ± 25 | -42 ± 11 | LC | NT | VU |
| *Treron phayrei* | 114,920 | 68 | 172 | 220 | 196 | 223 | 220 | 153 ± 78 | 213 ± 15 | LC | LC | LC |
| *Treron phoenicopterus* | 36,903 | -3 | 8 | 113 | -15 | -19 | 0 | 39 ± 64 | -11 ± 10 | LC | LC | NT |
| *Treron seimundi* | 11,939 | -50 | -64 | -62 | -85 | -82 | -100 | -59 ± 8 | -89 ± 10 | VU | EN | CR |
| *Treron sieboldii* | 116,251 | -43 | -86 | -24 | -74 | -99 | -86 | -51 ± 32 | -86 ± 13 | LC | EN | CR |
| *Treron sphenurus* | 51,394 | -57 | -82 | -31 | -85 | -98 | -94 | -57 ± 25 | -92 ± 6 | LC | EN | CR |
| *Treron vernans* | 41,969 | 137 | 312 | 275 | -9 | 565 | 730 | 242 ± 92 | 428 ± 388 | LC | LC | LC |
| *Trichastoma bicolor* | 11,153 | 18 | 200 | 164 | 139 | 192 | 103 | 127 ± 96 | 145 ± 45 | VU | LC | LC |
| *Trichastoma malaccense* | 14,118 | 64 | 100 | 139 | 106 | 122 | 101 | 101 ± 38 | 110 ± 11 | VU | LC | LC |
| *Trichastoma rostratum* | 37,880 | 40 | 77 | 3 | 34 | -39 | -15 | 40 ± 37 | -6 ± 37 | LC | LC | NT |
| *Tricholestes criniger* | 21,642 | 15 | 18 | -6 | 4 | -8 | -61 | 9 ± 13 | -22 ± 34 | NT | LC | NT |
| *Tringa brevipes* | 78,915 | 129 | 222 | 169 | 346 | 362 | 66 | 173 ± 47 | 258 ± 166 | LC | LC | LC |
| *Tringa erythropus* | 92,079 | 25 | 44 | 26 | -51 | -67 | -100 | 32 ± 11 | -73 ± 25 | LC | LC | EN |
| *Tringa glareola* | 143,768 | -8 | 31 | 52 | -54 | -27 | -83 | 25 ± 30 | -55 ± 28 | LC | LC | EN |
| *Tringa guttifer* | 8,609 | 158 | 224 | 82 | 104 | -16 | -98 | 155 ± 71 | -3 ± 102 | VU | LC | NT |

| | | | | | | | | | | | | |
|---|---|---|---|---|---|---|---|---|---|---|---|---|
| *Tringa nebularia* | 125,931 | 47 | 117 | 129 | 42 | -9 | -29 | 98 ± 45 | 1 ± 37 | LC | LC | LC |
| *Tringa ochropus* | 140,135 | 38 | 99 | 125 | 59 | 102 | -65 | 88 ± 45 | 32 ± 87 | LC | LC | LC |
| *Tringa stagnatilis* | 83,789 | 54 | 11 | 136 | -57 | -38 | -55 | 67 ± 63 | -50 ± 10 | LC | LC | VU |
| *Tringa totanus* | 39,795 | 179 | 355 | 499 | 122 | 757 | 785 | 344 ± 160 | 555 ± 375 | LC | LC | LC |
| *Trochalopteron melanostigma* | 27,563 | -44 | -60 | 77 | -84 | -99 | -85 | -9 ± 75 | -90 ± 9 | NT | NT | CR |
| *Turdinus abbotti* | 102,597 | -49 | -69 | -54 | -64 | -48 | -90 | -58 ± 11 | -67 ± 21 | LC | EN | EN |
| *Turnix suscitator* | 210,572 | 1 | 34 | 37 | -51 | 17 | 64 | 24 ± 19 | 10 ± 58 | LC | LC | LC |
| *Turnix sylvaticus* | 76,835 | 64 | 181 | 174 | 99 | 248 | 523 | 140 ± 65 | 290 ± 215 | LC | LC | LC |
| *Turnix tanki* | 354,448 | 10 | 27 | 21 | 1 | 39 | 64 | 19 ± 9 | 35 ± 31 | LC | LC | LC |
| *Tyto alba* | 254,550 | -10 | -15 | 23 | -5 | 38 | 48 | 0 ± 20 | 27 ± 28 | LC | LC | LC |
| *Upupa epops* | 297,146 | -17 | -22 | 21 | -64 | -20 | 20 | -6 ± 24 | -21 ± 42 | LC | NT | NT |
| *Urocissa erythroryncha* | 140,498 | -8 | 26 | 32 | -2 | -49 | -5 | 17 ± 21 | -19 ± 26 | LC | LC | NT |
| *Vanellus cinereus* | 157,118 | 9 | 39 | 49 | -23 | -18 | -95 | 33 ± 21 | -45 ± 43 | LC | LC | VU |
| *Vanellus duvaucelii* | 244,290 | -5 | 19 | 22 | -3 | -40 | -80 | 12 ± 15 | -41 ± 38 | LC | LC | VU |
| *Vanellus indicus* | 250,534 | 6 | 13 | 42 | -32 | -6 | 48 | 20 ± 19 | 3 ± 41 | LC | LC | LC |
| *Xenus cinereus* | 23,110 | -5 | 11 | 29 | -12 | 73 | -70 | 12 ± 17 | -3 ± 72 | NT | LC | NT |
| *Zanclostomus curvirostris* | 35,428 | -21 | -42 | -38 | -55 | -87 | -81 | -34 ± 11 | -74 ± 17 | LC | VU | EN |
| *Zanclostomus javanicus* | 24,824 | -9 | -30 | 21 | -54 | -76 | -37 | -6 ± 26 | -55 ± 19 | NT | NT | EN |

**Table S7** Predicted current suitable habitat for each plant species, projected change by 2070, and estimated conservation status using three earth system models and two RCPs.

| species | suitable habitat at present ($km^2$) | % change in suitable habitat under RCP2.6 | | | % change in suitable habitat under RCP8.5 | | | mean of suitable habitat change | | Conservation Status | | |
|---|---|---|---|---|---|---|---|---|---|---|---|---|
| | | CNRM-CM5 | GFDL-CM3 | HadGEM2-ES | CNRM-CM5 | GFDL-CM3 | HadGEM2-ES | RCP 2.6 | RCP 8.5 | Current | RCP 2.6 | RCP 8.5 |
| *Abrus fruticulosus* | 317,309 | -5 | -5 | 5 | -3 | -6 | 17 | -2 ± 6 | 3 ± 12 | LC | NT | LC |
| *Abrus precatorius* | 330,985 | 30 | 14 | 7 | 38 | -23 | 49 | 17 ± 12 | 21 ± 39 | LC | LC | LC |
| *Acacia caesia* | 88,087 | -69 | -91 | -69 | -95 | -97 | -98 | -76 ± 13 | -97 ± 2 | LC | EN | CR |
| *Acacia comosa* | 17,161 | 20 | 24 | 13 | 79 | 29 | 594 | 19 ± 6 | 234 ± 313 | VU | LC | LC |
| *Acacia concinna* | 115,001 | -50 | -86 | -70 | -90 | -99 | -92 | -69 ± 18 | -94 ± 5 | LC | EN | CR |
| *Acacia farnesiana* | 83,551 | -50 | -82 | -19 | -92 | -96 | -96 | -51 ± 32 | -95 ± 2 | LC | EN | CR |
| *Acacia harmandiana* | 76,555 | -61 | -85 | -34 | -71 | -99 | -89 | -60 ± 25 | -86 ± 14 | LC | EN | CR |
| *Acacia leucophloea* | 27,378 | -91 | -100 | -100 | -100 | -100 | -100 | -97 ± 5 | -100 ± 0 | NT | CR | EX |
| *Acacia megaladena* | 196,366 | -7 | -26 | 36 | -11 | -32 | 47 | 1 ± 32 | 2 ± 41 | LC | LC | LC |
| *Acacia pennata* | 82,200 | -70 | -89 | -64 | -91 | -98 | -100 | -74 ± 13 | -96 ± 5 | LC | EN | CR |
| *Acer calcaratum* | 77,330 | -37 | -99 | -65 | -57 | -80 | -90 | -67 ± 31 | -76 ± 17 | LC | EN | EN |
| *Acmella paniculata* | 44,901 | -71 | -70 | 15 | -90 | -99 | -84 | -42 ± 49 | -91 ± 8 | LC | VU | CR |
| *Acrocarpus fraxinifolius* | 117,355 | 12 | -23 | 10 | -8 | 7 | -21 | 0 ± 19 | -8 ± 14 | LC | LC | NT |
| *Acronychia pedunculata* | 89,709 | -21 | -83 | -41 | -57 | -82 | -68 | -48 ± 32 | -69 ± 13 | LC | VU | EN |
| *Actinodaphne henryi* | 99,817 | -27 | -47 | -25 | -72 | -26 | -86 | -33 ± 12 | -61 ± 32 | LC | VU | EN |
| *Adenanthera microsperma* | 117,893 | 0 | -14 | 122 | -47 | -58 | 146 | 36 ± 75 | 14 ± 114 | LC | LC | LC |
| *Adenanthera pavonina* | 127,761 | -12 | -17 | 8 | -39 | -36 | -49 | -7 ± 13 | -41 ± 7 | LC | NT | VU |
| *Adenostemma lavenia* | 35,220 | -51 | -92 | -44 | -73 | -99 | -63 | -62 ± 26 | -79 ± 19 | LC | EN | EN |
| *Adenostemma viscosum* | 83,643 | -47 | -88 | -29 | -71 | -95 | -72 | -54 ± 30 | -79 ± 14 | LC | EN | EN |
| *Adinandra integerrima* | 66,382 | -25 | -81 | -44 | -77 | -93 | -82 | -50 ± 29 | -84 ± 9 | LC | VU | CR |
| *Aegle marmelos* | 392,597 | -4 | 26 | 10 | 0 | -36 | 45 | 10 ± 15 | 3 ± 40 | LC | LC | LC |
| *Aeschynomene indica* | 353,111 | -9 | 23 | 47 | 12 | -17 | 45 | 20 ± 28 | 13 ± 31 | LC | LC | LC |
| *Aesculus assamica* | 103,231 | -7 | -10 | 77 | 7 | 12 | 72 | 20 ± 50 | 30 ± 36 | LC | LC | LC |
| *Afgekia filipes* | 51,238 | -51 | -47 | -21 | -51 | -92 | -93 | -40 ± 16 | -79 ± 24 | LC | VU | EN |
| *Afgekia mahidolae* | 35,080 | -83 | -69 | -47 | -92 | -100 | -83 | -67 ± 18 | -92 ± 8 | LC | EN | CR |
| *Afgekia sericea* | 224,382 | 4 | -37 | 19 | -9 | -27 | 44 | -4 ± 29 | 3 ± 37 | LC | NT | LC |
| *Afzelia xylocarpa* | 100,821 | 22 | 8 | 7 | 27 | -58 | 15 | 12 ± 8 | -5 ± 46 | LC | LC | NT |
| *Aganope heptaphylla* | 5,910 | 54 | -75 | -68 | -76 | -97 | -99 | -30 ± 72 | -91 ± 13 | VU | VU | CR |

| | | | | | | | | | | | | |
|---|---|---|---|---|---|---|---|---|---|---|---|---|
| *Aganope thyrsiflora* | 233,442 | 18 | -27 | 1 | -19 | 55 | 70 | -2 ± 23 | 35 ± 48 | LC | NT | LC |
| *Agapetes bracteata* | 98,296 | -86 | -100 | -87 | -96 | -100 | -99 | -91 ± 8 | -98 ± 2 | LC | CR | CR |
| *Agapetes hosseana* | 149,469 | -35 | -80 | 31 | -59 | -65 | -69 | -28 ± 56 | -64 ± 5 | LC | NT | EN |
| *Agapetes lobbii* | 44,432 | -42 | -100 | -77 | -73 | -99 | -97 | -73 ± 29 | -90 ± 15 | LC | EN | CR |
| *Agapetes loranthiflora* | 5,076 | -85 | -100 | -95 | -99 | -100 | -100 | -93 ± 8 | -100 ± 0 | VU | CR | EX |
| *Agapetes parishii* | 135,191 | -11 | 24 | 101 | 142 | 127 | 206 | 38 ± 57 | 158 ± 42 | LC | LC | LC |
| *Agapetes saxicola* | 29,995 | -63 | -100 | -100 | -93 | -95 | -97 | -88 ± 21 | -95 ± 2 | NT | CR | CR |
| *Aglaia argentea* | 3,522 | -29 | 23 | -98 | 22 | 65 | -97 | -34 ± 61 | -3 ± 84 | EN | VU | NT |
| *Aglaia chittagonga* | 136,728 | -42 | -61 | 21 | -70 | -78 | -36 | -28 ± 43 | -61 ± 22 | LC | NT | EN |
| *Aglaia crassinervia* | 17,849 | -30 | -81 | -48 | -89 | -100 | -67 | -53 ± 26 | -85 ± 16 | VU | EN | CR |
| *Aglaia edulis* | 3,280 | -50 | -63 | -93 | -62 | 26 | -100 | -69 ± 22 | -45 ± 65 | EN | EN | VU |
| *Aglaia elaeagnoidea* | 99,373 | -34 | -70 | -70 | -80 | -93 | -79 | -58 ± 21 | -84 ± 8 | LC | EN | CR |
| *Aglaia elliptica* | 245,389 | -14 | -33 | 18 | -2 | 105 | 96 | -9 ± 26 | 66 ± 59 | LC | NT | LC |
| *Aglaia forbesii* | 37,113 | -16 | -17 | 30 | -41 | -89 | 2 | -1 ± 27 | -43 ± 46 | LC | NT | VU |
| *Aglaia lawii* | 288,952 | -28 | -41 | -32 | -41 | -75 | -38 | -34 ± 7 | -51 ± 21 | LC | VU | EN |
| *Aglaia pachyphylla* | 9,082 | -7 | -14 | 411 | 167 | 118 | 364 | 130 ± 244 | 216 ± 130 | VU | LC | LC |
| *Aglaia perviridis* | 48,315 | -55 | -75 | -41 | -79 | -96 | -71 | -57 ± 17 | -82 ± 13 | LC | EN | CR |
| *Aglaia tenuicaulis* | 1,476 | 714 | 2427 | 1023 | 3106 | 3390 | 3394 | 1388 ± 913 | 3297 ± 165 | EN | LC | LC |
| *Ailanthus triphysa* | 102,538 | -42 | -22 | -3 | -55 | -36 | -60 | -22 ± 19 | -50 ± 13 | LC | NT | VU |
| *Akschindlium godefroyanum* | 258,471 | 12 | 24 | 24 | 34 | -28 | 48 | 20 ± 7 | 18 ± 41 | LC | LC | LC |
| *Alangium chinense* | 114,860 | -37 | -62 | -12 | -71 | -87 | -33 | -37 ± 25 | -63 ± 27 | LC | VU | EN |
| *Alangium salviifolium* | 106,932 | 2 | 28 | 38 | 21 | 4 | -64 | 23 ± 19 | -13 ± 45 | LC | LC | NT |
| *Albizia chinensis* | 125,957 | -22 | -53 | 43 | -40 | -86 | -22 | -11 ± 49 | -49 ± 33 | LC | NT | VU |
| *Albizia lebbeck* | 187,200 | 10 | -19 | -14 | -5 | -50 | -10 | -8 ± 15 | -22 ± 25 | LC | NT | NT |
| *Albizia odoratissima* | 235,305 | -33 | -50 | 12 | -55 | -76 | -82 | -24 ± 32 | -71 ± 14 | LC | NT | EN |
| *Allophylus cobbe* | 313,472 | -2 | 26 | 23 | -25 | 20 | -3 | 16 ± 15 | -3 ± 22 | LC | LC | NT |
| *Alphonsea glabrifolia* | 50,869 | -49 | -36 | 78 | -79 | -97 | -70 | -2 ± 70 | -82 ± 14 | LC | NT | CR |
| *Alpinia blepharocalyx* | 73,492 | -13 | -66 | -14 | -28 | -66 | 19 | -31 ± 30 | -25 ± 43 | LC | VU | NT |
| *Alpinia galanga* | 415,447 | -5 | 0 | -27 | -15 | -71 | -25 | -11 ± 15 | -37 ± 30 | LC | NT | VU |
| *Alpinia javanica* | 26,168 | 52 | 51 | 78 | 41 | 19 | 30 | 60 ± 16 | 30 ± 11 | NT | LC | LC |
| *Alpinia malaccensis* | 226,440 | -38 | -38 | -21 | -63 | -88 | -62 | -32 ± 10 | -71 ± 15 | LC | VU | EN |
| *Alpinia oxymitra* | 541,103 | -4 | -55 | -4 | -8 | -31 | -10 | -21 ± 29 | -16 ± 13 | LC | NT | NT |
| *Alstonia rostrata* | 39,897 | -43 | -93 | -81 | -73 | -98 | -71 | -72 ± 26 | -81 ± 15 | LC | EN | CR |
| *Alstonia scholaris* | 99,470 | 2 | -5 | -4 | 35 | -33 | 15 | -2 ± 4 | 6 ± 35 | LC | NT | LC |
| *Alysicarpus vaginalis* | 322,375 | 24 | -35 | 17 | -25 | -28 | 45 | 2 ± 32 | -2 ± 41 | LC | LC | NT |
| *Amomum siamense* | 132,633 | -45 | -75 | -21 | -78 | -93 | -35 | -47 ± 27 | -68 ± 30 | LC | VU | EN |
| *Amomum uliginosum* | 258,838 | -36 | -46 | -6 | -34 | -74 | -65 | -29 ± 21 | -57 ± 21 | LC | NT | EN |
| *Amphicarpaea involucrata* | 113,863 | -34 | -82 | -14 | -70 | -96 | -75 | -43 ± 35 | -80 ± 14 | LC | VU | EN |
| *Anisoptera costata* | 229,759 | -21 | -66 | -69 | -71 | -83 | -92 | -52 ± 27 | -82 ± 11 | LC | EN | CR |

| | | | | | | | | | | | | |
|---|---|---|---|---|---|---|---|---|---|---|---|---|
| *Anisoptera scaphula* | 145,693 | -53 | -74 | -36 | -70 | -93 | -84 | -54 ± 19 | -82 ± 12 | LC | EN | CR |
| *Anneslea fragrans* | 127,310 | 30 | 28 | 67 | 83 | 74 | 131 | 41 ± 22 | 96 ± 31 | LC | LC | LC |
| *Anogeissus acuminata* | 100,829 | 19 | 46 | 16 | 27 | 17 | -15 | 27 ± 16 | 10 ± 22 | LC | LC | LC |
| *Antheroporum glaucum* | 27,683 | -15 | -63 | 29 | -73 | -77 | -85 | -16 ± 46 | -78 ± 6 | NT | NT | EN |
| *Antidesma ghaesembilla* | 199,457 | 13 | -5 | -6 | -15 | -40 | -50 | 1 ± 10 | -35 ± 18 | LC | LC | VU |
| *Antidesma sootepense* | 99,401 | 9 | 72 | 39 | 61 | 51 | 15 | 40 ± 32 | 42 ± 24 | LC | LC | LC |
| *Aphanamixis polystachya* | 95,601 | -1 | -33 | 3 | -39 | -49 | -63 | -10 ± 20 | -50 ± 12 | LC | NT | VU |
| *Aporosa octandra* | 72,668 | 19 | 7 | 54 | 39 | 113 | 49 | 27 ± 24 | 67 ± 40 | LC | LC | LC |
| *Aporosa villosa* | 106,461 | -6 | -6 | -19 | -13 | -44 | -27 | -10 ± 7 | -28 ± 16 | LC | NT | NT |
| *Aquilaria crassna* | 44,488 | -48 | -80 | -1 | -57 | -70 | -96 | -43 ± 40 | -74 ± 20 | LC | VU | EN |
| *Aquilaria malaccensis* | 173,730 | -7 | -4 | 0 | 1 | -10 | 6 | -4 ± 4 | -1 ± 8 | LC | NT | NT |
| *Archidendron clypearia* | 344,962 | -5 | -34 | -17 | -11 | -65 | -52 | -18 ± 15 | -42 ± 28 | LC | NT | VU |
| *Archidendron jiringa* | 119,599 | -24 | -45 | 0 | -19 | -66 | -81 | -23 ± 22 | -55 ± 32 | LC | NT | EN |
| *Ardisia sanguinolenta* | 8,344 | -8 | 19 | 133 | 128 | 346 | 529 | 48 ± 75 | 334 ± 201 | VU | LC | LC |
| *Artocarpus lacucha* | 129,655 | -4 | 36 | 17 | 22 | -5 | 23 | 16 ± 20 | 13 ± 16 | LC | LC | LC |
| *Artocarpus nitidus* | 157,585 | -37 | -72 | -43 | -63 | -96 | -75 | -51 ± 18 | -78 ± 16 | LC | EN | EN |
| *Artocarpus thailandica* | 99,558 | -26 | -71 | -5 | -58 | -88 | 38 | -34 ± 33 | -36 ± 66 | LC | VU | VU |
| *Arytera littoralis* | 147,986 | -20 | 47 | 25 | -19 | -59 | 13 | 18 ± 34 | -22 ± 36 | LC | LC | NT |
| *Baccaurea ramiflora* | 88,226 | 9 | -7 | 6 | 59 | 35 | 12 | 3 ± 8 | 35 ± 24 | LC | LC | LC |
| *Balakata baccata* | 43,102 | -7 | -18 | 2 | -8 | 53 | -35 | -7 ± 10 | 3 ± 45 | LC | NT | LC |
| *Barringtonia macrostachya* | 14,998 | 86 | 123 | 127 | 105 | 71 | 83 | 112 ± 23 | 86 ± 17 | VU | LC | LC |
| *Bauhinia bracteata* | 150,209 | 25 | -82 | 74 | -6 | 43 | 146 | 6 ± 80 | 61 ± 78 | LC | LC | LC |
| *Bauhinia glauca* | 399,015 | -13 | -33 | -10 | -27 | -34 | 12 | -19 ± 13 | -16 ± 24 | LC | NT | NT |
| *Bauhinia malabarica* | 105,335 | 63 | 103 | 35 | 91 | 118 | -42 | 67 ± 34 | 56 ± 86 | LC | LC | LC |
| *Bauhinia ornata* | 165,441 | -30 | -72 | 9 | -27 | -43 | -72 | -31 ± 41 | -47 ± 23 | LC | VU | VU |
| *Bauhinia pulla* | 225,870 | -4 | -15 | -23 | -12 | -72 | 84 | -14 ± 10 | 0 ± 78 | LC | NT | LC |
| *Bauhinia saccocalyx* | 482,701 | 2 | -9 | 0 | -5 | -2 | 7 | -2 ± 6 | 0 ± 6 | LC | NT | LC |
| *Bauhinia scandens* | 499,032 | -1 | -66 | -23 | -8 | -58 | 0 | -30 ± 33 | -22 ± 32 | LC | VU | NT |
| *Bauhinia variegata* | 67,142 | -44 | -28 | -16 | -54 | -47 | -68 | -29 ± 14 | -56 ± 11 | LC | NT | EN |
| *Bauhinia viridescens* | 190,972 | -35 | -38 | -29 | -64 | -82 | -83 | -34 ± 5 | -76 ± 11 | LC | VU | EN |
| *Beilschmiedia gammieana* | 36,395 | -39 | -59 | -42 | -58 | -52 | -77 | -47 ± 11 | -62 ± 14 | LC | VU | EN |
| *Bhesa robusta* | 125,382 | 7 | -75 | -39 | -36 | -74 | -43 | -36 ± 41 | -51 ± 20 | LC | VU | EN |
| *Blumea balsamifera* | 76,887 | -30 | -44 | 15 | -53 | -85 | -43 | -20 ± 30 | -60 ± 21 | LC | NT | EN |
| *Blumea fistulosa* | 205,106 | -49 | -69 | -36 | -87 | -96 | -43 | -51 ± 17 | -75 ± 28 | LC | EN | EN |
| *Blumea lacera* | 197,009 | -18 | 17 | 61 | -6 | -4 | 103 | 20 ± 40 | 31 ± 62 | LC | LC | LC |
| *Blumea mollis* | 292,084 | -24 | -21 | 19 | -35 | -19 | 46 | -9 ± 24 | -3 ± 43 | LC | NT | NT |
| *Blumeopsis flava* | 312,045 | 0 | -13 | 7 | -12 | -44 | 27 | -2 ± 10 | -10 ± 36 | LC | NT | NT |
| *Boesenbergia basispicata* | 147,393 | 1 | 18 | -23 | 68 | -51 | 84 | -1 ± 21 | 34 ± 74 | LC | NT | LC |
| *Boesenbergia longipes* | 38,887 | -53 | -21 | -13 | -62 | -82 | -72 | -29 ± 21 | -72 ± 10 | LC | NT | EN |

| | | | | | | | | | | | | |
|---|---|---|---|---|---|---|---|---|---|---|---|---|
| *Boesenbergia rotunda* | 278,286 | -28 | -16 | -20 | -12 | -70 | -16 | -21 ± 6 | -33 ± 33 | LC | NT | VU |
| *Bombax ceiba* | 142,779 | 24 | 31 | 74 | 45 | 10 | 59 | 43 ± 27 | 38 ± 26 | LC | LC | LC |
| *Bombax insigne* | 62,443 | 13 | -53 | -60 | 28 | -35 | -26 | -34 ± 40 | -11 ± 34 | LC | VU | NT |
| *Bouea oppositifolia* | 19,639 | -25 | -48 | -39 | -61 | -77 | -76 | -37 ± 12 | -72 ± 9 | VU | VU | EN |
| *Broussonetia kurzii* | 212,468 | -26 | -37 | 1 | -39 | -57 | -21 | -20 ± 20 | -39 ± 18 | LC | NT | VU |
| *Broussonetia papyrifera* | 200,099 | -27 | -15 | -26 | -62 | -68 | -91 | -23 ± 7 | -74 ± 15 | LC | NT | EN |
| *Butea monosperma* | 349,777 | 10 | -9 | 2 | -3 | -7 | 30 | 1 ± 9 | 7 ± 20 | LC | LC | LC |
| *Butea superba* | 169,344 | -3 | 71 | 84 | -8 | 34 | 103 | 51 ± 47 | 43 ± 56 | LC | LC | LC |
| *Caesalpinia digyna* | 353,332 | -5 | 29 | 37 | -63 | -17 | 48 | 20 ± 22 | -11 ± 56 | LC | LC | NT |
| *Caesalpinia hymenocarpa* | 379,106 | -7 | -19 | 10 | -25 | -38 | 6 | -5 ± 15 | -19 ± 23 | LC | NT | NT |
| *Caesalpinia sappan* | 315,775 | -13 | -3 | 35 | 11 | -25 | 47 | 6 ± 25 | 11 ± 36 | LC | LC | LC |
| *Cajanus crassus* | 292,996 | -41 | -49 | -63 | -81 | -86 | -54 | -51 ± 11 | -74 ± 17 | LC | EN | EN |
| *Cajanus goensis* | 141,436 | -45 | -86 | -70 | -90 | -95 | -97 | -67 ± 20 | -94 ± 3 | LC | EN | CR |
| *Cajanus scarabaeoides* | 337,789 | -7 | -43 | -3 | -20 | -21 | 16 | -18 ± 22 | -8 ± 21 | LC | NT | NT |
| *Callerya atropurpurea* | 50,544 | -54 | -74 | -66 | -75 | -87 | -88 | -64 ± 10 | -83 ± 7 | LC | EN | CR |
| *Callicarpa arborea* | 120,669 | 3 | 11 | 36 | -11 | -39 | 25 | 17 ± 17 | -8 ± 32 | LC | LC | NT |
| *Calophyllum calaba* | 69,363 | 83 | -49 | 52 | -56 | -4 | -35 | 28 ± 69 | -31 ± 26 | LC | LC | VU |
| *Calophyllum polyanthum* | 301,193 | 7 | 3 | -5 | 7 | -16 | 4 | 2 ± 6 | -2 ± 12 | LC | LC | NT |
| *Calycopteris floribunda* | 302,490 | -7 | 3 | 12 | -6 | -7 | 38 | 3 ± 10 | 8 ± 26 | LC | LC | LC |
| *Canarium denticulatum* | 65,620 | -46 | -75 | -62 | -63 | -94 | -80 | -61 ± 15 | -79 ± 15 | LC | EN | EN |
| *Canarium euphyllum* | 148,882 | -1 | -39 | 54 | -25 | -8 | 79 | 4 ± 47 | 15 ± 56 | LC | LC | LC |
| *Canarium littorale* | 1,322 | -97 | 210 | 1172 | 202 | 66 | 803 | 428 ± 662 | 357 ± 392 | EN | LC | LC |
| *Canarium strictum* | 46,285 | -8 | -47 | 21 | -26 | -40 | 182 | -11 ± 34 | 39 ± 124 | LC | NT | LC |
| *Canarium subulatum* | 134,750 | 19 | -3 | 9 | 6 | -9 | -62 | 8 ± 11 | -22 ± 36 | LC | LC | NT |
| *Carallia brachiata* | 95,109 | -7 | -58 | -33 | -36 | -73 | -78 | -33 ± 26 | -62 ± 23 | LC | VU | EN |
| *Cardiospermum halicacabum* | 400,027 | 5 | -13 | 31 | 14 | -44 | 38 | 8 ± 22 | 3 ± 42 | LC | LC | LC |
| *Careya arborea* | 105,115 | -3 | -25 | -11 | -28 | -37 | -65 | -13 ± 11 | -43 ± 19 | LC | NT | VU |
| *Caryota maxima* | 216,863 | 15 | -24 | -20 | -23 | 26 | 40 | -9 ± 22 | 14 ± 33 | LC | NT | LC |
| *Casearia grewiifolia* | 153,512 | 29 | 16 | -10 | 6 | 30 | -60 | 11 ± 20 | -8 ± 47 | LC | LC | NT |
| *Cassia fistula* | 99,625 | 128 | 110 | 295 | 389 | 129 | 118 | 178 ± 102 | 212 ± 153 | LC | LC | LC |
| *Castanopsis acuminatissima* | 41,038 | -39 | -71 | -36 | -64 | -89 | -86 | -49 ± 20 | -80 ± 14 | LC | VU | EN |
| *Castanopsis diversifolia* | 101,541 | -21 | -27 | -9 | -48 | -25 | 15 | -19 ± 9 | -20 ± 32 | LC | NT | NT |
| *Castanopsis indica* | 61,836 | -10 | -35 | -5 | 11 | 35 | 19 | -16 ± 16 | 22 ± 12 | LC | NT | LC |
| *Castanopsis pierrei* | 25,225 | -50 | -95 | -57 | -10 | -88 | 9 | -67 ± 24 | -30 ± 52 | NT | EN | VU |
| *Castanopsis purpurea* | 59,023 | -28 | -54 | -17 | -54 | -82 | -13 | -33 ± 19 | -50 ± 35 | LC | VU | VU |
| *Castanopsis tribuloides* | 74,256 | -17 | 5 | 36 | -22 | -55 | -5 | 8 ± 27 | -27 ± 26 | LC | LC | NT |
| *Catunaregam tomentosa* | 143,773 | 45 | -29 | 3 | 45 | 110 | 34 | 6 ± 37 | 63 ± 41 | LC | LC | LC |
| *Celtis timorensis* | 29,863 | -80 | -92 | -92 | -89 | -98 | -100 | -88 ± 7 | -96 ± 6 | NT | CR | CR |
| *Cephalotaxus mannii* | 99,718 | -38 | -52 | 23 | -54 | -93 | -52 | -22 ± 40 | -66 ± 23 | LC | NT | EN |

| | | | | | | | | | | | | |
|---|---|---|---|---|---|---|---|---|---|---|---|---|
| *Champereia manillana* | 55,295 | 166 | 179 | 280 | 267 | 328 | 328 | 208 ± 62 | 308 ± 35 | LC | LC | LC |
| *Chionanthus ramiforus* | 63,481 | -53 | -57 | 57 | -44 | -51 | -54 | -18 ± 65 | -50 ± 5 | LC | NT | VU |
| *Chisocheton cumingianus* | 450,002 | -3 | -1 | -19 | -5 | -76 | -19 | -7 ± 10 | -33 ± 38 | LC | NT | VU |
| *Chisocheton dysoxylifolius* | 4,358 | -42 | -26 | 325 | -20 | -100 | 70 | 86 ± 207 | -17 ± 85 | EN | LC | NT |
| *Chisocheton penduliflorus* | 9,318 | 83 | 210 | 205 | 276 | -41 | 1386 | 166 ± 72 | 540 ± 749 | VU | LC | LC |
| *Choerospondias axillaris* | 74,443 | -11 | -51 | 34 | 18 | -14 | -17 | -10 ± 43 | -4 ± 20 | LC | NT | NT |
| *Christia obcordata* | 328,895 | 4 | -13 | -15 | -10 | -23 | 11 | -8 ± 11 | -8 ± 17 | LC | NT | NT |
| *Chukrasia tabularis* | 120,922 | 16 | 1 | 103 | 82 | 58 | 49 | 40 ± 55 | 63 ± 17 | LC | LC | LC |
| *Cinnamomum parthenoxylon* | 67,560 | -49 | -74 | -67 | -57 | -96 | -89 | -63 ± 13 | -81 ± 21 | LC | EN | CR |
| *Cinnamomum subavenium* | 4,620 | -31 | -35 | 66 | -39 | -78 | -79 | 0 ± 57 | -65 ± 23 | EN | LC | EN |
| *Cipadessa baccifera* | 333,926 | -4 | -6 | -4 | -48 | -30 | 16 | -5 ± 1 | -21 ± 33 | LC | NT | NT |
| *Cleistocalyx operculatus* | 236,656 | -12 | -22 | -34 | -47 | -77 | -56 | -23 ± 11 | -60 ± 15 | LC | NT | EN |
| *Clitoria macrophylla* | 244,489 | -3 | -57 | -7 | -50 | -97 | -13 | -22 ± 30 | -53 ± 42 | LC | NT | EN |
| *Combretum latifolium* | 232,690 | -10 | -26 | 9 | 0 | -12 | 26 | -9 ± 17 | 5 ± 20 | LC | NT | LC |
| *Combretum procursum* | 306,642 | 6 | -8 | 31 | 19 | -62 | 70 | 10 ± 20 | 9 ± 66 | LC | LC | LC |
| *Combretum quadrangulare* | 395,515 | 4 | 13 | 37 | 21 | 4 | 29 | 18 ± 17 | 18 ± 13 | LC | LC | LC |
| *Combretum yunnanense* | 6,343 | 63 | -88 | -50 | 73 | 135 | 524 | -25 ± 78 | 244 ± 245 | VU | NT | LC |
| *Conyza leucantha* | 82,841 | -45 | -85 | -38 | -81 | -98 | -76 | -56 ± 25 | -85 ± 12 | LC | EN | CR |
| *Cornukaempferia aurantiflora* | 53,705 | -80 | -43 | 37 | -93 | -92 | -53 | -29 ± 60 | -80 ± 23 | LC | NT | EN |
| *Corypha lecomtei* | 126,567 | 99 | 197 | 173 | 230 | 223 | 313 | 156 ± 51 | 255 ± 50 | LC | LC | LC |
| *Corypha utan* | 33,274 | -48 | -82 | -53 | -99 | -100 | -100 | -61 ± 18 | -99 ± 1 | LC | EN | CR |
| *Craibiodendron stellatum* | 84,480 | -15 | -25 | 44 | -49 | -17 | 18 | 1 ± 37 | -16 ± 34 | LC | LC | NT |
| *Cratoxylum cochinchinense* | 132,485 | 27 | 8 | -15 | 3 | 26 | -10 | 7 ± 21 | 6 ± 18 | LC | LC | LC |
| *Cratoxylum formosum* | 37,712 | 52 | 124 | 66 | 380 | 235 | -41 | 81 ± 38 | 191 ± 214 | LC | LC | LC |
| *Crinum thaianum* | 862 | 174 | 564 | 141 | 288 | 93 | -92 | 293 ± 236 | 97 ± 190 | EN | LC | LC |
| *Crotalaria alata* | 292,995 | -41 | -49 | -29 | -49 | -98 | -73 | -40 ± 10 | -73 ± 25 | LC | VU | EN |
| *Crotalaria albida* | 98,680 | -16 | -33 | 63 | -52 | -52 | -25 | 4 ± 51 | -43 ± 16 | LC | LC | VU |
| *Crotalaria assamica* | 173,935 | -47 | -78 | -40 | -76 | -95 | -72 | -55 ± 20 | -81 ± 12 | LC | EN | CR |
| *Crotalaria bracteata* | 229,278 | -6 | -22 | 11 | -29 | -84 | 30 | -6 ± 16 | -27 ± 57 | LC | NT | NT |
| *Crotalaria dubia* | 313,297 | -11 | -47 | 1 | -9 | -41 | -39 | -19 ± 25 | -30 ± 18 | LC | NT | VU |
| *Crotalaria kurzii* | 259,646 | -41 | -47 | 6 | -16 | -65 | -14 | -27 ± 29 | -32 ± 29 | LC | NT | VU |
| *Crotalaria lejoloba* | 167,656 | -13 | -47 | 12 | -21 | -63 | -9 | -16 ± 29 | -31 ± 28 | LC | NT | VU |
| *Crotalaria linifolia* | 252,510 | -12 | -20 | 34 | 22 | -47 | -37 | 1 ± 29 | -21 ± 37 | LC | LC | NT |
| *Crotalaria neriifolia* | 343,180 | -11 | -59 | -11 | -22 | -63 | -51 | -27 ± 28 | -46 ± 21 | LC | NT | VU |
| *Crotalaria sessiliflora* | 138,336 | -22 | -61 | -14 | -35 | -75 | -35 | -32 ± 25 | -48 ± 23 | LC | VU | VU |
| *Crotalaria verrucosa* | 404,178 | -3 | 28 | 30 | 0 | 3 | 42 | 18 ± 18 | 15 ± 23 | LC | LC | LC |
| *Croton argyratus* | 9,319 | 82 | 107 | 729 | 301 | 253 | 1523 | 306 ± 366 | 692 ± 719 | VU | LC | LC |
| *Croton persimilis* | 97,968 | -22 | -48 | -52 | -45 | -68 | -89 | -41 ± 16 | -67 ± 22 | LC | VU | EN |
| *Cruddasia insignis* | 151,488 | -44 | -74 | -48 | -65 | -91 | -22 | -55 ± 17 | -59 ± 35 | LC | EN | EN |

| | | | | | | | | | | | | |
|---|---|---|---|---|---|---|---|---|---|---|---|---|
| *Crypteronia paniculata* | 120,527 | 45 | 62 | 55 | 63 | 72 | 13 | 54 ± 8 | 49 ± 32 | LC | LC | LC |
| *Cryptocarya pallens* | 56,062 | -48 | -64 | -62 | -61 | -66 | -76 | -58 ± 9 | -68 ± 8 | LC | EN | EN |
| *Curcuma ecomata* | 38,983 | -53 | -94 | -25 | -87 | -100 | -92 | -57 ± 35 | -93 ± 6 | LC | EN | CR |
| *Curcuma parviflora* | 244,769 | 5 | -35 | 24 | 16 | 12 | -1 | -2 ± 30 | 9 ± 9 | LC | NT | LC |
| *Curcuma roscoeana* | 122,383 | -31 | -43 | 34 | 15 | -58 | 22 | -13 ± 41 | -7 ± 44 | LC | NT | NT |
| *Curcuma rubrobracteata* | 112,683 | -27 | -58 | -23 | -67 | -98 | -34 | -36 ± 19 | -67 ± 32 | LC | VU | EN |
| *Cyathocalyx harmandii* | 133,911 | -4 | -15 | 28 | 11 | 20 | 50 | 3 ± 22 | 27 ± 21 | LC | LC | LC |
| *Cycas macrocarpa* | 51,374 | 40 | 57 | 29 | 50 | 56 | 34 | 42 ± 14 | 47 ± 11 | LC | LC | LC |
| *Cycas nongnoochiae* | 6,676 | 110 | 407 | 800 | 1385 | 738 | 978 | 439 ± 346 | 1034 ± 327 | VU | LC | LC |
| *Cycas pectinata* | 140,461 | 77 | 51 | 76 | 31 | 84 | 11 | 68 ± 14 | 42 ± 38 | LC | LC | LC |
| *Cycas petrae* | 11,404 | 10 | -100 | -100 | -43 | 106 | 10 | -63 ± 64 | 24 ± 76 | VU | EN | LC |
| *Cycas pranburiensis* | 1,624 | -40 | -70 | -100 | -49 | 1 | -100 | -70 ± 30 | -49 ± 51 | EN | EN | VU |
| *Cycas siamensis* | 98,503 | -51 | -72 | -63 | -90 | -92 | -94 | -62 ± 11 | -92 ± 2 | LC | EN | CR |
| *Cycas simplicipinna* | 21,587 | -17 | -100 | -100 | -64 | 19 | 7 | -72 ± 48 | -13 ± 45 | NT | EN | NT |
| *Cycas tansachana* | 74,891 | 158 | 253 | 284 | 298 | 562 | 498 | 232 ± 66 | 453 ± 137 | LC | LC | LC |
| *Dacrycarpus imbricatus* | 34,493 | -62 | -93 | -92 | -95 | -93 | -99 | -82 ± 18 | -96 ± 3 | LC | CR | CR |
| *Dacrydium elatum* | 21,868 | -63 | -95 | -97 | -93 | -52 | -99 | -85 ± 19 | -81 ± 26 | NT | CR | CR |
| *Dalbergia cana* | 338,528 | -11 | -33 | 1 | -10 | -21 | 31 | -14 ± 17 | 0 ± 27 | LC | NT | LC |
| *Dalbergia cochinchinensis* | 44,734 | 15 | -79 | -78 | -71 | -99 | -97 | -47 ± 54 | -89 ± 16 | LC | VU | CR |
| *Dalbergia cultrata* | 128,405 | 26 | 40 | 44 | -2 | -12 | -3 | 36 ± 9 | -6 ± 5 | LC | LC | NT |
| *Dalbergia foliacea* | 325,349 | -42 | -63 | -41 | -67 | -92 | -63 | -49 ± 12 | -74 ± 15 | LC | VU | EN |
| *Dalbergia lanceolaria* | 168,239 | -37 | -44 | 73 | -43 | -39 | -93 | -3 ± 66 | -58 ± 30 | LC | NT | EN |
| *Dalbergia nigrescens* | 254,524 | -17 | -73 | -3 | -52 | -30 | 33 | -31 ± 37 | -16 ± 44 | LC | VU | NT |
| *Dalbergia oliveri* | 127,871 | 13 | 44 | 18 | 27 | 52 | 10 | 25 ± 16 | 30 ± 21 | LC | LC | LC |
| *Dalbergia rimosa* | 138,638 | -51 | -54 | -48 | -86 | -98 | -86 | -51 ± 3 | -90 ± 7 | LC | EN | CR |
| *Dalbergia stipulacea* | 107,304 | -50 | -82 | -63 | -84 | -84 | -90 | -65 ± 16 | -86 ± 4 | LC | EN | CR |
| *Dalbergia velutina* | 220,642 | -10 | -4 | 14 | 33 | 31 | -30 | 0 ± 12 | 11 ± 36 | LC | LC | LC |
| *Dehaasia kurzii* | 11,048 | -50 | -72 | -40 | -79 | -99 | -85 | -54 ± 17 | -88 ± 10 | VU | EN | CR |
| *Derris scandens* | 396,598 | 27 | 29 | 26 | 42 | 45 | 20 | 27 ± 2 | 36 ± 14 | LC | LC | LC |
| *Derris trifoliata* | 67,325 | 113 | 184 | 326 | 222 | 480 | 500 | 208 ± 109 | 401 ± 155 | LC | LC | LC |
| *Desmodium heterocarpon* | 175,738 | -29 | -18 | 42 | -71 | -83 | -89 | -2 ± 38 | -81 ± 9 | LC | NT | CR |
| *Desmodium laxiflorum* | 144,905 | -27 | -37 | 26 | -27 | -82 | -9 | -13 ± 34 | -39 ± 38 | LC | NT | VU |
| *Desmodium megaphyllum* | 131,370 | -10 | 14 | 9 | 12 | -51 | 60 | 4 ± 12 | 7 ± 56 | LC | LC | LC |
| *Desmodium motorium* | 175,689 | -18 | -45 | 41 | 14 | -69 | 74 | -8 ± 44 | 6 ± 72 | LC | NT | LC |
| *Desmodium oblongum* | 83,667 | -23 | -51 | 56 | -23 | -67 | 131 | -6 ± 56 | 14 ± 104 | LC | NT | LC |
| *Desmodium renifolium* | 185,468 | -32 | -88 | -65 | -79 | -79 | -95 | -62 ± 28 | -84 ± 10 | LC | EN | CR |
| *Desmodium triangulare* | 356,210 | -1 | 19 | 9 | 8 | -14 | 28 | 9 ± 10 | 7 ± 21 | LC | LC | LC |
| *Desmodium triflorum* | 354,181 | -9 | -39 | -28 | -39 | -52 | -39 | -26 ± 15 | -43 ± 8 | LC | NT | VU |
| *Desmodium velutinum* | 352,749 | 4 | -17 | 1 | 0 | -31 | 19 | -4 ± 11 | -4 ± 25 | LC | NT | NT |

| | | | | | | | | | | | | |
|---|---|---|---|---|---|---|---|---|---|---|---|---|
| *Dialium cochinchinense* | 61,487 | 54 | 52 | -28 | 81 | -29 | -50 | 26 ± 47 | 1 ± 70 | LC | LC | LC |
| *Dillenia obovata* | 115,317 | -9 | -29 | -5 | -50 | -58 | -65 | -14 ± 13 | -57 ± 7 | LC | NT | EN |
| *Dimocarpus fumatus* | 45,631 | 10 | 76 | 141 | 122 | -15 | 70 | 75 ± 65 | 59 ± 69 | LC | LC | LC |
| *Dimocarpus longan* | 308,554 | -38 | -62 | -40 | -78 | -87 | -83 | -47 ± 14 | -83 ± 4 | LC | VU | CR |
| *Dioecrescis erythroclada* | 128,036 | 1 | -16 | 21 | 2 | 7 | -91 | 2 ± 19 | -27 ± 56 | LC | LC | NT |
| *Diospyros andamanica* | 3,990 | -34 | -92 | -69 | -93 | -100 | -62 | -65 ± 29 | -85 ± 20 | EN | EN | CR |
| *Diospyros bejaudii* | 37,327 | 29 | 7 | 118 | 45 | 10 | 72 | 51 ± 59 | 42 ± 31 | LC | LC | LC |
| *Diospyros buxifolia* | 11,835 | -37 | 5 | 14 | 80 | -93 | 215 | -6 ± 27 | 67 ± 155 | VU | NT | LC |
| *Diospyros cauliflora* | 74,976 | 7 | -23 | 50 | 25 | -72 | -4 | 11 ± 36 | -17 ± 50 | LC | LC | NT |
| *Diospyros dasyphylla* | 69,241 | -18 | -63 | 8 | -51 | -75 | -20 | -24 ± 36 | -49 ± 28 | LC | NT | VU |
| *Diospyros decandra* | 37,645 | -38 | -85 | -51 | -91 | -98 | -100 | -58 ± 24 | -96 ± 5 | LC | EN | CR |
| *Diospyros glandulosa* | 56,825 | -59 | -77 | -47 | -76 | -99 | -91 | -61 ± 15 | -89 ± 12 | LC | EN | CR |
| *Diospyros insidiosa* | 4,980 | -56 | -80 | -13 | -27 | -97 | -100 | -50 ± 34 | -74 ± 41 | EN | VU | EN |
| *Diospyros malabarica* | 122,134 | -1 | 71 | 41 | 64 | 34 | 49 | 37 ± 36 | 49 ± 15 | LC | LC | LC |
| *Diospyros mollis* | 128,075 | 45 | 100 | 35 | 61 | 48 | -10 | 60 ± 35 | 33 ± 38 | LC | LC | LC |
| *Diospyros thaiensis* | 134,765 | 51 | 107 | 136 | 132 | 119 | 156 | 98 ± 43 | 136 ± 19 | LC | LC | LC |
| *Diospyros transitoria* | 20,664 | -51 | -75 | -72 | -91 | -95 | -100 | -66 ± 13 | -96 ± 4 | NT | EN | CR |
| *Diospyros wallichii* | 3,714 | -57 | -95 | -91 | -85 | -100 | -100 | -81 ± 21 | -95 ± 8 | EN | CR | CR |
| *Diospyros winitii* | 96,266 | -47 | -70 | -47 | -69 | -95 | -92 | -55 ± 13 | -85 ± 14 | LC | EN | CR |
| *Diplycosia cf. heterophylla* | 741 | -44 | -99 | -98 | -100 | -100 | -100 | -80 ± 31 | -100 ± 0 | EN | EN | EX |
| *Dipterocarpus alatus* | 129,144 | 46 | 162 | 176 | 182 | 187 | 320 | 128 ± 71 | 230 ± 78 | LC | LC | LC |
| *Dipterocarpus baudii* | 14,407 | -37 | -73 | -46 | -79 | -96 | -86 | -52 ± 19 | -87 ± 8 | VU | EN | CR |
| *Dipterocarpus costatus* | 105,955 | -22 | -55 | 0 | -50 | -66 | -76 | -26 ± 28 | -64 ± 13 | LC | NT | EN |
| *Dipterocarpus dyeri* | 27,685 | -12 | -32 | -29 | -13 | -21 | -95 | -25 ± 11 | -43 ± 45 | NT | NT | VU |
| *Dipterocarpus gracilis* | 6,144 | -56 | -33 | -24 | -58 | -89 | -81 | -38 ± 16 | -76 ± 16 | VU | VU | EN |
| *Dipterocarpus grandiflorus* | 16,835 | -52 | -59 | -28 | -89 | -99 | -89 | -47 ± 17 | -92 ± 6 | VU | VU | CR |
| *Dipterocarpus intricatus* | 147,858 | 50 | 58 | 90 | 98 | 74 | 107 | 66 ± 21 | 93 ± 17 | LC | LC | LC |
| *Dipterocarpus kerrii* | 22,275 | -27 | 3 | 75 | 139 | 117 | -79 | 17 ± 52 | 59 ± 120 | NT | LC | LC |
| *Dipterocarpus obtusifolius* | 142,279 | 82 | 115 | 147 | 27 | 134 | 57 | 115 ± 33 | 73 ± 55 | LC | LC | LC |
| *Dipterocarpus retusus* | 296,446 | -4 | -38 | -29 | -78 | -89 | -60 | -24 ± 18 | -76 ± 15 | LC | NT | EN |
| *Dipterocarpus tuberculatus* | 143,972 | 17 | 16 | 10 | 7 | 23 | -15 | 15 ± 4 | 5 ± 19 | LC | LC | LC |
| *Dipterocarpus turbinatus* | 74,542 | -7 | -43 | 12 | -20 | -78 | -46 | -13 ± 28 | -48 ± 29 | LC | NT | VU |
| *Dolichandrone serrulata* | 97,495 | 5 | 5 | 50 | -20 | -2 | 11 | 20 ± 26 | -4 ± 15 | LC | LC | NT |
| *Dracontomelon dao* | 56,546 | -13 | -34 | 94 | -7 | -48 | 61 | 15 ± 69 | 2 ± 55 | LC | LC | LC |
| *Drypetes hoaensis* | 35,598 | -12 | -54 | -12 | -43 | -60 | -74 | -26 ± 24 | -59 ± 15 | LC | NT | EN |
| *Dunbaria bella* | 228,623 | -6 | -40 | -21 | -27 | -60 | -44 | -22 ± 18 | -44 ± 16 | LC | NT | VU |
| *Dunbaria longeracemosa* | 80,963 | -41 | -81 | -7 | -72 | -99 | -33 | -43 ± 37 | -68 ± 33 | LC | VU | EN |
| *Durio lowianus* | 7,475 | -33 | 12 | -46 | -9 | -53 | -98 | -22 ± 30 | -53 ± 44 | VU | NT | EN |
| *Durio mansoni* | 94,456 | -26 | -38 | -76 | -74 | -53 | -90 | -47 ± 26 | -72 ± 18 | LC | VU | EN |

| | | | | | | | | | | | | |
|---|---|---|---|---|---|---|---|---|---|---|---|---|
| *Dysolobium grande* | 232,189 | 2 | -16 | 27 | -19 | -51 | 31 | 4 ± 21 | -13 ± 41 | LC | LC | NT |
| *Dysoxylum cyrtobotryum* | 84,860 | -28 | 46 | 69 | -6 | -44 | 3 | 29 ± 51 | -16 ± 25 | LC | LC | NT |
| *Eclipta prostrata* | 361,687 | -8 | -23 | 0 | -20 | -21 | 13 | -10 ± 12 | -9 ± 19 | LC | NT | NT |
| *Elaeocarpus_floribundus* | 80,168 | -35 | -53 | -26 | -82 | -63 | -81 | -38 ± 14 | -75 ± 11 | LC | VU | EN |
| *Elaeocarpus_grandiflorus* | 413,418 | -14 | -5 | -2 | 0 | -32 | 18 | -7 ± 6 | -4 ± 25 | LC | NT | NT |
| *Elaeocarpus_petiolatus* | 60,542 | 4 | 39 | -19 | -4 | 38 | 23 | 8 ± 29 | 19 ± 21 | LC | LC | LC |
| *Elaeocarpus_robustus* | 325,912 | 0 | 0 | -2 | -6 | -12 | 2 | -1 ± 1 | -5 ± 7 | LC | NT | NT |
| *Elaeocarpus_tectorius* | 195,633 | -23 | -64 | -46 | -83 | -74 | -77 | -45 ± 21 | -78 ± 5 | LC | VU | EN |
| *Elephantopus scaber* | 305,672 | -21 | -19 | -6 | -46 | -20 | -81 | -15 ± 8 | -49 ± 31 | LC | NT | VU |
| *Elettariopsis curtisii* | 8,615 | 162 | 226 | 97 | 158 | 254 | 128 | 162 ± 64 | 180 ± 66 | VU | LC | LC |
| *Ellipanthus tomentosus* | 101,363 | 61 | 51 | 17 | 23 | 25 | 53 | 43 ± 23 | 34 ± 17 | LC | LC | LC |
| *Endocomia macrocoma* | 276,070 | -4 | 41 | 48 | 77 | 30 | 58 | 28 ± 28 | 55 ± 24 | LC | LC | LC |
| *Endosamara racemosa* | 190,320 | -25 | -68 | 15 | -21 | -37 | 73 | -26 ± 42 | 5 ± 59 | LC | NT | LC |
| *Engelhardtia spicata* | 6,771 | -36 | -93 | -91 | -80 | -93 | -100 | -73 ± 32 | -91 ± 10 | VU | EN | CR |
| *Entada rheedii* | 74,897 | -40 | -69 | -18 | -57 | -97 | -85 | -42 ± 26 | -80 ± 20 | LC | VU | EN |
| *Eriolaena candollei* | 104,798 | -5 | 8 | -6 | -4 | -46 | -68 | -1 ± 8 | -40 ± 32 | LC | NT | VU |
| *Eriosema chinense* | 284,061 | -39 | -75 | -17 | -74 | -95 | -82 | -44 ± 29 | -83 ± 11 | LC | VU | CR |
| *Erythrina stricta* | 256,352 | -19 | -55 | -11 | -44 | -79 | -30 | -28 ± 23 | -51 ± 26 | LC | NT | EN |
| *Etlingera corneri* | 11,524 | -2 | 36 | -2 | -18 | -77 | -71 | 11 ± 22 | -55 ± 32 | VU | LC | EN |
| *Etlingera littoralis* | 111,055 | -17 | -40 | -14 | -36 | -55 | -28 | -24 ± 14 | -40 ± 14 | LC | NT | VU |
| *Etlingera maingayi* | 21,626 | 67 | 27 | 151 | 168 | -28 | -35 | 82 ± 63 | 35 ± 115 | NT | LC | LC |
| *Etlingera venusta* | 517 | 35 | 677 | -37 | 524 | 340 | -100 | 225 ± 393 | 255 ± 321 | EN | LC | LC |
| *Eurya acuminata* | 212,396 | -12 | -67 | -15 | -50 | -69 | -23 | -31 ± 31 | -47 ± 23 | LC | VU | VU |
| *Eurya nitida* | 296,966 | -28 | -64 | -45 | -70 | -76 | -88 | -45 ± 18 | -78 ± 9 | LC | VU | EN |
| *Eurycoma longifolia* | 35,767 | -21 | -56 | -1 | -51 | -51 | -35 | -26 ± 28 | -46 ± 10 | LC | NT | VU |
| *Fagraea fragrans* | 39,922 | -24 | -26 | -14 | -34 | -14 | -80 | -21 ± 7 | -42 ± 34 | LC | NT | VU |
| *Falconeria insignis* | 74,066 | -22 | -25 | 26 | -11 | -41 | 32 | -7 ± 29 | -7 ± 37 | LC | NT | NT |
| *Ficus altissima* | 81,040 | -38 | -53 | 30 | -49 | -91 | -60 | -20 ± 44 | -67 ± 22 | LC | NT | EN |
| *Ficus anastomosans* | 316,366 | 5 | 9 | 40 | 17 | 31 | 36 | 18 ± 19 | 28 ± 10 | LC | LC | LC |
| *Ficus annulata* | 310,423 | -2 | -9 | 0 | -21 | -21 | -4 | -4 ± 5 | -15 ± 10 | LC | NT | NT |
| *Ficus auriculata* | 135,434 | -27 | -76 | 3 | -55 | -96 | -87 | -33 ± 39 | -79 ± 21 | LC | VU | EN |
| *Ficus benjamina* | 287,331 | -5 | -52 | 6 | -20 | -22 | 4 | -17 ± 31 | -12 ± 14 | LC | NT | NT |
| *Ficus chartacea* | 135,205 | -23 | -61 | -70 | -91 | -94 | -97 | -51 ± 25 | -94 ± 3 | LC | EN | CR |
| *Ficus concinna* | 367,135 | 16 | -32 | 35 | -1 | -59 | 35 | 6 ± 35 | -8 ± 47 | LC | LC | NT |
| *Ficus curtipes* | 286,726 | -28 | -45 | -20 | -53 | -95 | -44 | -31 ± 13 | -64 ± 27 | LC | VU | EN |
| *Ficus fistulosa* | 139,753 | -51 | -75 | -44 | -71 | -94 | -92 | -57 ± 16 | -86 ± 13 | LC | EN | CR |
| *Ficus glaberrima* | 200,928 | 14 | 95 | 113 | 27 | 13 | 16 | 74 ± 53 | 19 ± 8 | LC | LC | LC |
| *Ficus heteropleura* | 94,160 | -4 | 71 | 108 | 64 | 95 | 53 | 58 ± 57 | 71 ± 22 | LC | LC | LC |
| *Ficus hirta* | 117,438 | -56 | -94 | -86 | -91 | -97 | -99 | -79 ± 20 | -96 ± 4 | LC | EN | CR |

| | | | | | | | | | | | | |
|---|---|---|---|---|---|---|---|---|---|---|---|---|
| *Ficus hispida* | 296,333 | -12 | -58 | -42 | -40 | -86 | -66 | -37 ± 23 | -64 ± 23 | LC | VU | EN |
| *Ficus ischnopoda* | 215,142 | 15 | 19 | 39 | 5 | 55 | 95 | 25 ± 13 | 52 ± 45 | LC | LC | LC |
| *Ficus lepicarpa* | 414,949 | 33 | -59 | -85 | -22 | 47 | -29 | -37 ± 62 | -1 ± 42 | LC | VU | NT |
| *Ficus microcarpa* | 172,016 | 57 | 60 | 71 | 13 | 41 | 24 | 63 ± 7 | 26 ± 14 | LC | LC | LC |
| *Ficus parietalis* | 284,477 | -36 | -53 | -12 | -35 | -70 | -52 | -34 ± 21 | -53 ± 17 | LC | VU | EN |
| *Ficus punctata* | 212,273 | -10 | -50 | 0 | -24 | -68 | -24 | -20 ± 26 | -39 ± 26 | LC | NT | VU |
| *Ficus racemosa* | 171,642 | -12 | -32 | -31 | -24 | -40 | -59 | -25 ± 11 | -41 ± 18 | LC | NT | VU |
| *Ficus subincisa* | 253,944 | -13 | -25 | 23 | 17 | -56 | -17 | -5 ± 25 | -19 ± 37 | LC | NT | NT |
| *Ficus subpisocarpa* | 410,998 | 11 | -11 | 5 | 11 | 11 | 19 | 2 ± 11 | 13 ± 5 | LC | LC | LC |
| *Ficus subulata* | 166,348 | 23 | 4 | 19 | 109 | 4 | 119 | 15 ± 10 | 78 ± 64 | LC | LC | LC |
| *Ficus talbotii* | 292,501 | -12 | -13 | 1 | -23 | -51 | 19 | -8 ± 7 | -18 ± 35 | LC | NT | NT |
| *Ficus tinctoria* | 406,254 | -1 | -2 | 13 | 1 | 16 | 15 | 3 ± 9 | 11 ± 8 | LC | LC | LC |
| *Ficus variegata* | 450,340 | -11 | -37 | -5 | -14 | -30 | 3 | -18 ± 17 | -14 ± 16 | LC | NT | NT |
| *Ficus virens* | 268,950 | -35 | -78 | -33 | -45 | -88 | -78 | -48 ± 26 | -71 ± 22 | LC | VU | EN |
| *Firmiana colorata* | 86,665 | -2 | 21 | 55 | 2 | 49 | 34 | 25 ± 29 | 29 ± 24 | LC | LC | LC |
| *Flemingia lineata* | 206,420 | -18 | -23 | -17 | -28 | -80 | -14 | -19 ± 3 | -41 ± 35 | LC | NT | VU |
| *Flemingia sootepensis* | 130,768 | -39 | -65 | -9 | -59 | -89 | -69 | -37 ± 28 | -72 ± 15 | LC | VU | EN |
| *Flemingia strobilifera* | 285,654 | -6 | 0 | 26 | 16 | -19 | 32 | 6 ± 17 | 10 ± 26 | LC | LC | LC |
| *Garcinia cowa* | 108,059 | 19 | -32 | 9 | -1 | -6 | -45 | -1 ± 27 | -18 ± 24 | LC | NT | NT |
| *Garcinia speciosa* | 82,066 | 13 | -53 | -19 | -36 | -56 | -76 | -20 ± 33 | -56 ± 20 | LC | NT | EN |
| *Gardenia sootepensis* | 115,200 | 20 | 68 | 61 | 34 | 35 | 27 | 50 ± 26 | 32 ± 4 | LC | LC | LC |
| *Garuga floribunda* | 17,124 | 32 | 300 | 806 | 186 | 406 | 363 | 379 ± 393 | 319 ± 116 | VU | LC | LC |
| *Garuga pinnata* | 148,604 | 25 | 55 | 77 | 27 | 54 | 33 | 52 ± 26 | 38 ± 14 | LC | LC | LC |
| *Geostachys angustifolia* | 3,647 | -57 | -85 | 10 | -53 | -98 | -88 | -44 ± 49 | -80 ± 23 | EN | VU | EN |
| *Geostachys smitinandii* | 40,442 | -37 | -78 | -21 | -85 | -65 | -98 | -46 ± 29 | -82 ± 16 | LC | VU | CR |
| *Globba kerrii* | 89,644 | -54 | -72 | -24 | -87 | -100 | -97 | -50 ± 25 | -95 ± 7 | LC | VU | CR |
| *Globba nuda* | 73,768 | -38 | -74 | -22 | -82 | -98 | -52 | -45 ± 27 | -77 ± 23 | LC | VU | EN |
| *Globba obscura* | 147,631 | -47 | -70 | -22 | -68 | -100 | -74 | -46 ± 24 | -80 ± 17 | LC | VU | EN |
| *Globba purpurascens* | 59,215 | -35 | -39 | 78 | -67 | -94 | -43 | 1 ± 67 | -68 ± 25 | LC | LC | EN |
| *Globba reflexa* | 40,205 | -60 | -10 | 144 | -66 | -62 | -66 | 25 ± 107 | -64 ± 2 | LC | LC | EN |
| *Globba schomburgkii* | 256,970 | -14 | -67 | -8 | -51 | -78 | -44 | -30 ± 32 | -58 ± 18 | LC | VU | EN |
| *Globba siamensis* | 62,590 | -13 | -63 | -88 | -82 | -93 | -99 | -55 ± 38 | -91 ± 8 | LC | EN | CR |
| *Globba villosula* | 489,821 | 0 | -20 | 5 | -2 | -19 | 9 | -5 ± 13 | -4 ± 14 | LC | NT | NT |
| *Globba winitii* | 142,764 | -15 | 9 | 87 | -32 | -45 | 61 | 27 ± 54 | -5 ± 58 | LC | LC | NT |
| *Gluta glabra* | 45,078 | -8 | -31 | 66 | -23 | -49 | 50 | 9 ± 51 | -7 ± 52 | LC | LC | NT |
| *Gluta laccifera* | 370,168 | 1 | 18 | 23 | 17 | -13 | 32 | 14 ± 11 | 12 ± 23 | LC | LC | LC |
| *Gluta usitata* | 142,091 | 16 | 18 | 0 | 20 | 12 | -25 | 11 ± 10 | 2 ± 24 | LC | LC | LC |
| *Gmelina arborea* | 85,041 | -38 | -51 | -31 | -74 | -59 | -81 | -40 ± 10 | -71 ± 11 | LC | VU | EN |
| *Goniothalamus laoticus* | 93,207 | 8 | -50 | -41 | -16 | -17 | -65 | -28 ± 31 | -33 ± 28 | LC | NT | VU |

| | | | | | | | | | | | | |
|---|---|---|---|---|---|---|---|---|---|---|---|---|
| *Gordonia axillaris* | 94,218 | 50 | -40 | 13 | 14 | 32 | -46 | 8 ± 45 | 0 ± 41 | LC | LC | LC |
| *Gordonia dalglieshiana* | 33,699 | -38 | -84 | -42 | -88 | -93 | -90 | -55 ± 26 | -91 ± 2 | LC | EN | CR |
| *Gynura pseudochina* | 204,269 | -37 | -54 | 2 | -36 | -96 | -59 | -29 ± 29 | -63 ± 30 | LC | NT | EN |
| *Haldina cordifolia* | 87,604 | 25 | 83 | 97 | 71 | 104 | 8 | 68 ± 38 | 61 ± 49 | LC | LC | LC |
| *Harpullia arborea* | 237,828 | -35 | -49 | -28 | -67 | -73 | -60 | -38 ± 10 | -66 ± 6 | LC | VU | EN |
| *Harpullia cupanioides* | 229,390 | -19 | -55 | -26 | -64 | -56 | -71 | -33 ± 19 | -64 ± 7 | LC | VU | EN |
| *Hedychium ellipticum* | 15,618 | -47 | -82 | -49 | -73 | -90 | -96 | -59 ± 20 | -86 ± 12 | VU | EN | CR |
| *Hedychium tomentosum* | 75,647 | -50 | -84 | -22 | -72 | -99 | -88 | -52 ± 31 | -86 ± 13 | LC | EN | CR |
| *Hedychium villosum* | 27,633 | 72 | -72 | 14 | 82 | 207 | 409 | 5 ± 73 | 233 ± 165 | NT | LC | LC |
| *Helicia nilagirica* | 49,870 | 0 | -15 | -44 | 5 | 5 | -71 | -20 ± 23 | -21 ± 44 | LC | NT | NT |
| *Heritiera javanica* | 76,342 | 10 | -4 | -6 | 3 | -4 | -45 | 0 ± 9 | -16 ± 26 | LC | LC | NT |
| *Heteropanax fragrans* | 40,172 | -21 | 32 | 72 | -26 | -10 | 3 | 28 ± 47 | -11 ± 14 | LC | LC | NT |
| *Heynea trijuga* | 67,527 | -36 | -75 | -7 | -74 | -96 | -47 | -39 ± 34 | -72 ± 24 | LC | VU | EN |
| *Holoptelea integrifolia* | 155,312 | 11 | 29 | 60 | 34 | 44 | 65 | 33 ± 24 | 47 ± 15 | LC | LC | LC |
| *Homalium tomentosum* | 156,874 | 30 | -39 | -5 | -23 | 17 | -48 | -5 ± 35 | -18 ± 33 | LC | NT | NT |
| *Hopea ferrea* | 86,865 | -2 | -20 | 41 | -2 | -31 | -71 | 6 ± 32 | -34 ± 34 | LC | LC | VU |
| *Hopea helferi* | 9,871 | 201 | 354 | 234 | 357 | 538 | 119 | 263 ± 80 | 338 ± 210 | VU | LC | LC |
| *Hopea oblongifolia* | 1,854 | -65 | -9 | -89 | -77 | -97 | -100 | -54 ± 41 | -91 ± 13 | EN | EN | CR |
| *Hopea odorata* | 128,304 | -16 | -47 | -8 | -37 | -58 | 20 | -24 ± 20 | -25 ± 40 | LC | NT | NT |
| *Hopea pierrei* | 24,050 | -30 | -43 | -79 | -59 | -97 | -98 | -51 ± 25 | -85 ± 22 | NT | EN | CR |
| *Hopea recopei* | 42,314 | -13 | -7 | -90 | 86 | -36 | -83 | -37 ± 46 | -11 ± 87 | LC | VU | NT |
| *Hopea thorelii* | 1,404 | -99 | -100 | 279 | -87 | -100 | -45 | 27 ± 218 | -77 ± 29 | EN | LC | EN |
| *Horsfieldia amygdalina* | 111,613 | 15 | 3 | 101 | -20 | 36 | 52 | 40 ± 54 | 23 ± 38 | LC | LC | LC |
| *Horsfieldia glabra* | 96,223 | 8 | 5 | 59 | 31 | 4 | 31 | 24 ± 30 | 22 ± 16 | LC | LC | LC |
| *Horsfieldia irya* | 186,589 | 82 | 102 | 83 | 144 | 43 | 104 | 89 ± 11 | 97 ± 51 | LC | LC | LC |
| *Hydnocarpus anthelminthicus* | 391,509 | -1 | -37 | -11 | -36 | -39 | -27 | -17 ± 19 | -34 ± 6 | LC | NT | VU |
| *Hydnocarpus ilicifolia* | 41,338 | 23 | -55 | -32 | -16 | -66 | -94 | -21 ± 40 | -59 ± 39 | LC | NT | EN |
| *Ilex cymosa* | 78,563 | 7 | 5 | 9 | -25 | 11 | 33 | 7 ± 2 | 6 ± 29 | LC | LC | LC |
| *Ilex micrococca* | 64,290 | -46 | -93 | -28 | -81 | -100 | -84 | -56 ± 33 | -88 ± 10 | LC | EN | CR |
| *Ilex umbellata* | 222,755 | -8 | -40 | -14 | -24 | -80 | -29 | -21 ± 17 | -44 ± 31 | LC | NT | VU |
| *Ilex wallichii* | 74,314 | 34 | -13 | -9 | 128 | -13 | 0 | 4 ± 26 | 38 ± 78 | LC | LC | LC |
| *Indigofera caloneura* | 102,378 | -33 | -67 | 41 | -31 | -90 | 19 | -20 ± 55 | -34 ± 54 | LC | NT | VU |
| *Indigofera cassioides* | 191,520 | -10 | -42 | 16 | 6 | -51 | 46 | -12 ± 29 | 0 ± 49 | LC | NT | LC |
| *Indigofera dosua* | 72,276 | -34 | -67 | 9 | -53 | -94 | 47 | -31 ± 38 | -33 ± 73 | LC | VU | VU |
| *Indigofera lacei* | 100,987 | -17 | -41 | 45 | -7 | -36 | 93 | -5 ± 45 | 17 ± 68 | LC | NT | LC |
| *Indigofera squalida* | 331,833 | -9 | -17 | -7 | -31 | -69 | 5 | -11 ± 5 | -32 ± 37 | LC | NT | VU |
| *Intsia palembanica* | 69,760 | -17 | -60 | -30 | -87 | -93 | -86 | -36 ± 22 | -89 ± 4 | LC | VU | CR |
| *Inula cappa* | 81,894 | -26 | -58 | 41 | -39 | -74 | 15 | -14 ± 50 | -32 ± 45 | LC | NT | VU |
| *Inula indica* | 129,486 | -39 | -53 | 50 | -32 | -37 | 107 | -14 ± 56 | 13 ± 82 | LC | NT | LC |

| | | | | | | | | | | | | |
|---|---|---|---|---|---|---|---|---|---|---|---|---|
| *Inula nervosa* | 100,987 | -20 | -77 | -4 | -42 | -85 | 68 | -34 ± 38 | -20 ± 79 | LC | VU | NT |
| *Irvingia malayana* | 104,594 | 23 | 25 | 40 | 32 | 23 | -23 | 29 ± 9 | 11 ± 29 | LC | LC | LC |
| *Kaempferia elegans* | 75,661 | -35 | -28 | 98 | -67 | -99 | 9 | 12 ± 75 | -52 ± 55 | LC | LC | EN |
| *Kaempferia larsenii* | 3,160 | 42 | 231 | 513 | 632 | 403 | 324 | 262 ± 237 | 453 ± 160 | EN | LC | LC |
| *Kaempferia rotunda* | 103,388 | -28 | -45 | 52 | -23 | -89 | -32 | -7 ± 52 | -48 ± 36 | LC | NT | VU |
| *Kaempferia siamensis* | 56,999 | -23 | -65 | 90 | -35 | -71 | 227 | 1 ± 80 | 40 ± 163 | LC | LC | LC |
| *Kaempferia spoliata* | 53,005 | -55 | -61 | 18 | -79 | -96 | 41 | -33 ± 44 | -45 ± 75 | LC | VU | VU |
| *Kailarsenia lineata* | 8,298 | 56 | 130 | -63 | -42 | -88 | -100 | 41 ± 97 | -77 ± 31 | VU | LC | EN |
| *Knema andamanica* | 6,304 | 113 | 177 | 409 | 609 | 128 | 29 | 233 ± 156 | 255 ± 310 | VU | LC | LC |
| *Knema austrosiamensis* | 14,301 | -50 | -76 | -17 | -43 | -94 | -73 | -48 ± 30 | -70 ± 26 | VU | VU | EN |
| *Knema elegans* | 201,212 | -17 | 12 | 25 | 17 | -23 | 57 | 7 ± 22 | 17 ± 40 | LC | LC | LC |
| *Knema globularia* | 105,320 | 30 | 56 | 47 | 17 | 34 | -16 | 44 ± 13 | 12 ± 25 | LC | LC | LC |
| *Knema globulatericia* | 52,194 | -73 | -90 | -89 | -94 | -100 | -99 | -84 ± 10 | -97 ± 3 | LC | CR | CR |
| *Knema laurina* | 43,903 | -40 | -45 | -48 | -57 | -27 | -55 | -44 ± 4 | -46 ± 17 | LC | VU | VU |
| *Knema lenta* | 332,606 | 7 | -13 | -10 | -2 | -14 | -27 | -5 ± 11 | -14 ± 12 | LC | NT | NT |
| *Knema tenuinervia* | 229,119 | -9 | -1 | 17 | -30 | -64 | -25 | 2 ± 13 | -40 ± 21 | LC | LC | VU |
| *Lagerstroemia calyculata* | 77,289 | 38 | 15 | 38 | 43 | 9 | 18 | 31 ± 13 | 24 ± 18 | LC | LC | LC |
| *Lagerstroemia loudonii* | 82,932 | 36 | -13 | 50 | 43 | -13 | 143 | 24 ± 33 | 58 ± 79 | LC | LC | LC |
| *Lagerstroemia macrocarpa* | 135,577 | 15 | 30 | 36 | 6 | 32 | 46 | 27 ± 11 | 28 ± 20 | LC | LC | LC |
| *Laggera alata* | 110,557 | -27 | -50 | 7 | -42 | -93 | 23 | -24 ± 29 | -37 ± 58 | LC | NT | VU |
| *Lepisanthes rubiginosa* | 258,179 | 56 | 95 | 69 | 25 | 60 | 52 | 73 ± 20 | 46 ± 18 | LC | LC | LC |
| *Lepisanthes tetraphylla* | 178,756 | -29 | -57 | -51 | -87 | -81 | -86 | -46 ± 15 | -85 ± 3 | LC | VU | CR |
| *Lithocarpus dealbatus* | 415,606 | -23 | -76 | -23 | -35 | -82 | -42 | -41 ± 31 | -53 ± 26 | LC | VU | EN |
| *Lithocarpus elegans* | 97,548 | -37 | -52 | -61 | -54 | -93 | -66 | -50 ± 12 | -71 ± 20 | LC | VU | EN |
| *Lithocarpus fenestratus* | 116,089 | -43 | -35 | -4 | -61 | -79 | -70 | -27 ± 21 | -70 ± 9 | LC | NT | EN |
| *Lithocarpus polystachyus* | 85,311 | -19 | -26 | -15 | -59 | -62 | -46 | -20 ± 5 | -56 ± 8 | LC | NT | EN |
| *Lithocarpus sootepensis* | 294,605 | 0 | -21 | 13 | -1 | -20 | 31 | -3 ± 17 | 4 ± 26 | LC | NT | LC |
| *Lithocarpus truncatus* | 42,251 | -60 | -49 | -24 | -85 | -95 | -88 | -45 ± 18 | -89 ± 5 | LC | VU | CR |
| *Litsea cubeba* | 145,363 | -37 | -52 | -42 | -67 | -88 | -71 | -44 ± 8 | -75 ± 11 | LC | VU | EN |
| *Litsea grandis* | 8,874 | -51 | -64 | -45 | -64 | -96 | -82 | -53 ± 10 | -81 ± 16 | VU | EN | CR |
| *Lophopetalum duperreanum* | 103,441 | 30 | -12 | 26 | 42 | -11 | 69 | 15 ± 23 | 33 ± 41 | LC | LC | LC |
| *Lophopetalum wightianum* | 244,141 | -23 | -68 | 18 | -16 | -60 | -66 | -24 ± 43 | -47 ± 27 | LC | NT | VU |
| *Lyonia ovalifolia* | 219,871 | -35 | -63 | -42 | -79 | -72 | -48 | -47 ± 14 | -66 ± 16 | LC | VU | EN |
| *Maclura cochinchinensis* | 194,327 | -26 | -49 | -4 | -55 | -61 | -61 | -26 ± 23 | -59 ± 3 | LC | NT | EN |
| *Maclura fruticosa* | 98,561 | -45 | -82 | -12 | -70 | -98 | -42 | -46 ± 35 | -70 ± 28 | LC | VU | EN |
| *Maerua siamensis* | 70,243 | 50 | 60 | 80 | 86 | 93 | 99 | 63 ± 15 | 93 ± 7 | LC | LC | LC |
| *Magnolia baillonii* | 41,215 | -39 | -68 | -79 | -76 | -77 | -87 | -62 ± 21 | -80 ± 6 | LC | EN | EN |
| *Magnolia elegans* | 21,211 | -8 | -46 | 111 | -10 | -83 | 55 | 19 ± 82 | -13 ± 69 | NT | LC | NT |
| *Magnolia rajaniana* | 39,468 | -56 | -85 | -24 | -85 | -92 | -82 | -55 ± 31 | -86 ± 5 | LC | EN | CR |

| | | | | | | | | | | | | |
|---|---|---|---|---|---|---|---|---|---|---|---|---|
| *Mallotus macrostachyus* | 10,509 | -53 | -77 | 3 | -52 | -96 | -85 | -42 ± 41 | -78 ± 23 | VU | VU | EN |
| *Mallotus philippensis* | 92,158 | 0 | 24 | -23 | -5 | -2 | -20 | 0 ± 23 | -9 ± 10 | LC | LC | NT |
| *Mammea siamensis* | 115,276 | 30 | 126 | 131 | 100 | 76 | 127 | 95 ± 57 | 101 ± 25 | LC | LC | LC |
| *Mangifera caloneura* | 113,828 | 30 | -19 | 3 | 4 | -23 | -51 | 5 ± 25 | -23 ± 28 | LC | LC | NT |
| *Mangifera cochinchinensis* | 64,598 | 4 | -53 | 27 | -24 | -1 | 121 | -8 ± 41 | 32 ± 78 | LC | NT | LC |
| *Mansonia gagei* | 149,863 | -28 | -65 | -20 | -57 | -83 | 52 | -37 ± 24 | -30 ± 72 | LC | VU | VU |
| *Melia azedarach* | 395,852 | -15 | -33 | -8 | -33 | -56 | -1 | -18 ± 13 | -30 ± 27 | LC | NT | VU |
| *Melientha suavis* | 114,885 | 19 | 19 | 83 | 21 | 56 | 122 | 41 ± 37 | 67 ± 51 | LC | LC | LC |
| *Melodorum fruticosum* | 61,643 | 22 | -50 | 76 | 40 | 38 | 68 | 16 ± 63 | 49 ± 17 | LC | LC | LC |
| *Memecylon ovatum* | 44,880 | -29 | -79 | -59 | -49 | -85 | -83 | -55 ± 25 | -72 ± 20 | LC | EN | EN |
| *Mesua ferrea* | 80,697 | -38 | -59 | -20 | -79 | -93 | -86 | -39 ± 20 | -86 ± 7 | LC | VU | CR |
| *Microcos tomentosa* | 91,633 | 35 | -41 | -20 | 1 | -9 | -74 | -9 ± 39 | -27 ± 41 | LC | NT | NT |
| *Millettia brandisiana* | 325,303 | -9 | -22 | 2 | -4 | -29 | 26 | -10 ± 12 | -2 ± 28 | LC | NT | NT |
| *Millettia leucantha* | 121,688 | 43 | 14 | 31 | 56 | 10 | -15 | 29 ± 15 | 17 ± 36 | LC | LC | LC |
| *Millettia pachycarpa* | 166,553 | -20 | 7 | 27 | 0 | -68 | 22 | 5 ± 24 | -15 ± 47 | LC | LC | NT |
| *Millettia xylocarpa* | 344,217 | 0 | 18 | 43 | 28 | 15 | 42 | 20 ± 21 | 29 ± 13 | LC | LC | LC |
| *Mischocarpus pentapetalus* | 107,617 | -45 | -49 | 79 | -49 | -41 | 84 | -5 ± 73 | -2 ± 75 | LC | NT | NT |
| *Mischocarpus sundaicus* | 41,827 | 101 | 245 | 192 | 791 | 518 | 460 | 179 ± 73 | 590 ± 177 | LC | LC | LC |
| *Mitrephora vandaeflora* | 268,728 | -49 | -89 | -72 | -93 | -100 | -98 | -70 ± 20 | -97 ± 3 | LC | EN | CR |
| *Mitrephora winitii* | 2,764 | -10 | -64 | -89 | -94 | -94 | -100 | -54 ± 40 | -96 ± 4 | EN | EN | CR |
| *Monoon lateriflorum* | 14,340 | -8 | 19 | -13 | 1 | -40 | -60 | -1 ± 17 | -33 ± 31 | VU | NT | VU |
| *Monoon membranifolium* | 3,714 | -45 | -75 | -5 | -58 | -96 | -82 | -42 ± 35 | -79 ± 20 | EN | VU | EN |
| *Morus macroura* | 64,638 | -55 | -56 | -30 | -72 | -95 | -63 | -47 ± 15 | -77 ± 16 | LC | VU | EN |
| *Mucuna bracteata* | 132,974 | -45 | -48 | 18 | -60 | -82 | -65 | -25 ± 37 | -69 ± 11 | LC | NT | EN |
| *Mucuna macrocarpa* | 194,069 | -35 | -71 | -29 | -66 | -92 | -64 | -45 ± 22 | -74 ± 16 | LC | VU | EN |
| *Mucuna pruriens* | 321,030 | -3 | -24 | 14 | 3 | -36 | 27 | -4 ± 19 | -2 ± 32 | LC | NT | NT |
| *Mucuna revoluta* | 182,851 | 11 | 33 | 28 | 5 | 21 | 122 | 24 ± 12 | 49 ± 64 | LC | LC | LC |
| *Munronia humilis* | 40,391 | -47 | -57 | 90 | -81 | -83 | -71 | -5 ± 82 | -78 ± 6 | LC | NT | EN |
| *Murraya paniculata* | 92,571 | 32 | -32 | 30 | 32 | 0 | -32 | 10 ± 37 | 0 ± 32 | LC | LC | LC |
| *Myrica esculenta* | 73,923 | -39 | -38 | 1 | -28 | -89 | -25 | -25 ± 23 | -47 ± 36 | LC | NT | VU |
| *Nageia wallichiana* | 50,172 | -71 | -90 | -79 | -89 | -98 | -98 | -80 ± 10 | -95 ± 5 | LC | EN | CR |
| *Naringi crenulata* | 98,373 | -25 | -78 | -78 | -75 | -100 | -99 | -60 ± 31 | -91 ± 14 | LC | EN | CR |
| *Nauclea orientalis* | 54,049 | -10 | -69 | -12 | -53 | -83 | -71 | -30 ± 33 | -69 ± 16 | LC | VU | EN |
| *Neolamarckia cadamba* | 133,673 | 28 | 61 | 51 | 41 | 5 | 17 | 47 ± 17 | 21 ± 19 | LC | LC | LC |
| *Neolitsea zeylanica* | 120,699 | -31 | -23 | 53 | -3 | -43 | 7 | -1 ± 46 | -13 ± 27 | LC | NT | NT |
| *Nephelium cuspidatum* | 13,844 | -34 | -26 | 3 | -52 | -77 | -70 | -19 ± 20 | -66 ± 13 | VU | NT | EN |
| *Nephelium hypoleucum* | 85,502 | 22 | -25 | 13 | -1 | -24 | -47 | 3 ± 25 | -24 ± 23 | LC | LC | NT |
| *Nephelium melliferum* | 4,336 | -39 | -45 | -41 | -61 | -97 | -89 | -42 ± 3 | -83 ± 19 | EN | VU | CR |
| *Nothaphoebe umbelliflora* | 62,571 | -35 | 102 | 83 | -7 | -78 | 11 | 50 ± 74 | -25 ± 47 | LC | LC | NT |

| | | | | | | | | | | | | |
|---|---|---|---|---|---|---|---|---|---|---|---|---|
| *Ochna integerrima* | 142,670 | 43 | 71 | 65 | 41 | 52 | 68 | 60 ± 15 | 54 ± 14 | LC | LC | LC |
| *Ostodes paniculata* | 61,945 | -27 | -27 | 45 | -43 | -67 | -50 | -3 ± 41 | -53 ± 13 | LC | NT | EN |
| *Pachyrhizus erosus* | 350,689 | -20 | -35 | -14 | -46 | -83 | -55 | -23 ± 11 | -61 ± 20 | LC | NT | EN |
| *Paranephelium spirei* | 51,429 | 17 | -2 | -35 | -78 | -84 | -4 | -7 ± 26 | -55 ± 45 | LC | NT | EN |
| *Parashorea densiflora* | 1,227 | 1243 | 131 | -100 | 196 | -68 | -100 | 425 ± 718 | 9 ± 162 | EN | LC | LC |
| *Parashorea stellata* | 11,962 | -51 | -92 | -45 | -86 | -99 | -89 | -63 ± 25 | -92 ± 7 | VU | EN | CR |
| *Parinari anamense* | 154,524 | 71 | 109 | 62 | 116 | 13 | 96 | 80 ± 25 | 75 ± 54 | LC | LC | LC |
| *Parinari anamensis* | 133,510 | 1 | -61 | -56 | -60 | -49 | -81 | -39 ± 34 | -63 ± 17 | LC | VU | EN |
| *Parkia speciosa* | 24,307 | -43 | -59 | -40 | -77 | -78 | -83 | -47 ± 10 | -79 ± 3 | NT | VU | EN |
| *Parkia sumatrana* | 44,423 | 47 | -61 | -79 | -66 | 27 | -83 | -31 ± 68 | -41 ± 60 | LC | VU | VU |
| *Persea kurzii* | 86,687 | -31 | -86 | -77 | -87 | -84 | -95 | -65 ± 30 | -89 ± 6 | LC | EN | CR |
| *Phylacium majus* | 225,473 | -26 | -48 | 15 | -37 | -65 | -51 | -20 ± 32 | -51 ± 14 | LC | NT | EN |
| *Phyllanthus angkorensis* | 85,795 | -12 | -56 | 10 | -67 | -20 | -55 | -20 ± 33 | -48 ± 24 | LC | NT | VU |
| *Phyllanthus emblica* | 120,610 | -11 | 32 | 31 | 18 | 42 | -17 | 18 ± 25 | 14 ± 30 | LC | LC | LC |
| *Phyllodium kurzianum* | 203,292 | -20 | -27 | 3 | -36 | -61 | 75 | -15 ± 16 | -7 ± 72 | LC | NT | NT |
| *Phyllodium pulchellum* | 383,991 | -4 | -24 | 12 | -3 | -19 | -5 | -5 ± 18 | -9 ± 9 | LC | NT | NT |
| *Pinus kesiya* | 45,088 | -33 | -71 | 16 | -47 | -52 | -88 | -29 ± 44 | -62 ± 22 | LC | NT | EN |
| *Pinus merkusii* | 46,553 | -18 | 3 | -2 | 41 | 29 | 263 | -6 ± 11 | 111 ± 132 | LC | NT | LC |
| *Platymitra macrocarpa* | 18,881 | -44 | -64 | -32 | -52 | -78 | 158 | -47 ± 16 | 9 ± 129 | VU | VU | LC |
| *Pluchea polygonata* | 202,979 | -14 | -39 | 21 | -61 | -64 | 51 | -11 ± 30 | -25 ± 66 | LC | NT | NT |
| *Podocarpus neriifolius* | 30,368 | -37 | -67 | -38 | -74 | -88 | -81 | -47 ± 17 | -81 ± 7 | LC | VU | CR |
| *Pometia pinnata* | 56,796 | -36 | -60 | -48 | -74 | -74 | -83 | -48 ± 12 | -77 ± 5 | LC | VU | EN |
| *Protium serratum* | 82,120 | -9 | 6 | 26 | -11 | -64 | -40 | 8 ± 18 | -38 ± 27 | LC | LC | VU |
| *Pterocarpus macrocarpus* | 141,387 | 22 | 19 | 6 | 15 | 18 | -18 | 16 ± 9 | 5 ± 20 | LC | LC | LC |
| *Pterocymbium tinctorium* | 86,445 | 75 | 79 | 106 | 107 | 20 | 169 | 87 ± 17 | 99 ± 75 | LC | LC | LC |
| *Pueraria anabaptista* | 144,405 | -47 | -76 | 10 | -88 | -94 | -82 | -38 ± 44 | -88 ± 6 | LC | VU | CR |
| *Pueraria phaseoloides* | 521,818 | -2 | -1 | 11 | -1 | -4 | -8 | 3 ± 7 | -4 ± 3 | LC | LC | NT |
| *Pueraria stricta* | 156,558 | -41 | -78 | -22 | -73 | -99 | -30 | -47 ± 29 | -67 ± 35 | LC | VU | EN |
| *Pueraria wallichii* | 174,788 | -15 | -60 | 8 | -19 | -29 | 56 | -22 ± 35 | 3 ± 47 | LC | NT | LC |
| *Pyrenaria diospyricarpa* | 40,587 | -24 | -33 | 65 | -54 | -81 | -43 | 3 ± 54 | -59 ± 19 | LC | LC | EN |
| *Quercus brandisiana* | 18,821 | -65 | -34 | -17 | -58 | -99 | -100 | -39 ± 24 | -86 ± 24 | VU | VU | CR |
| *Quercus kerrii* | 101,996 | 17 | 34 | 13 | -16 | 33 | -29 | 22 ± 11 | -4 ± 32 | LC | LC | NT |
| *Quercus kingiana* | 150,246 | -11 | -50 | 35 | -24 | -61 | 70 | -9 ± 43 | -5 ± 68 | LC | NT | NT |
| *Quercus oidocarpa* | 74,070 | -23 | -45 | 10 | -92 | -92 | 6 | -19 ± 27 | -59 ± 56 | LC | NT | EN |
| *Quercus rex* | 297,121 | -50 | -91 | -45 | -94 | -88 | -70 | -62 ± 25 | -84 ± 12 | LC | EN | CR |
| *Radermachera ignea* | 121,693 | -54 | -75 | -35 | -86 | -99 | -85 | -55 ± 20 | -90 ± 8 | LC | EN | CR |
| *Rhododendron ludwigianum* | 86,320 | -60 | -78 | -7 | -63 | -98 | -57 | -49 ± 37 | -73 ± 22 | LC | VU | EN |
| *Rinorea anguifera* | 3,125 | -1 | 4 | 133 | -16 | 112 | 339 | 45 ± 76 | 145 ± 180 | EN | LC | LC |
| *Rothmannia sootepensis* | 118,415 | -43 | -79 | -40 | -64 | -90 | -84 | -54 ± 22 | -79 ± 14 | LC | EN | EN |

| | | | | | | | | | | | | |
|---|---|---|---|---|---|---|---|---|---|---|---|---|
| *Rothmannia wittii* | 17,749 | 37 | -82 | -87 | 32 | -56 | -83 | -44 ± 70 | -35 ± 60 | VU | VU | VU |
| *Sageraea elliptica* | 57,775 | 3 | 2 | -15 | -2 | -42 | -17 | -3 ± 10 | -20 ± 20 | LC | NT | NT |
| *Sandoricum koetjape* | 20,679 | -8 | 12 | -13 | 33 | 24 | 7 | -3 ± 13 | 21 ± 13 | NT | NT | LC |
| *Santiria laevigata* | 296 | -84 | -44 | 8034 | 396 | 212 | 9671 | 2635 ± 4676 | 3426 ± 5409 | EN | LC | LC |
| *Santisukia kerrii* | 202,181 | 1 | -11 | 14 | 2 | -36 | 52 | 1 ± 13 | 6 ± 44 | LC | LC | LC |
| *Santisukia pagetii* | 36,508 | -69 | -93 | -18 | -84 | -100 | -54 | -60 ± 39 | -79 ± 23 | LC | EN | EN |
| *Sapindus rarak* | 111,893 | -50 | -64 | -68 | -75 | -95 | -92 | -61 ± 10 | -87 ± 11 | LC | EN | CR |
| *Saraca declinata* | 119,487 | 51 | 18 | 61 | 57 | 149 | 176 | 44 ± 23 | 128 ± 62 | LC | LC | LC |
| *Saraca thaipingensis* | 389,316 | 26 | 34 | 33 | 37 | 40 | 39 | 31 ± 4 | 39 ± 1 | LC | LC | LC |
| *Sarcosperma arboreum* | 36,876 | -17 | -59 | -31 | -58 | -54 | -76 | -36 ± 21 | -63 ± 12 | LC | VU | EN |
| *Scaphium linearicarpum* | 8,511 | -53 | -89 | -23 | -84 | -99 | -91 | -55 ± 33 | -92 ± 8 | VU | EN | CR |
| *Scaphochlamys obcordata* | 8,487 | -25 | -99 | -94 | -99 | -100 | -100 | -73 ± 41 | -100 ± 0 | VU | EN | EX |
| *Schima wallichii* | 82,179 | -32 | -58 | -46 | -60 | -83 | -90 | -45 ± 13 | -78 ± 16 | LC | VU | EN |
| *Schleichera oleosa* | 105,619 | -36 | -31 | -36 | -80 | -79 | -79 | -34 ± 3 | -79 ± 0 | LC | VU | EN |
| *Schrebera swietenioides* | 140,592 | 0 | -4 | -21 | -13 | -5 | -10 | -8 ± 11 | -10 ± 4 | LC | NT | NT |
| *Semecarpus cochinchinensis* | 77,286 | 17 | 17 | 44 | 21 | 11 | -14 | 26 ± 15 | 6 ± 18 | LC | LC | LC |
| *Shorea farinosa* | 73,813 | -73 | -90 | -80 | -97 | -99 | -98 | -81 ± 9 | -98 ± 1 | LC | CR | CR |
| *Shorea gratissima* | 28,171 | -53 | -73 | -60 | -87 | -96 | -90 | -62 ± 10 | -91 ± 5 | NT | EN | CR |
| *Shorea guiso* | 58,892 | -51 | -85 | 17 | -79 | -95 | -14 | -40 ± 52 | -62 ± 43 | LC | VU | EN |
| *Shorea henryana* | 20,350 | 19 | -22 | 24 | 3 | -28 | 211 | 7 ± 25 | 62 ± 130 | NT | LC | LC |
| *Shorea hypochra* | 28,827 | -36 | -37 | -20 | -77 | -96 | -94 | -31 ± 9 | -89 ± 10 | NT | VU | CR |
| *Shorea obtusa* | 144,004 | -1 | 14 | -23 | -7 | 12 | -27 | -3 ± 19 | -8 ± 20 | LC | NT | NT |
| *Shorea roxburghii* | 122,456 | -29 | -18 | 6 | -39 | -81 | -70 | -13 ± 18 | -63 ± 22 | LC | NT | EN |
| *Shorea siamensis* | 120,376 | 21 | 57 | 30 | 39 | 96 | 28 | 36 ± 18 | 54 ± 36 | LC | LC | LC |
| *Shuteria vestita* | 294,271 | -31 | -75 | -14 | -69 | -89 | -50 | -40 ± 31 | -69 ± 19 | LC | VU | EN |
| *Sindora siamensis* | 93,168 | 40 | -3 | -54 | 4 | -11 | -32 | -6 ± 47 | -13 ± 18 | LC | NT | NT |
| *Siphonodon celastrineus* | 118,887 | 31 | 39 | 22 | 37 | 37 | 38 | 31 ± 9 | 37 ± 0 | LC | LC | LC |
| *Sisyrolepis muricata* | 190,490 | 3 | 43 | 86 | 34 | 38 | 21 | 44 ± 42 | 31 ± 9 | LC | LC | LC |
| *Smithia ciliata* | 49,876 | -11 | -53 | -37 | -31 | -57 | -13 | -33 ± 21 | -34 ± 23 | LC | VU | VU |
| *Spatholobus parviflorus* | 164,034 | -32 | -11 | -17 | -43 | -88 | -65 | -20 ± 11 | -65 ± 22 | LC | NT | EN |
| *Spondias lakonensis* | 113,941 | -52 | -80 | -39 | -66 | -98 | -61 | -57 ± 21 | -75 ± 20 | LC | EN | EN |
| *Spondias pinnata* | 145,980 | 9 | 15 | 1 | 2 | -19 | -44 | 8 ± 7 | -20 ± 23 | LC | LC | NT |
| *Sterculia foetida* | 113,530 | -3 | 38 | -18 | 1 | -80 | -78 | 6 ± 29 | -53 ± 46 | LC | LC | EN |
| *Sterculia pexa* | 107,944 | -15 | -25 | -35 | -45 | -64 | -85 | -25 ± 10 | -65 ± 20 | LC | NT | EN |
| *Stereospermum colias* | 239,805 | 9 | -5 | -5 | -2 | -41 | 12 | 0 ± 8 | -10 ± 27 | LC | LC | NT |
| *Stereospermum fimbriatum* | 83,018 | -24 | -1 | -17 | 23 | -59 | 7 | -14 ± 12 | -9 ± 43 | LC | NT | NT |
| *Streblus asper* | 346,327 | -10 | -30 | -12 | -7 | -21 | 17 | -17 ± 11 | -4 ± 20 | LC | NT | NT |
| *Streblus ilicifolius* | 178,107 | -6 | -7 | 122 | 93 | 7 | 16 | 36 ± 74 | 38 ± 47 | LC | LC | LC |

| | | | | | | | | | | | | |
|---|---|---|---|---|---|---|---|---|---|---|---|---|
| *Streblus taxoides* | 475,871 | 15 | 15 | 23 | 24 | 26 | 28 | 18 ± 4 | 26 ± 2 | LC | LC | LC |
| *Strychnos nux-vomica* | 132,708 | 69 | 188 | 105 | 96 | 105 | 51 | 121 ± 61 | 84 ± 29 | LC | LC | LC |
| *Stylosanthes sundaica* | 378,048 | -20 | -38 | -2 | -29 | -26 | -32 | -20 ± 18 | -29 ± 3 | LC | NT | NT |
| *Styrax benzoides* | 26,569 | -45 | -2 | 27 | -52 | -56 | -27 | -7 ± 37 | -45 ± 16 | NT | NT | VU |
| *Suregada multiflorum* | 123,619 | 10 | 39 | 1 | 3 | -4 | 11 | 17 ± 20 | 4 ± 8 | LC | LC | LC |
| *Symplocos cochinchinensis* | 48,454 | 87 | -16 | -39 | 83 | 25 | 128 | 10 ± 67 | 79 ± 52 | LC | LC | LC |
| *Syzygium cinereum* | 51,570 | 52 | 59 | 96 | 115 | -37 | 271 | 69 ± 24 | 116 ± 154 | LC | LC | LC |
| *Syzygium claviflorum* | 65,377 | -12 | 33 | -30 | 6 | 13 | -2 | -3 ± 33 | 6 ± 7 | LC | NT | LC |
| *Syzygium cumini* | 131,774 | 24 | 0 | 16 | 28 | 15 | -24 | 13 ± 12 | 6 ± 27 | LC | LC | LC |
| *Syzygium siamense* | 23,494 | -5 | -56 | -69 | 29 | -62 | -86 | -43 ± 34 | -40 ± 61 | NT | VU | VU |
| *Tadehagi triquetrum* | 304,464 | -21 | -40 | -10 | -45 | -61 | -25 | -24 ± 15 | -44 ± 18 | LC | NT | VU |
| *Tarennoidea wallichii* | 32,673 | -65 | -37 | -69 | -92 | -99 | -95 | -57 ± 17 | -95 ± 3 | LC | EN | CR |
| *Tectona grandis* | 66,349 | -1 | 84 | 57 | 5 | -25 | -15 | 47 ± 44 | -12 ± 16 | LC | LC | NT |
| *Terminalia alata* | 242,745 | -5 | -45 | 8 | -1 | -64 | 2 | -14 ± 28 | -21 ± 37 | LC | NT | NT |
| *Terminalia bellirica* | 122,427 | 14 | 27 | 23 | 21 | -18 | -42 | 21 ± 7 | -13 ± 32 | LC | LC | NT |
| *Terminalia chebula* | 121,693 | 28 | 67 | 34 | 48 | 53 | -16 | 43 ± 21 | 28 ± 38 | LC | LC | LC |
| *Terminalia citrina* | 88,730 | -50 | -68 | -68 | -88 | -98 | -92 | -62 ± 10 | -92 ± 5 | LC | EN | CR |
| *Terminalia myriocarpa* | 12,514 | -80 | -48 | 92 | -92 | -84 | -69 | -12 ± 91 | -82 ± 12 | VU | NT | CR |
| *Terminalia triptera* | 325,101 | 4 | -21 | 34 | 10 | -24 | 49 | 5 ± 28 | 12 ± 37 | LC | LC | LC |
| *Ternstroemia gymnanthera* | 38,897 | -56 | -11 | -21 | -64 | -92 | -62 | -30 ± 24 | -73 ± 17 | LC | VU | EN |
| *Tetrameles nudiflora* | 96,019 | 11 | -30 | -16 | -32 | -55 | -23 | -12 ± 21 | -36 ± 16 | LC | NT | VU |
| *Toona ciliata* | 76,644 | -34 | -35 | -30 | -76 | -90 | -91 | -33 ± 3 | -86 ± 8 | LC | VU | CR |
| *Trevesia palmata* | 92,491 | -38 | -68 | -56 | -78 | -87 | -90 | -54 ± 15 | -85 ± 6 | LC | EN | CR |
| *Trigonobalanus doichangensis* | 107,555 | 3 | -29 | 46 | 16 | 3 | 144 | 7 ± 38 | 54 ± 78 | LC | LC | LC |
| *Ulmus lanceifolia* | 44,177 | -55 | -74 | -5 | -64 | -99 | -73 | -44 ± 36 | -78 ± 18 | LC | VU | EN |
| *Uraria campanulata* | 274,848 | -21 | -68 | -16 | -42 | -91 | -39 | -35 ± 29 | -57 ± 29 | LC | VU | EN |
| *Uraria cordifolia* | 145,231 | -24 | -63 | 42 | -53 | -54 | 71 | -15 ± 53 | -12 ± 72 | LC | NT | NT |
| *Uraria crinita* | 325,985 | -2 | -13 | 42 | 8 | -16 | 24 | 9 ± 29 | 6 ± 20 | LC | LC | LC |
| *Uraria lagopodioides* | 269,632 | -7 | 15 | 17 | 11 | -52 | 9 | 9 ± 13 | -11 ± 36 | LC | LC | NT |
| *Vaccinium sprengelii* | 190,493 | -6 | -60 | 22 | -42 | -57 | 4 | -14 ± 42 | -32 ± 32 | LC | NT | VU |
| *Vaccinium viscifolium* | 91,154 | -49 | -83 | -60 | -92 | -100 | -91 | -64 ± 18 | -94 ± 5 | LC | EN | CR |
| *Vatica diospyroides* | 65,068 | 99 | 8 | 44 | -5 | 34 | -35 | 50 ± 46 | -2 ± 35 | LC | LC | NT |
| *Vatica harmandiana* | 128,890 | -2 | -68 | -28 | -68 | -75 | -53 | -33 ± 33 | -65 ± 11 | LC | VU | EN |
| *Vatica mangachapoi* | 2,084 | 65 | -97 | -41 | -100 | -100 | -93 | -24 ± 82 | -98 ± 4 | EN | NT | CR |
| *Vatica odorata* | 46,352 | -37 | -73 | -52 | -73 | -91 | -87 | -54 ± 18 | -84 ± 10 | LC | EN | CR |
| *Vatica philastreana* | 177,967 | 18 | 29 | 24 | -5 | 37 | 16 | 24 ± 6 | 16 ± 21 | LC | LC | LC |
| *Vatica stapfiana* | 1,468 | -100 | 291 | 1122 | 597 | -89 | 1627 | 438 ± 624 | 712 ± 863 | EN | LC | LC |
| *Vernonia divergens* | 125,000 | -14 | -30 | 88 | 19 | -49 | 14 | 15 ± 64 | -6 ± 38 | LC | LC | NT |
| *Vernonia parishii* | 240,382 | -31 | -47 | 0 | -77 | -80 | -51 | -26 ± 24 | -69 ± 16 | LC | NT | EN |

| | | | | | | | | | | | | |
|---|---|---|---|---|---|---|---|---|---|---|---|---|
| *Vernonia squarrosa* | 209,515 | -10 | 14 | 50 | 0 | 22 | 97 | 18 ± 30 | 40 ± 50 | LC | LC | LC |
| *Vernonia sutepensis* | 76,166 | -44 | -87 | 34 | -66 | -100 | -29 | -32 ± 62 | -65 ± 35 | LC | VU | EN |
| *Vigna exilis* | 95,067 | -87 | -92 | -98 | -94 | -100 | -100 | -92 ± 6 | -98 ± 3 | LC | CR | CR |
| *Vitex peduncularis* | 137,062 | 12 | 15 | -3 | -5 | -14 | -50 | 8 ± 10 | -23 ± 24 | LC | LC | NT |
| *Vitex pinnata* | 143,020 | 14 | 19 | 9 | 10 | -13 | -48 | 14 ± 5 | -17 ± 29 | LC | LC | NT |
| *Wrightia arborea* | 121,091 | 29 | 27 | 12 | 15 | -26 | -44 | 23 ± 10 | -18 ± 31 | LC | LC | NT |
| *Wrightia pubescens* | 62,773 | 91 | 177 | 139 | 137 | 193 | 125 | 136 ± 43 | 151 ± 36 | LC | LC | LC |
| *Xanthophyllum flavescens* | 50,056 | -26 | -44 | -15 | -58 | -73 | -52 | -29 ± 15 | -61 ± 11 | LC | NT | EN |
| *Xantolis cambodiana* | 244,005 | -3 | -36 | 7 | -25 | -45 | 5 | -10 ± 22 | -22 ± 25 | LC | NT | NT |
| *Xerospermum noronhianum* | 162,152 | -20 | -67 | -45 | -69 | -70 | -89 | -44 ± 23 | -76 ± 11 | LC | VU | EN |
| *Xylia xylocarpa* | 127,951 | 14 | -3 | -7 | -14 | -57 | -77 | 1 ± 11 | -49 ± 33 | LC | LC | VU |
| *Xylocarpus granatum* | 504,690 | 13 | 10 | 6 | 20 | 20 | -20 | 10 ± 3 | 6 ± 23 | LC | LC | LC |
| *Zanthoxylum rhetsa* | 84,581 | 34 | 55 | 30 | 138 | 118 | 42 | 40 ± 14 | 99 ± 51 | LC | LC | LC |
| *Zingiber bradleyanum* | 152,497 | -28 | -51 | 17 | -31 | -70 | -48 | -21 ± 34 | -49 ± 20 | LC | NT | VU |
| *Zingiber kerrii* | 175,423 | -14 | -34 | 7 | -20 | -74 | 9 | -13 ± 20 | -28 ± 42 | LC | NT | NT |
| *Zingiber smilesianum* | 247,045 | -25 | -58 | -11 | -45 | -92 | -57 | -31 ± 24 | -65 ± 24 | LC | VU | EN |
| *Zollingeria dongnaiensis* | 78,748 | 70 | 153 | 17 | 219 | 236 | 135 | 80 ± 68 | 196 ± 54 | LC | LC | LC |
| *Zornia gibbosa* | 410,314 | -9 | -25 | -6 | -9 | -34 | 13 | -13 ± 10 | -10 ± 23 | LC | NT | NT |

**Table S8** Predicted current suitable habitat for each reptile species, projected change by 2070, and estimated conservation status using three earth system models and two RCPs.

| species | suitable habitat at present ($km^2$) | % change in suitable habitat under RCP2.6 | | | % change in suitable habitat under RCP8.5 | | | mean of suitable habitat change | | Conservation Status | | |
|---|---|---|---|---|---|---|---|---|---|---|---|---|
| | | CNRM-CM5 | GFDL-CM3 | HadGEM2-ES | CNRM-CM5 | GFDL-CM3 | HadGEM2-ES | RCP 2.6 | RCP 8.5 | Current | RCP 2.6 | RCP 8.5 |
| *Acanthosaura lepidogaster* | 19,985 | -41 | -99 | -92 | -88 | -97 | -95 | -77 ± 32 | -93 ± 5 | VU | EN | CR |
| *Ahaetulla prasina* | 193,559 | -45 | -93 | -77 | -95 | -97 | -98 | -72 ± 24 | -97 ± 1 | LC | EN | CR |
| *Boiga cyanea* | 386,789 | 16 | -7 | 27 | 10 | 7 | 42 | 12 ± 17 | 19 ± 19 | LC | LC | LC |
| *Calloselasma rhodostoma* | 274,597 | -2 | -4 | 21 | 35 | -4 | 31 | 5 ± 14 | 20 ± 21 | LC | LC | LC |
| *Calotes emma* | 57,896 | -40 | -75 | -58 | -83 | -87 | -94 | -58 ± 17 | -88 ± 5 | LC | EN | CR |
| *Calotes mystaceus* | 143,887 | 5 | 22 | 14 | -41 | -59 | -64 | 14 ± 8 | -55 ± 12 | LC | LC | EN |
| *Calotes versicolor* | 264,767 | -20 | -62 | -36 | -45 | -62 | -89 | -39 ± 21 | -65 ± 22 | LC | VU | EN |
| *Chelonia mydas* | 988 | 131 | 833 | -76 | 226 | -51 | 119 | 296 ± 477 | 98 ± 140 | EN | LC | LC |
| *Chrysopelea ornata* | 223,786 | 41 | 76 | 69 | 94 | 147 | 106 | 62 ± 19 | 116 ± 28 | LC | LC | LC |
| *Crocodylus siamensis* | 201,090 | -4 | -26 | 18 | -18 | -43 | 72 | -4 ± 22 | 3 ± 61 | LC | NT | LC |
| *Cuora amboinensis* | 395,318 | 27 | 23 | -84 | 31 | 52 | 53 | -11 ± 63 | 46 ± 12 | LC | NT | LC |
| *Cyrtodactylus intermedius* | 81,560 | -36 | -86 | -14 | -54 | -92 | 4 | -45 ± 37 | -48 ± 49 | LC | VU | VU |
| *Dasia olivacea* | 89,173 | 129 | 238 | -32 | 298 | 231 | 404 | 111 ± 136 | 311 ± 87 | LC | LC | LC |
| *Dendrelaphis pictus* | 363,594 | 35 | 45 | 35 | 60 | 48 | 47 | 39 ± 6 | 52 ± 7 | LC | LC | LC |
| *Dixonius siamensis* | 265,125 | 11 | -2 | 59 | 39 | 29 | 63 | 23 ± 32 | 44 ± 17 | LC | LC | LC |
| *Draco maculatus* | 240,425 | -40 | -76 | -42 | -74 | -93 | -62 | -53 ± 20 | -76 ± 16 | LC | EN | EN |
| *Draco sumatranus* | 30,794 | 81 | 139 | 20 | 122 | 74 | 354 | 80 ± 59 | 183 ± 149 | LC | LC | LC |
| *Draco taeniopterus* | 161,708 | 34 | 9 | 56 | 95 | 96 | 144 | 33 ± 23 | 112 ± 28 | LC | LC | LC |
| *Enhydris enhydris* | 159,306 | 152 | 96 | 214 | 224 | 132 | 187 | 154 ± 59 | 181 ± 46 | LC | LC | LC |
| *Eutropis longicaudata* | 400,702 | 19 | 38 | 40 | 39 | 41 | 48 | 32 ± 11 | 43 ± 4 | LC | LC | LC |
| *Eutropis macularia* | 220,567 | -18 | -18 | -53 | -48 | -68 | -71 | -30 ± 20 | -63 ± 12 | LC | VU | EN |
| *Eutropis multifasciata* | 219,099 | 41 | 2 | 97 | 49 | 30 | 83 | 47 ± 48 | 54 ± 27 | LC | LC | LC |
| *Gehyra mutilata* | 493,624 | 11 | 21 | 19 | 15 | 21 | 23 | 17 ± 5 | 20 ± 4 | LC | LC | LC |
| *Gekko gecko* | 240,861 | -3 | -65 | -8 | -43 | -69 | -55 | -26 ± 35 | -56 ± 13 | LC | NT | EN |
| *Gonyosoma oxycephalum* | 230,764 | 26 | 50 | 94 | 93 | 74 | 84 | 57 ± 34 | 84 ± 10 | LC | LC | LC |
| *Hemidactylus frenatus* | 138,025 | 43 | -9 | 22 | -15 | 125 | 148 | 19 ± 26 | 86 ± 88 | LC | LC | LC |

| | | | | | | | | | | | | |
|---|---|---|---|---|---|---|---|---|---|---|---|---|
| *Hemidactylus platyurus* | 179,167 | 0 | -47 | 14 | 10 | -13 | -85 | -11 ± 31 | -29 ± 50 | LC | NT | NT |
| *Heosemys annandalii* | 77,959 | 83 | 245 | 341 | 161 | 428 | 449 | 223 ± 130 | 346 ± 161 | LC | LC | LC |
| *Hypsiscopus plumbea* | 216,101 | 29 | 77 | 78 | 56 | 93 | 83 | 61 ± 28 | 77 ± 19 | LC | LC | LC |
| *Indotestudo elongata* | 90,562 | -21 | -58 | -25 | -50 | -1 | -58 | -34 ± 20 | -36 ± 31 | LC | VU | VU |
| *Indotyphlops braminus* | 512,571 | -1 | 13 | 5 | 16 | -11 | 11 | 6 ± 7 | 5 ± 14 | LC | LC | LC |
| *Leiolepis belliana* | 43,991 | 202 | 224 | 439 | 400 | 89 | 724 | 288 ± 131 | 404 ± 318 | LC | LC | LC |
| *Lipinia vittigera* | 501,674 | -2 | -9 | 3 | -13 | -3 | 4 | -3 ± 6 | -4 ± 9 | LC | NT | NT |
| *Lycodon capucinus* | 296,766 | 19 | 22 | 38 | 5 | -8 | 44 | 26 ± 10 | 14 ± 27 | LC | LC | LC |
| *Lycodon laoensis* | 416,959 | 17 | 22 | 23 | 27 | 35 | 32 | 21 ± 3 | 31 ± 4 | LC | LC | LC |
| *Lygosoma bowringii* | 534,027 | 2 | 12 | -6 | 11 | -38 | -4 | 2 ± 9 | -10 ± 25 | LC | LC | NT |
| *Malayemys macrocephala* | 67,751 | 229 | 184 | 78 | 348 | 217 | 354 | 164 ± 78 | 306 ± 77 | LC | LC | LC |
| *Malayopython reticulatus* | 196,298 | -14 | 14 | 105 | -9 | 3 | 123 | 35 ± 62 | 39 ± 73 | LC | LC | LC |
| *Naja kaouthia* | 427,276 | 20 | 31 | 19 | 36 | 39 | 42 | 23 ± 7 | 39 ± 3 | LC | LC | LC |
| *Oligodon fasciolatus* | 370,056 | -10 | -16 | 16 | -3 | 12 | 34 | -3 ± 17 | 14 ± 19 | LC | NT | LC |
| *Physignathus cocincinus* | 256,759 | -18 | -61 | -30 | -59 | -64 | -3 | -36 ± 22 | -42 ± 34 | LC | VU | VU |
| *Ptyas korros* | 346,166 | 15 | 30 | 21 | 8 | 7 | 24 | 22 ± 8 | 13 ± 9 | LC | LC | LC |
| *Rhabdophis nigrocinctus* | 287,178 | 47 | 58 | 68 | 84 | 75 | 83 | 58 ± 11 | 81 ± 5 | LC | LC | LC |
| *Rhabdophis subminiatus* | 388,114 | 13 | 50 | 54 | 56 | 35 | 43 | 39 ± 23 | 45 ± 10 | LC | LC | LC |
| *Sphenomorphus maculatus* | 40,382 | -20 | -53 | -32 | -24 | -78 | -91 | -35 ± 17 | -64 ± 35 | LC | VU | EN |
| *Trimeresurus albolabris* | 226,159 | -16 | -20 | 27 | 1 | -16 | 82 | -3 ± 26 | 22 ± 53 | LC | NT | LC |
| *Trimeresurus macrops* | 397,124 | -7 | -64 | -63 | -28 | -54 | 15 | -45 ± 33 | -22 ± 35 | LC | VU | NT |
| *Trimeresurus vogeli* | 24,137 | -79 | -100 | -43 | -68 | -94 | -92 | -74 ± 29 | -85 ± 14 | NT | EN | CR |
| *Varanus bengalensis* | 56,005 | -3 | -26 | -18 | -20 | -78 | -73 | -16 ± 12 | -57 ± 32 | LC | NT | EN |
| *Varanus nebulosus* | 182,373 | -12 | -75 | 75 | 59 | 23 | 17 | -4 ± 75 | 33 ± 22 | LC | NT | LC |
| *Varanus salvator* | 90,261 | 25 | 94 | 52 | -31 | -31 | -57 | 57 ± 35 | -40 ± 15 | LC | LC | VU |
| *Xenochrophis flavipunctatus* | 290,257 | 30 | 55 | 52 | 58 | 29 | 65 | 46 ± 13 | 51 ± 19 | LC | LC | LC |
| *Xenochrophis piscator* | 212,178 | 52 | -15 | 56 | -32 | 46 | 27 | 31 ± 40 | 14 ± 41 | LC | LC | LC |

**Table S9** Predicted current suitable habitat for each amphibian species, projected change by 2070, and estimated conservation status using three earth system models and two RCPs.

| species | suitable habitat at present (km$^2$) | % change in suitable habitat under RCP2.6 | | | % change in suitable habitat under RCP8.5 | | | mean of suitable habitat change | | Conservation Status | | |
|---|---|---|---|---|---|---|---|---|---|---|---|---|
| | | CNRM-CM5 | GFDL-CM3 | HadGEM2-ES | CNRM-CM5 | GFDL-CM3 | HadGEM2-ES | RCP 2.6 | RCP 8.5 | Current | RCP 2.6 | RCP 8.5 |
| *Amolops panhai* | 75,005 | -2 | -78 | -72 | -47 | -69 | -95 | -51 ± 42 | -70 ± 24 | LC | EN | EN |
| *Calluella guttulata* | 275,504 | 21 | 34 | 18 | 42 | 3 | 36 | 24 ± 9 | 27 ± 21 | LC | LC | LC |
| *Duttaphrynus melanostictus* | 226,243 | 14 | 56 | 69 | 58 | 89 | 115 | 47 ± 28 | 87 ± 28 | LC | LC | LC |
| *Fejervarya cancrivora* | 298,164 | 42 | 84 | 85 | 94 | 98 | 101 | 70 ± 25 | 98 ± 4 | LC | LC | LC |
| *Fejervarya limnocharis* | 245,022 | 88 | 105 | 115 | 132 | 0 | 145 | 102 ± 14 | 93 ± 80 | LC | LC | LC |
| *Glyphoglossus molossus* | 134,447 | 132 | 241 | 190 | 269 | 132 | 314 | 188 ± 54 | 238 ± 95 | LC | LC | LC |
| *Hoplobatrachus rugulosus* | 352,887 | 21 | 41 | 34 | 49 | 40 | 61 | 32 ± 10 | 50 ± 11 | LC | LC | LC |
| *Hylarana erythraea* | 318,026 | 35 | 58 | 60 | 46 | 51 | 82 | 51 ± 14 | 59 ± 20 | LC | LC | LC |
| *Hylarana eschatia* | 25,637 | 41 | 102 | -22 | -51 | -80 | -29 | 40 ± 62 | -53 ± 26 | NT | LC | EN |
| *Hylarana macrodactyla* | 238,232 | 16 | 23 | 143 | 150 | 151 | 151 | 60 ± 71 | 150 ± 1 | LC | LC | LC |
| *Ichthyophis kohtaoensis* | 342,174 | 34 | 52 | 59 | 63 | 72 | 57 | 48 ± 13 | 64 ± 7 | LC | LC | LC |
| *Ingerophrynus parvus* | 242,587 | 42 | 60 | 65 | 61 | 80 | 83 | 55 ± 12 | 75 ± 12 | LC | LC | LC |
| *Kaloula pulchra* | 197,348 | 43 | 116 | 28 | 109 | 75 | 135 | 62 ± 47 | 106 ± 30 | LC | LC | LC |
| *Leptobrachium hendricksoni* | 150,346 | 0 | -9 | -98 | -98 | 2 | 28 | -36 ± 54 | -22 ± 67 | LC | VU | NT |
| *Limnonectes blythii* | 183,983 | 3 | 38 | 36 | -9 | -56 | -55 | 26 ± 20 | -40 ± 27 | LC | LC | VU |
| *Limnonectes gyldenstolpei* | 177,798 | -16 | -68 | -12 | -34 | -60 | 0 | -32 ± 31 | -31 ± 30 | LC | VU | VU |
| *Microhyla berdmorei* | 224,056 | -20 | -31 | -4 | -18 | -91 | 55 | -18 ± 14 | -18 ± 73 | LC | NT | NT |
| *Microhyla butleri* | 174,738 | 36 | 58 | 53 | 59 | 17 | 96 | 49 ± 12 | 57 ± 39 | LC | LC | LC |
| *Microhyla fissipes* | 471,024 | 19 | 24 | 26 | 19 | 10 | 21 | 23 ± 4 | 17 ± 6 | LC | LC | LC |
| *Microhyla heymonsi* | 242,548 | 38 | 99 | 105 | 125 | 118 | 118 | 81 ± 37 | 120 ± 4 | LC | LC | LC |
| *Microhyla ornata* | 385,312 | 32 | 38 | 40 | 49 | 31 | 37 | 36 ± 4 | 39 ± 9 | LC | LC | LC |
| *Microhyla pulchra* | 380,901 | -5 | 4 | -31 | -27 | -51 | 3 | -10 ± 18 | -25 ± 27 | LC | NT | NT |
| *Micryletta inornata* | 434,775 | 3 | -30 | 31 | 14 | -16 | 17 | 1 ± 31 | 5 ± 18 | LC | LC | LC |
| *Occidozyga lima* | 311,963 | 16 | -51 | -52 | -21 | -22 | -19 | -29 ± 39 | -21 ± 1 | LC | NT | NT |
| *Occidozyga martensii* | 271,128 | 58 | 90 | 74 | 69 | 83 | 97 | 74 ± 16 | 83 ± 14 | LC | LC | LC |
| *Odorrana hosii* | 27,908 | 88 | 29 | 22 | 266 | -62 | 36 | 46 ± 37 | 80 ± 168 | NT | LC | LC |
| *Phrynoidis aspera* | 113,584 | 53 | 17 | 54 | 190 | 69 | 124 | 41 ± 21 | 128 ± 61 | LC | LC | LC |

| | | | | | | | | | | | | |
|---|---|---|---|---|---|---|---|---|---|---|---|---|
| *Polypedates leucomystax* | 173,630 | -17 | -76 | -45 | -24 | -61 | -53 | -46 ± 29 | -46 ± 19 | LC | VU | VU |
| *Polypedates megacephalus* | 270,596 | -40 | -53 | -22 | -89 | -72 | -91 | -38 ± 15 | -84 ± 11 | LC | VU | CR |
| *Sylvirana mortenseni* | 514,560 | -5 | -67 | -16 | -39 | -48 | -52 | -29 ± 33 | -46 ± 7 | LC | NT | VU |
| *Sylvirana nigrovittata* | 296,469 | -11 | -33 | -2 | -46 | -97 | -83 | -16 ± 16 | -75 ± 26 | LC | NT | EN |

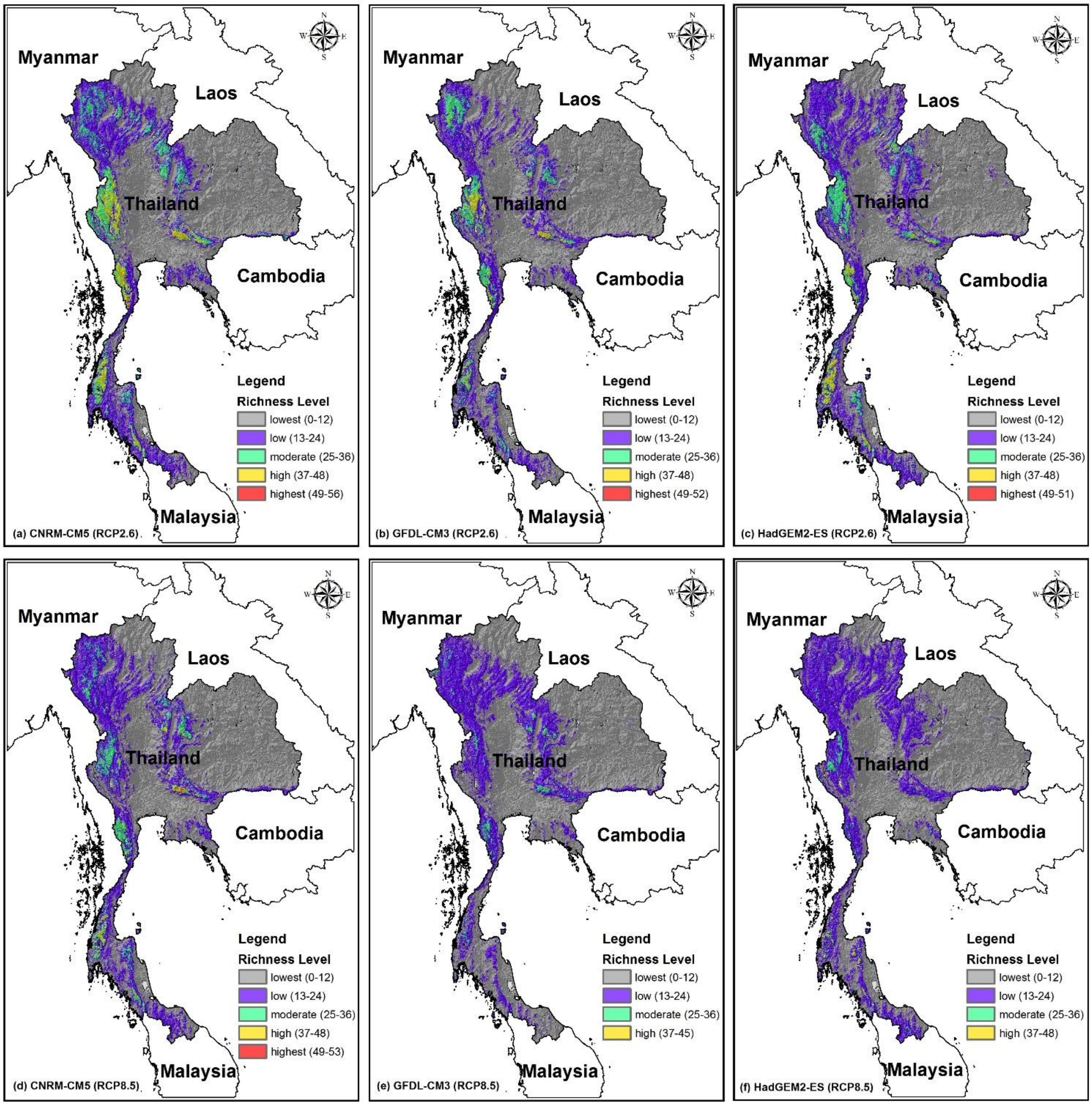


**Fig. S1** Spatial patterns of predicted species richness levels for mammals in 2070 with three earth system models and two RCPs. Maps created in ArcMap 10.5 (https://support.esri.com/en/products/desktop/arcgis-desktop/arcmap/10-5-1).

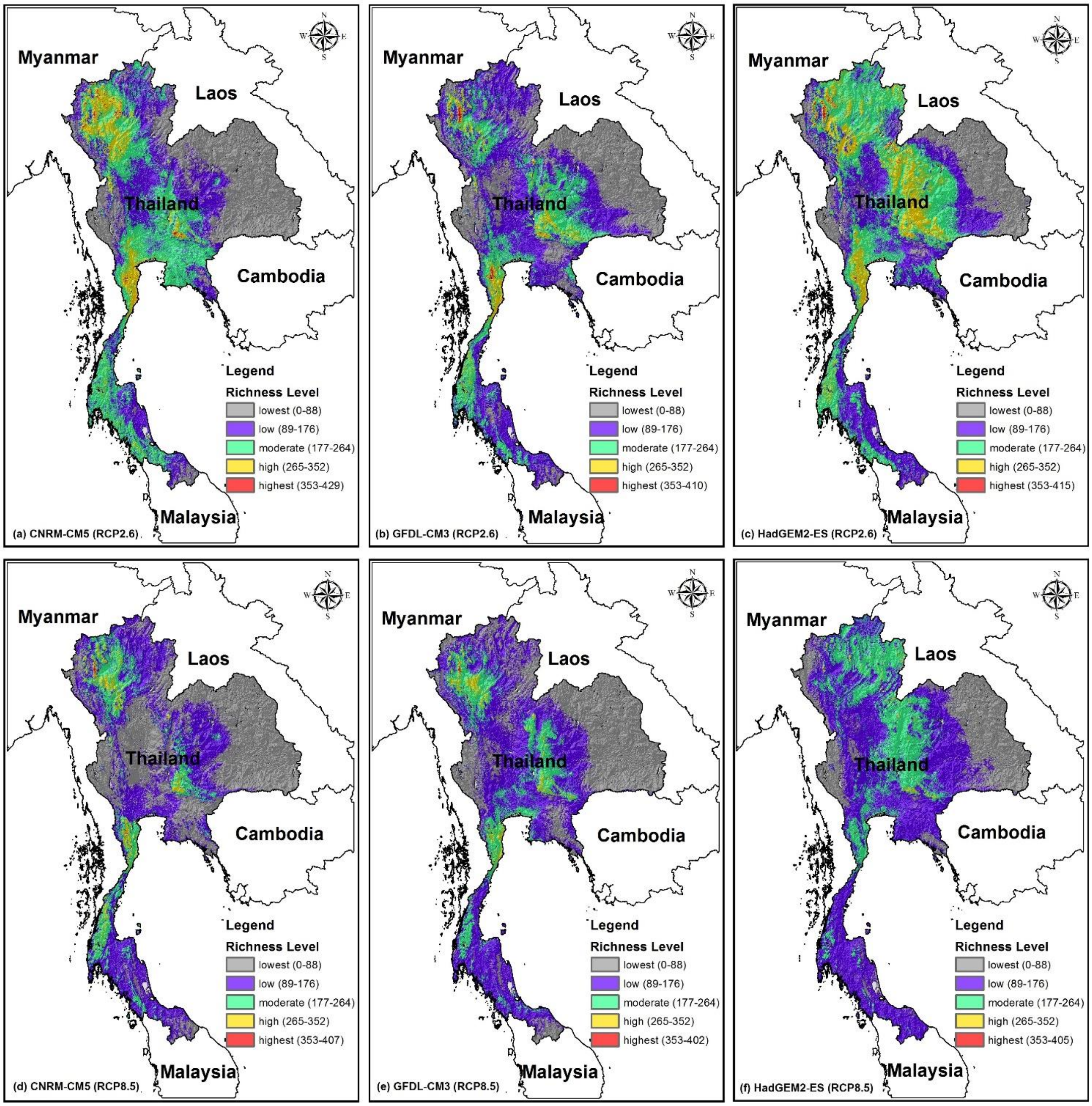


**Fig. S2** Spatial patterns of predicted species richness levels for birds in 2070 with three earth system models and two RCPs. Maps created in ArcMap 10.5 (https://support.esri.com/en/products/desktop/arcgis-desktop/arcmap/10-5-1).

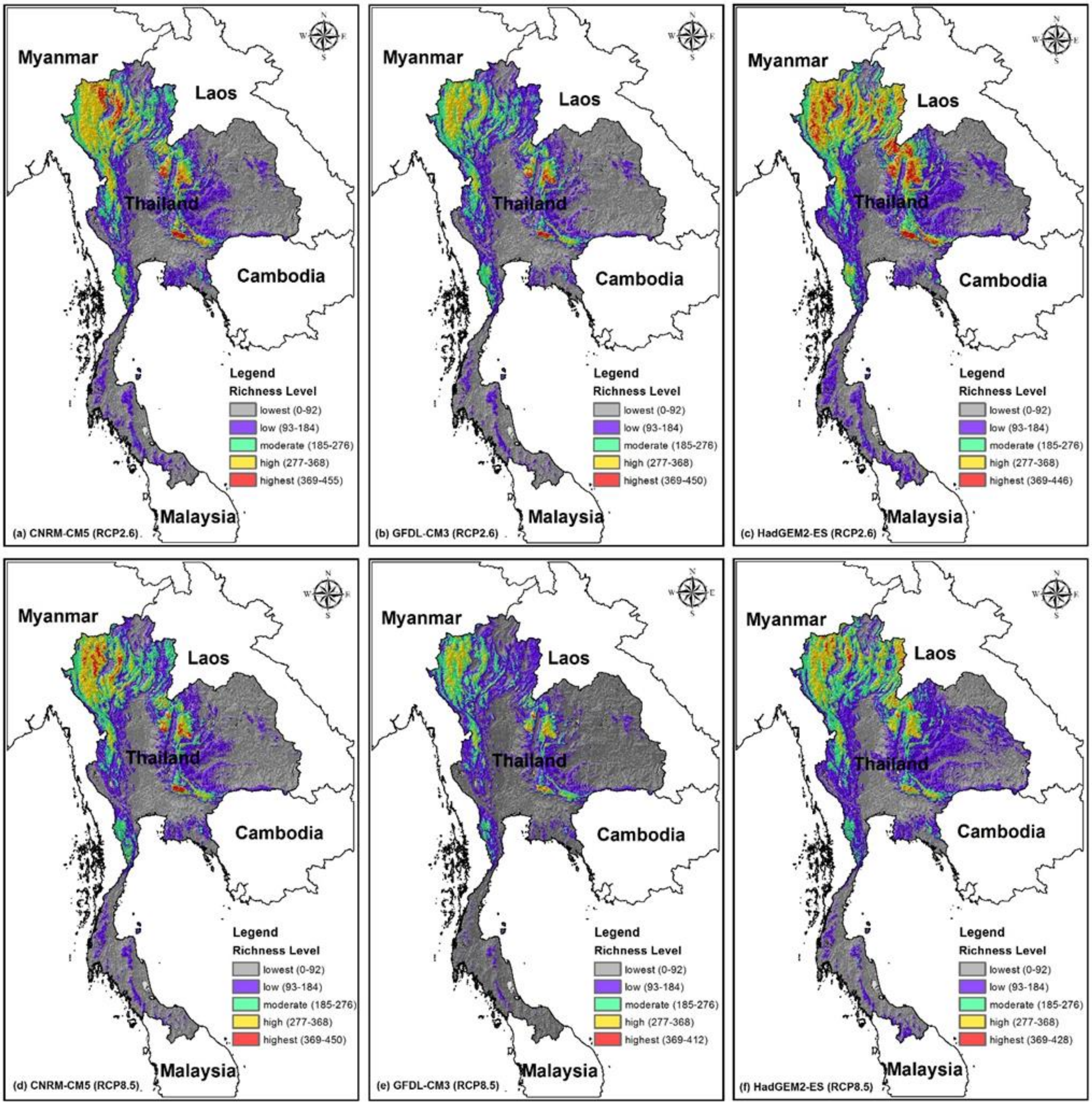


**Fig. S3** Spatial patterns of predicted species richness levels for plants in 2070 with three earth system models and two RCPs. Maps created in ArcMap 10.5 (https://support.esri.com/en/products/desktop/arcgis-desktop/arcmap/10-5-1).

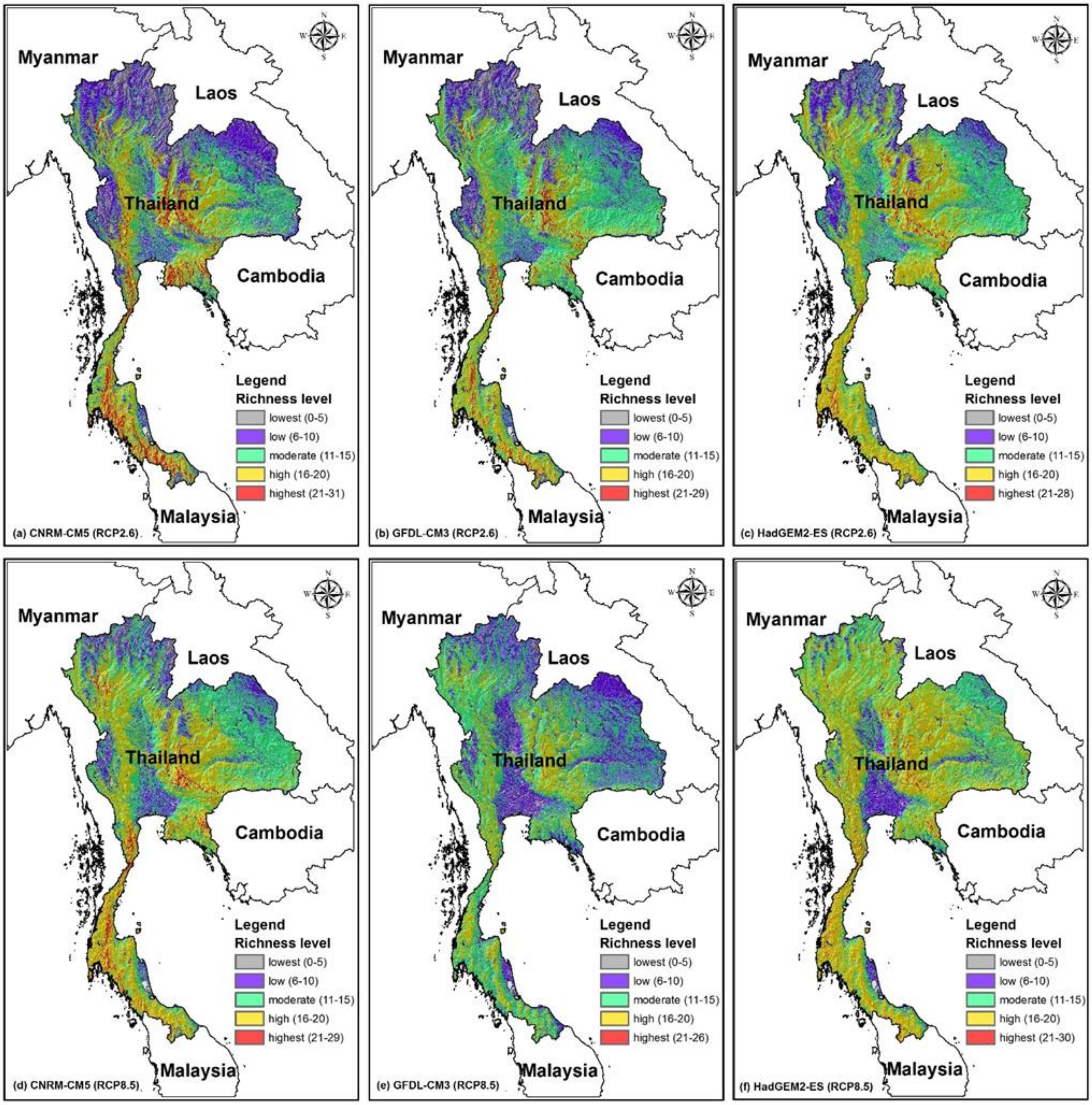


**Fig. S4** Spatial patterns of predicted species richness levels for amphibians in 2070 with three earth system models and two RCPs. Maps created in ArcMap 10.5 (https://support.esri.com/en/products/desktop/arcgis-desktop/arcmap/10-5-1).

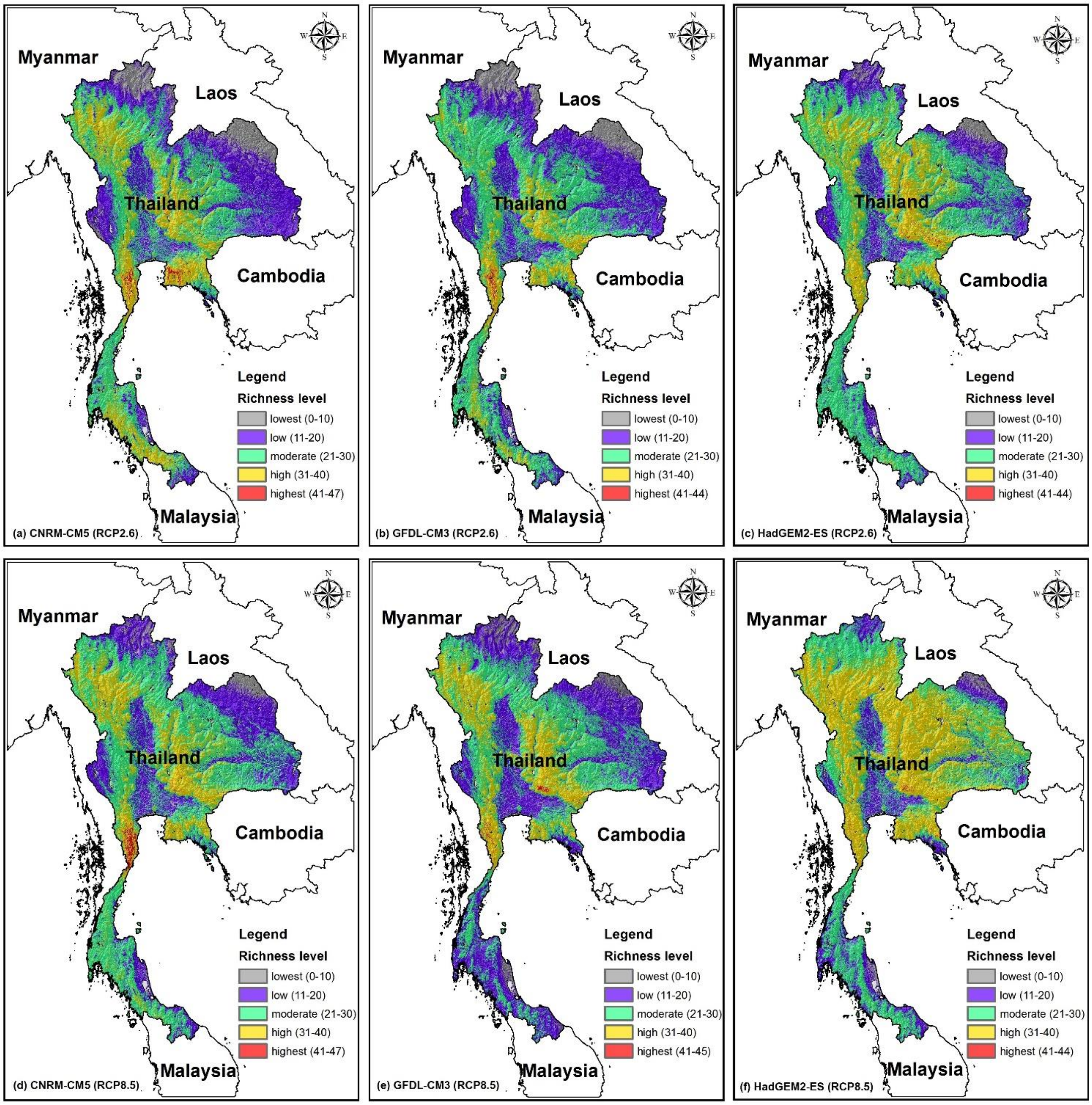


**Fig. S5** Spatial patterns of predicted species richness levels for reptiles in 2070 with three earth system models and two RCPs. Maps created in ArcMap 10.5 (https://support.esri.com/en/products/desktop/arcgis-desktop/arcmap/10-5-1).

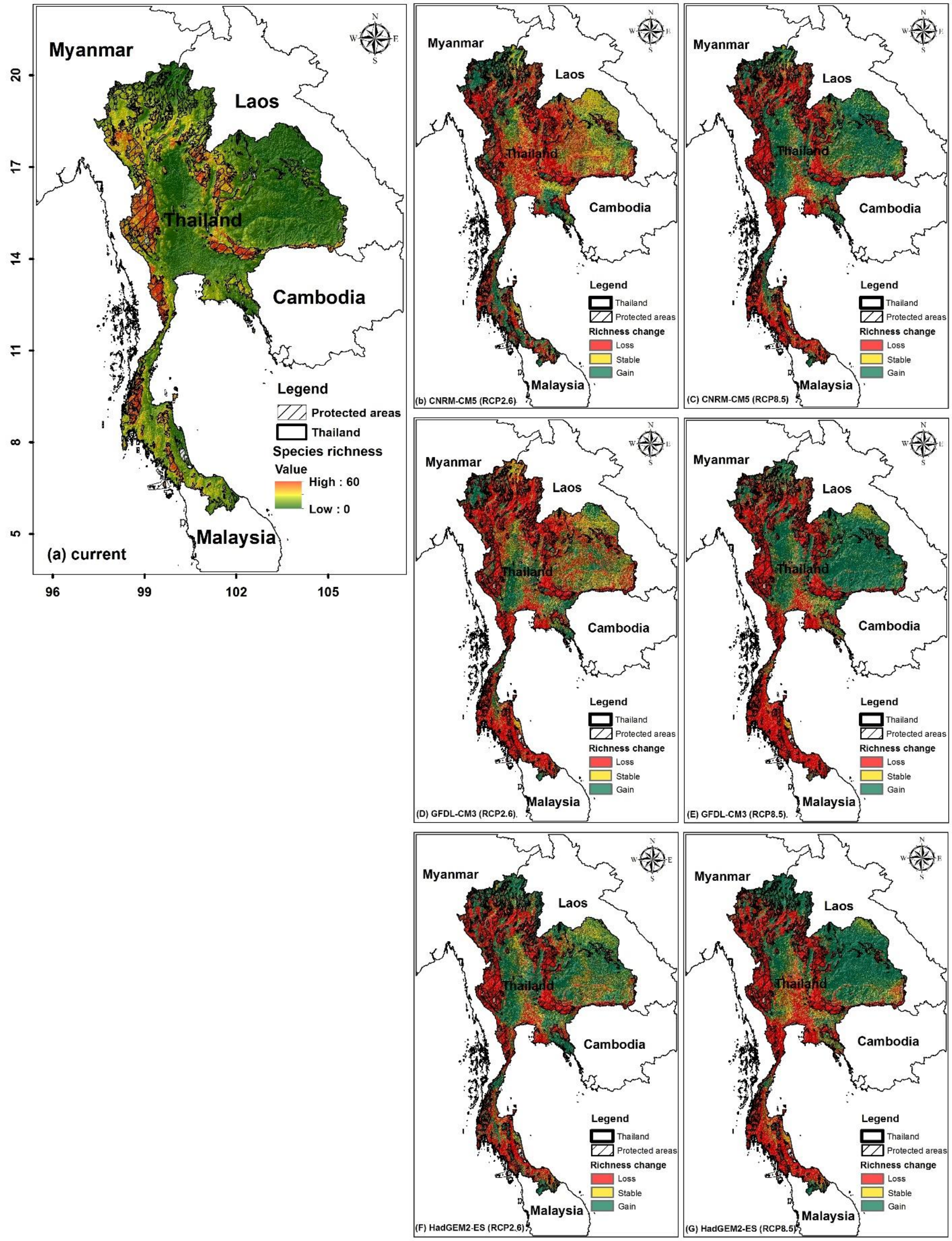


**Fig. S6** Predicted change in species richness levels for mammals by 2070 with three earth system models and two RCPs. Maps created in ArcMap 10.5 (https://support.esri.com/en/products/desktop/arcgis-desktop/arcmap/10-5-1).

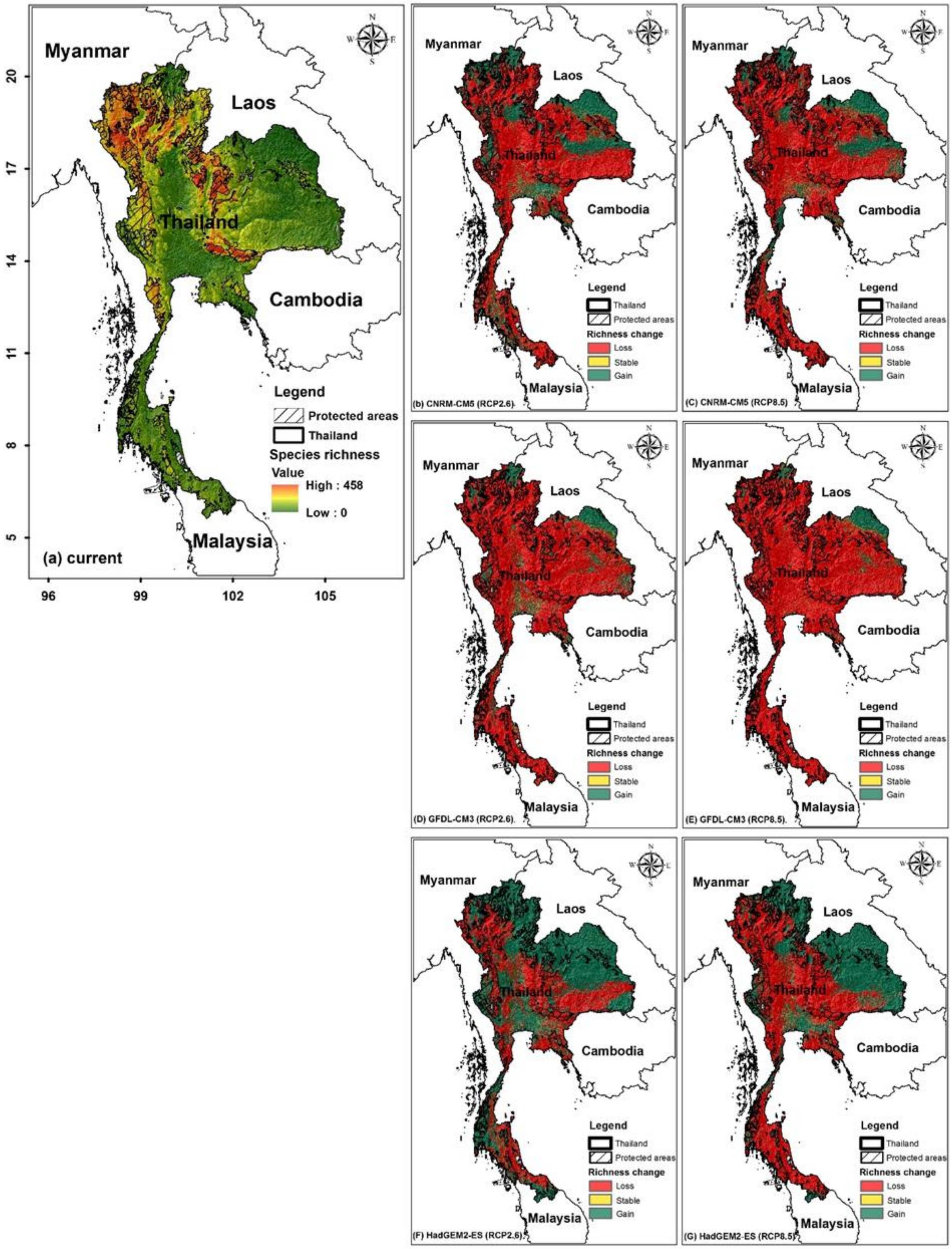


**Fig. S7** Predicted change in species richness levels for plants by 2070 with three earth system models and two RCPs. Maps created in ArcMap 10.5 (https://support.esri.com/en/products/desktop/arcgis-desktop/arcmap/10-5-1).

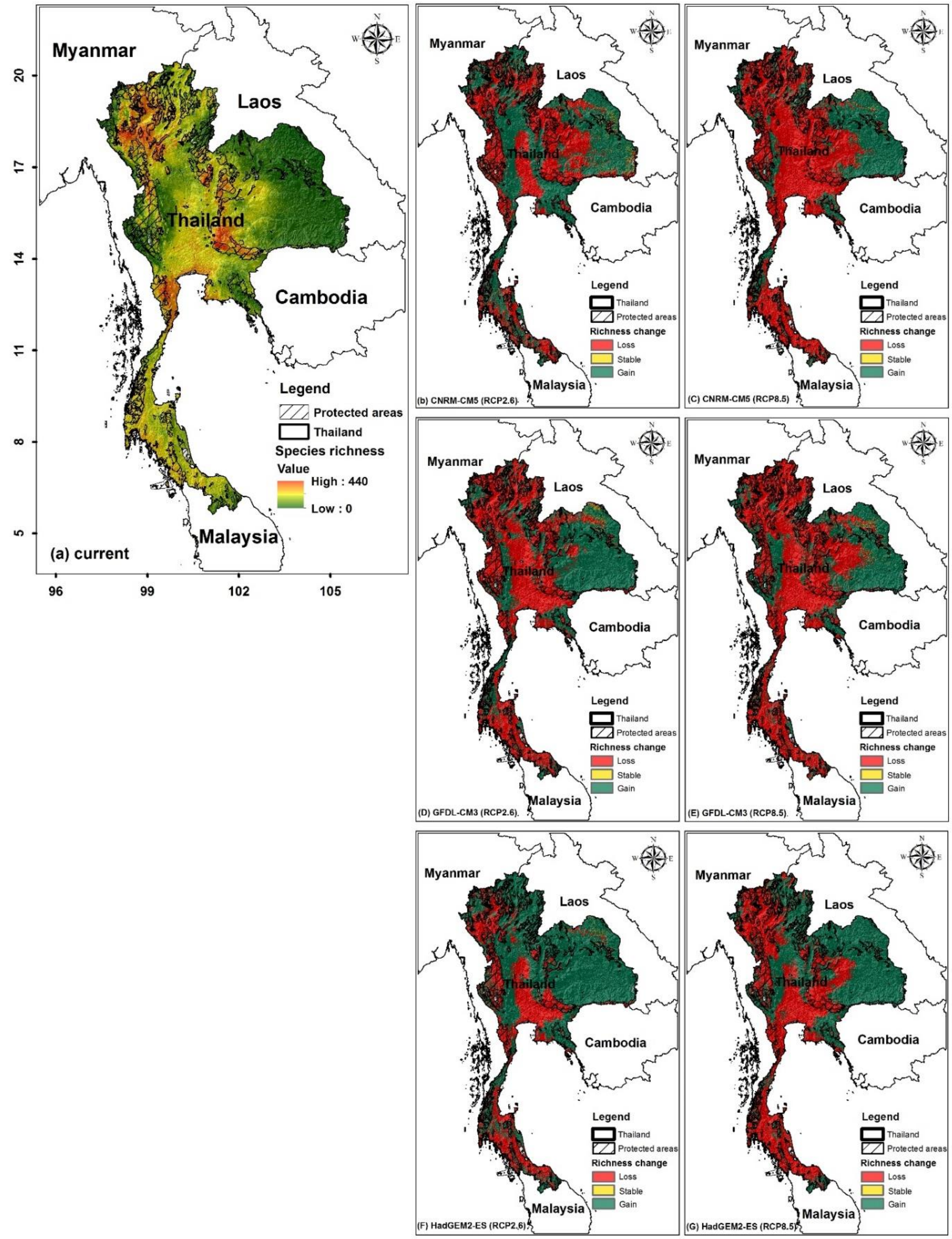


**Fig. S8** Predicted change in species richness levels for birds by 2070 with three earth system models and two RCPs. Maps created in ArcMap 10.5 (https://support.esri.com/en/products/desktop/arcgis-desktop/arcmap/10-5-1).

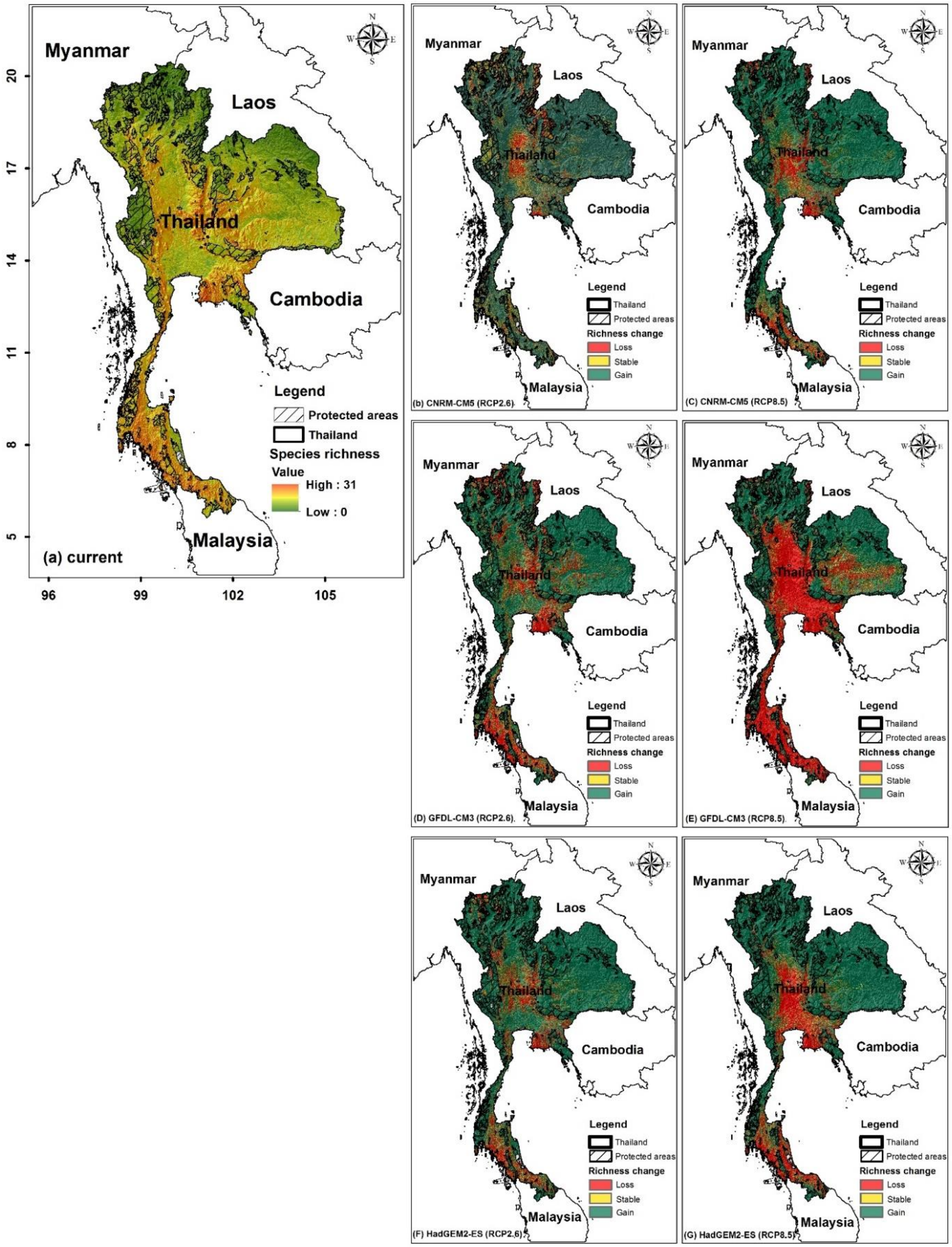


**Fig. S9** Predicted change in species richness levels for amphibians by 2070 with three earth system models and two RCPs. Maps created in ArcMap 10.5 (https://support.esri.com/en/products/desktop/arcgis-desktop/arcmap/10-5-1).

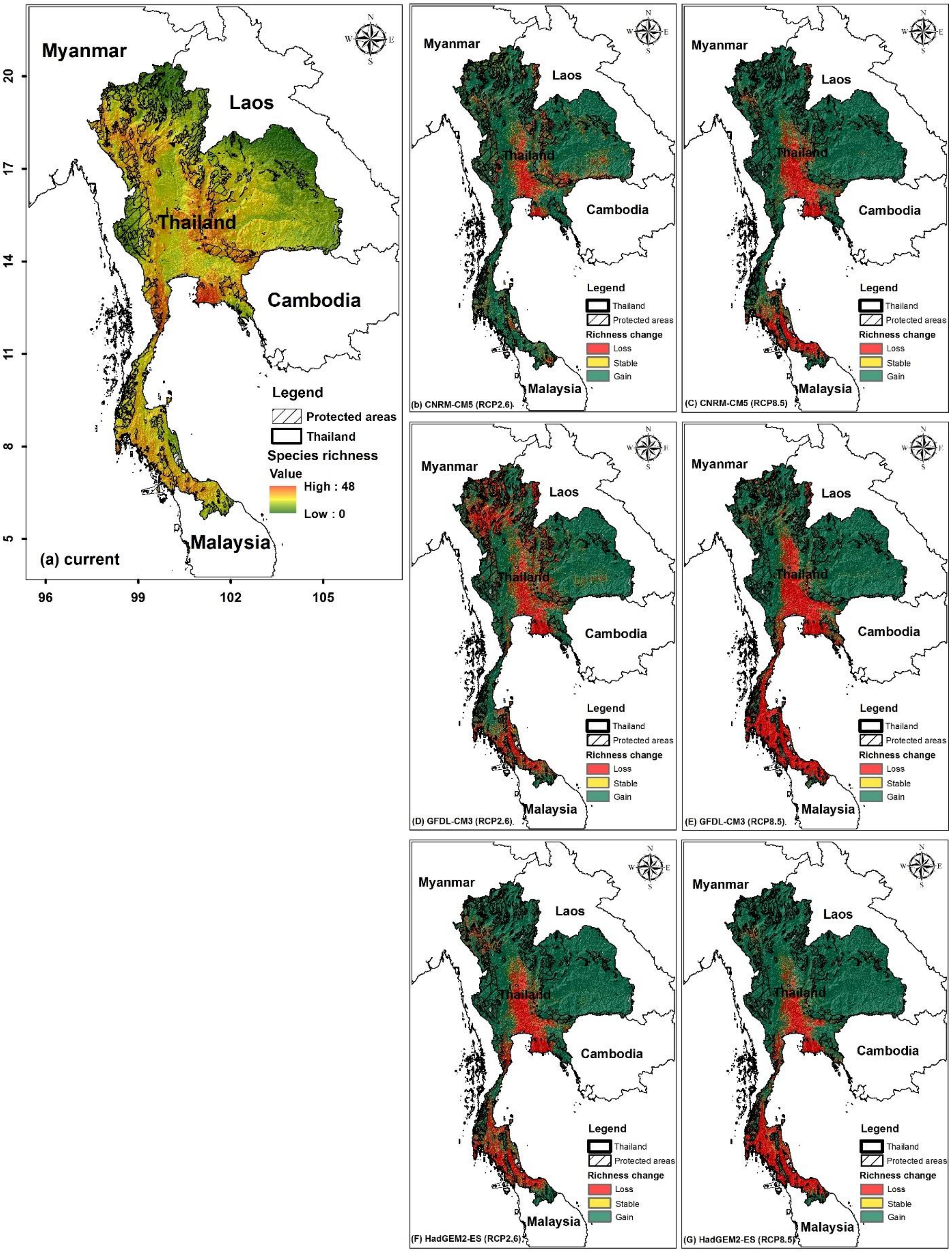


**Fig. S10** Predicted change in species richness levels for reptiles by 2070 with three earth system models and two RCPs. Maps created in ArcMap 10.5 (https://support.esri.com/en/products/desktop/arcgis-desktop/arcmap/10-5-1).
